# Ten sessions of hyperbaric oxygen versus sham treatment in patients with long covid (HOT-LoCO): a randomised, placebo-controlled, double-blind, phase II trial

**DOI:** 10.1136/bmjopen-2024-094386

**Published:** 2025-04-14

**Authors:** Anders Kjellberg, Adrian Hassler, Emil Boström, Sara El Gharbi, Sarah Al-Ezerjawi, Anna Schening, Katarina Fischer, Jan H Kowalski, Kenny A Rodriguez-Wallberg, Judith Bruchfeld, Marcus Ståhlberg, Malin Nygren-Bonnier, Michael Runold, Peter Lindholm

**Affiliations:** 1 Department of Physiology and Pharmacology, Karolinska Institutet, Stockholm, Sweden; 2 Perioperative Medicine and Intensive Care, Medical Unit Intensive Care and Thoracic Surgery, Karolinska University Hospital, Stockholm, Sweden; 3 Medical Unit Emergency Medicine, Karolinska University Hospital, Stockholm, Sweden; 4 Department of Medicine Solna, Karolinska Institutet, Stockholm, Sweden; 5 Medical Unit Cardiology, Heart and Vascular Center, Karolinska University Hospital, Stockholm, Sweden; 6 JK Biostatistics AB, Stockholm, Sweden; 7 Department of Oncology and Pathology, Karolinska Institutet, Stockholm, Sweden; 8 Department of Reproductive Medicine, Karolinska University Hospital, Stockholm, Sweden; 9 Department of Infectious Diseases, Karolinska University Hospital, Stockholm, Sweden; 10 Department of Neurobiology, Care Sciences and Society, Karolinska Institutet, Stockholm, Sweden; 11 Women’s Health and Allied Health Professionals Theme, Medical Allied Health Professionals, Karolinska University Hospital, Stockholm, Sweden; 12 Department of Medicine Solna, Respiratory Medicine Unit, Karolinska Institutet, Stockholm, Sweden; 13 Department of Respiratory Medicine and Allergy, Karolinska University Hospital, Stockholm, Sweden; 14 Department of Emergency Medicine, Division of Hyperbaric Medicine, University of California San Diego, La Jolla, California, USA

**Keywords:** Post-Infectious Disorders, Post-Acute COVID-19 Syndrome, Public health, Clinical trials, Randomized Controlled Trial

## Abstract

**Objectives:**

To evaluate if 10 sessions of hyperbaric oxygen treatments (HBOTs) improve short- and long-term health related quality of life, symptoms and physical performance in long covid patients compared with placebo.

**Design:**

Parallel, randomised, placebo-controlled, double-blind trial.

**Setting:**

Single-centre, university hospital, Sweden.

**Participants:**

Previously healthy subjects aged 18–60 years, diagnosed with long covid were included. We excluded pregnant women, patients with RAND-36 (role limitations due to physical health (RP) and physical functioning (PF)) above 70, diabetes, hypertension and contraindications for HBOT.

**Interventions:**

Subjects were randomly assigned to 10 sessions of HBOT or sham (placebo) treatments over 6 weeks. HBOT involved 100% oxygen, 2.4 bar, 90 min, placebo medical air, 1.34–1.2 bar. Randomisation (1:1) was done electronically, in blocks stratified by sex and disease severity. Subjects and investigators were blinded to allocation.

**Primary and secondary outcome measures:**

Primary endpoints were changes from baseline in RAND-36 PF and RP at 13 weeks. Efficacy was analysed on an intention-to-treat basis. Harms were evaluated according to the actual treatment given.

**Results:**

Between 15 September 2021 and 20 June 2023, 80 subjects (65 women, 15 men) were enrolled and randomised (40 in each group). The trial is completed. The primary endpoint analysis included 79 subjects (40 in HBOT and 39 in control). At 13 weeks, both groups showed improvement, with no significant difference between HBOT and placebo in PF (least square mean difference between groups (LSD), 0.63 (95% CI −7.04 to 8.29), p=0.87) and RP (LSD, 2.35 (95% CI −5.95 to 10.66), p=0.57). Harms: 43 adverse events (AEs), most commonly cough and chest pain/discomfort, occurred in 19 subjects (49%) of the HBOT group and 38 AEs in 18 subjects (44%) of the placebo group, one serious AE in HBOT and one death in the placebo group.

**Conclusions:**

10 HBOT sessions did not show more short-term benefits than placebo for long covid patients. Both groups improved, with a notable sex difference. HBOT has a favourable harm profile.

**Trial registration number:**

ClinicalTrials.gov (NCT04842448), EudraCT (2021-000764-30). The trial was funded by Vetenskapsrådet (2022-00834), Region Stockholm (2020-0731, 2022-0674), Hjärt-Lungfonden and OuraHealth Oy.

**STRENGTHS AND LIMITATIONS OF THIS STUDY**

- The present randomised, placebo-controlled, double-blind clinical trial was conducted in compliance with International Council for Harmonisation of Technical Requirements for Pharmaceuticals for Human use-Good Clinical Practice, the protocol was published at the start of the trial, and we report benefits and harms.
- The trial was designed and conducted with a 1-year parallel follow-up.
- The randomisation was stratified for sex category to allow for subgroup analysis.
- The hypothesis for the treatment protocol was based on early reports in 2021, indicating benefits from only 10 sessions of hyperbaric oxygen treatments for post-COVID-19 condition.

## Introduction

 Some 80 million people are estimated to suffer from persistent symptoms and low health related quality of life (HRQoL) after COVID-19. The estimation is based on close to 800 million confirmed cases of COVID-19 reported to date and 10% of infected individuals suffering persistent symptoms even after mild acute infections.^1^ Post-COVID-19 condition, also known as long covid, is defined by WHO as the continuation or development of new symptoms 3 months after the initial SARS-CoV-2 infection, with these symptoms lasting for at least 2 months with no other explanation.^2^ More than 200 symptoms have been reported, but some of the most common are fatigue, postexertional malaise (PEM), cognitive dysfunction (‘brain fog’), cough, dyspnoea, palpitations and pain.^1 3^ The understanding of underlying mechanisms is scarce, but suggested hypotheses include abnormal immune response (including autoantibodies and dysregulated T cell activation), viral persistence, chronic oxidative stress, mitochondrial dysfunction and endothelial dysfunction.^4^ Some ongoing trials have been criticised for not addressing the possible background mechanisms and not involving patient representatives in the trial designs.^5^ Despite extensive efforts to create high-grade evidence, current guidelines are mainly based on expert opinion.^4^ No effective evidence-based pharmacological treatment options for the underlying condition have been widely adopted into clinical practice,^6^ and many patients seek expensive and potentially harmful ‘remedies’ for self-management.^7^ Hyperbaric oxygen treatment (HBOT) has 14 internationally approved indications with demonstrated potential in a multitude of applications in inflammatory and systemic conditions but remains controversial despite half a century of clinical use.^8 9^ HBOT with 40 sessions at 2.0 atmospheres absolute (ATA), 90 min with 5 min air breaks every 20 min has been shown to improve neurocognitive function and symptoms. Clinical improvement was associated with changes on MRI and improvement of myocardial function in long covid in a randomised, placebo-controlled trial.^10 11^ A longitudinal follow-up of selected subjects from the phase II trial suggests a sustained effect for 3 months but high-grade evidence for long-term efficacy compared with a control group is missing.^12^ HBOT has previously been suggested to be effective in chronic fatigue syndrome with only 15 sessions.^13^ Two case series with 32 patients suggest efficacy from 10 sessions at 2.2–2.4 ATA, 75–105 min with two 5 min air breaks, but no Randomised Controlled Trials (RCTs) are published on this dose. A shorter treatment with 10–15 sessions has become increasingly popular off-label for long covid.^14^ The rationale for using fewer and less frequent sessions for long covid is based on the hyperoxic–hypoxic paradox with downstream regulation of hypoxia and inflammatory pathways,^15^ previous clinical experience from severe COVID-19 and experimental research.^16 17^ The safety profile of HBOT is well known for accepted indications, but has not been described in compliance with International Council for Harmonisation of Technical Requirements for Pharmaceuticals for Human use-Good Clinical Practice (ICH-GCP) and is not an accepted treatment for patients diagnosed with long covid.^18^ One of the reasons why HBOT is not generally accepted is the lack of consensus between caregivers, competent authorities and researchers regarding HBOT as a pharmacological or complementary intervention.^19^ Hence, HBOT is often overlooked in systematic reviews and other review articles on pharmacological interventions, despite preregistered as clinical trials.^20^ The aim of this trial was therefore to evaluate if 10 sessions of HBOT improve HRQoL, symptoms and objective findings for patients with long covid, to evaluate potential harms and to explore underlying mechanisms in compliance with ICH-GCP.

## Materials and methods

### Trial design

HOT-LoCO (hyperbaric oxygen for treatment of long covid syndrome) is an investigator-initiated, randomised, placebo-controlled, double blind, parallel-arms, phase II clinical trial that was conducted at the hyperbaric outpatient clinic at Karolinska University Hospital, Stockholm, Sweden.

The protocol was approved by the Swedish ethical review board (2021-02634, amendment 2021-04572, approval date 25 May 2021 and 22 September 2021) and the Swedish medical products agency (5.1-2020-36673, approval date 06 July 2021). The trial was registered on ClinicalTrial.gov (NCT04842448), 13 April 2021, and on EudraCT (2021-000764-30), 21 May 2021, before the start of the trial. Patient representatives from the post-COVID association in Sweden (Svenska Covidföreningen) were involved and approved the trial design. The protocol includes a detailed description and rationale for the primary and main secondary endpoints, including patient reported outcomes (PRO) in line with Standard Protocol Items: Recommendations for Interventional Trials (SPIRIT) SPIRIT-PRO Extension guidelines.^21^ The full protocol is published and available with open access.^22^ The trial was conducted in accordance with The Declaration of Helsinki, ICH-GCP, local and national regulations and is reported according to Consolidated Standards of Reporting Trials (CONSORT) Harms 2022 guidelines.^23^ The trial was monitored by an independent monitoring organisation, Karolinska Trial Alliance (KTA), before, during and after the trial according to the monitoring plan. An independent data safety monitoring board (DSMB) reviewed the data three times during the trial. A charter delineating their operating guidelines and stopping rules for terminating individual subjects, a portion or all the trial prematurely, was drawn up and agreed on before the trial started. The DSMB is composed of three experts in their respective disciplines of medicine, clinical trial methodology and conduct. The members of the DSMB, meeting plan and responsibilities are specified in the original protocol (p6 and 44).

### Patients

Patients were recruited through long covid outpatient clinics, directly or by advertisement through Svenska Covidföreningen. Inclusion criteria were: aged 18–60 years, previously generally healthy (defined as American Society of Anesthesiologists (ASA) classes I and II), symptoms consistent with long covid for a minimum of 12 weeks, diagnosed with post-COVID-19 condition (International Statistical Classification of Diseases and Related Health Problems – Tenth Revision (ICD-10) code U09.9), working or studying prior to COVID-19, documented informed consent according to ICH-GCP and national regulations. Exclusion criteria were: pregnancy or positive pregnancy test in women of childbearing age, ASA class III or more from other causes than long covid, score above 70 in RAND-36 domain physical functioning (PF) or role limitations due to physical health (RP), diabetes, diagnosed with hypertension prior to COVID-19, contraindication for HBOT according to local guidelines, participation or recent participation in a clinical trial with an investigational product, mental inability, reluctance or language difficulties that result in difficulty understanding the meaning of trial participation.

### Randomisation and masking

Eligible subjects were randomised in a 1:1 allocation, stratified by disease severity in relation to RAND-36 domains PF and PR (mean of PF and RP <30 or >30) and sex in mixed block sizes 2:4:8 (blinded to investigators and personnel assessing outcome data) to either HBOT or placebo. Both interventions included air breaks with medical air delivered by a non-rebreather mask. The random allocation sequence was generated by the trial statistician with a computer-based tool (randomizer.at), and only delegated staff specifically involved in the treatment and an unblinded monitor had access to the code. The standard operating procedure with details about the randomisation–blinding procedure used is published with the protocol and available online.^22^ The placebo protocol is well established, and even experienced divers cannot differentiate between ‘sham treatment’ and HBOT.^24^ The success of the masking procedure was validated by asking the subjects to guess the allocated treatment after the first treatment. The masking procedures were monitored by an independent unblinded monitor from KTA.

### Trial procedures

**Figure 1:**
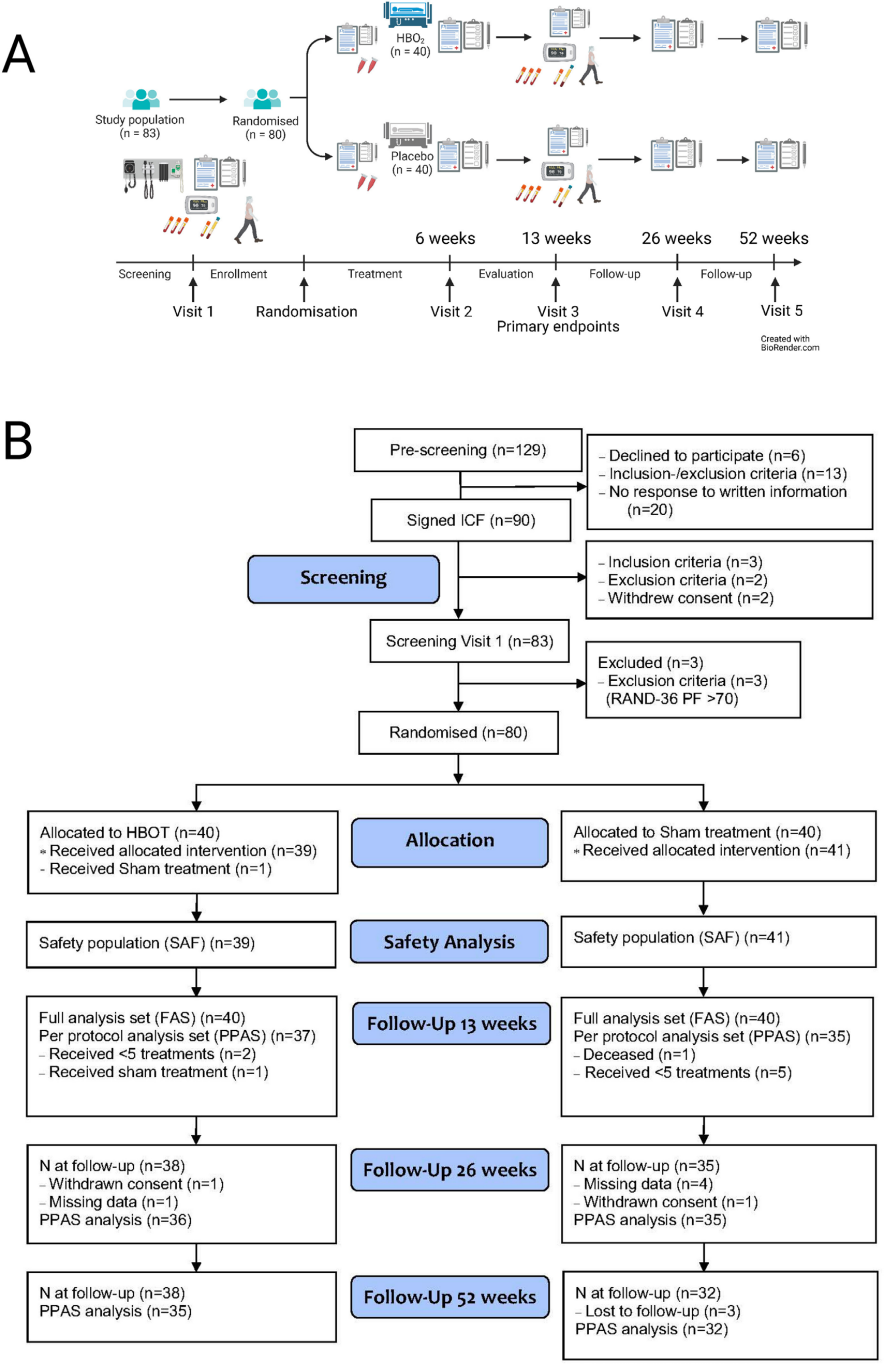
Trial overview/panel A shows the trial design. The trial involved five visits over 52 weeks. Panel B shows the CONSORT flow diagram. 129 patients were assessed for eligibility, 90 patients signed an informed consent form (ICF), 83 patients were screened, 80 subjects were randomised, 40 in each group. One subject in the HBOT group received sham treatment. CONSORT, Consolidated Standards of Reporting Trials; HBOT, hyperbaric oxygen treatments; PF, physical functioning.

The trial consisted of five visits over 52 weeks. Assessments at visits 1 and 3 were identical and included medical history, concomitant medication, physical examination, blood tests including biobanking, questionnaires RAND-36, EuroQol-5 Dimensions (EQ-5D), physical tests including the 6-minute Walk Test (6MWT), the 30/60-second Chair-Stand Test (CST) and other objective evaluations, including endothelial function with pulse amplitude tonometry, measurements of cardiac function (Nexfin) and activity, heart rate variability and sleep patterns with an activity metre (Oura ring). Eligible subjects at visit 1 were randomised and received a maximum of 10 HBOT or placebo (sham) treatments over 6 weeks from randomisation, the recommended scheme was every 2–3 days. Medical oxygen 100% was administered at 2.4 ATA for 90 min with two 5 min air breaks (rebreather mask). Sham treatment with medical air was administered by increasing pressure briefly to 1.34 ATA and then reduced and maintained at 1.2 ATA for 90 min with two 5 min air breaks (rebreather mask). All treatments were given in monoplace chambers (Sechrist, USA). Visit 2 was conducted in conjunction with the last treatment and included RAND-36, EQ-5D, physical examination and reporting of adverse events (AEs). The primary, main secondary endpoints and harms were assessed at 13 weeks from randomisation at visit 3. Visits 4 and 5 were long-term follow-up for exploratory endpoints and health-economic evaluation. A flow chart of the trial design and the CONSORT flow diagram is depicted in figure 1. A detailed description of all procedures is available in the protocol.^22^

### Outcomes

The primary endpoints were the physical domains, PF and RP, in the RAND 36-Item Health Survey 1.0 at 13 weeks. The main secondary endpoints were the physical tests 6MWT and 30-seconds CST, European Quality of Life-5 Dimensions-5 Level version (EQ-5D-5L) and objective evaluation of endothelial function with reactive hyperaemia index at 13 weeks. Harm endpoints were occurrence, frequency and seriousness of AEs.^22^ The definition, handling, follow-up and reporting of AEs are defined in the original protocol (p34–38). In short, AE data were collected directly after inclusion and continued to week 13, which is 7 weeks after the subjects have ended their treatment with the investigational product. All AEs were registered during the treatment period, and only serious adverse events (SAEs) were registered from week 6 until week 13. The benefits and harms were evaluated by an independent DSMB in the context of the trial design and of current information about long covid and HBOT.

### Statistical analysis

The primary endpoint is the mean change from baseline to week 13 in the RAND-36 score of RP and PF, respectively. A 10-point higher mean change in the HBOT group compared with the placebo group was considered as a clinically relevant difference. Sample size calculation was performed using a t-test for independent groups with 80% power, and with a type I error rate of 0.05 (5%), assuming a common SD of 15 known from prior studies, to detect a 10-point difference between groups. Power calculations indicated that at least 37 subjects per group were needed. Accounting for 5% dropout, we aimed to recruit 80 subjects. nQuery V.7 was used for sample size calculations.

In general, for continuous outcome variables including the primary endpoint, analyses were performed using analysis of covariance, unless otherwise specified, including stratification factors and treatment as fixed factors in the model. Estimates are presented using least square means for differences between treatment arms.

#### Hypothesis testing and adjustment for multiplicity

The null hypothesis tested was that there is no difference between HBOT and placebo (H_0_: HBOT=placebo), in the mean change from baseline to week 13 in the RAND-36 RP and PF, respectively. The same statistical hypothesis was used for the main secondary endpoints. Hypothesis testing for the primary endpoints was controlled at the type I error rate of 0.05 where adjustment for multiplicity was done using the Hochberg method, comparing the largest p value, when testing the mean change in the RAND-36 RP and PF, against the significance level 0.05, and the smallest p value against the significance level 0.025. Further, there was no adjustment for multiplicity in secondary endpoints as this is an exploratory study, but nominal p values are presented, and results are interpreted as exploratory findings. All hypothesis tests were two-sided.

#### Analysis populations

The full analysis set (FAS) population includes all randomised subjects who were exposed at least once to the study intervention. The FAS subjects were analysed according to their randomised/assigned treatment irrespective of which treatment they actually received. The per-protocol analysis set (PPAS) population includes all FAS subjects with no major protocol violations and who received at least five treatment sessions, and per-protocol set 10 (PPS10) includes all FAS subjects with no major protocol violations and who received all 10 treatment sessions. The Safety population (SAF) includes all randomised subjects who have received at least one study treatment. The SAF patients were analysed according to their actual treatment received. Primary and main secondary endpoints were evaluated using the FAS population, and sensitivity analyses were performed using the PPAS population. For all analyses, the observed population has been used for evaluation, that is, no imputation for missing data. It was only one subject (who deceased) missing at the evaluation for the primary endpoint in the FAS population.

#### Subgroups

Subgroup analysis was done for males and females.

#### Statistical methodology

The primary objective of the study was to confirm the superior efficacy of the HBOT treatment compared with placebo in the primary endpoints.

All continuous variables are described using standard statistical measures, that is, number of observations (N), percentage (%), mean or median value, SD, and minimum and maximum values.

Analysis for categorical data (yes/no) is presented as the proportion of subjects with the frequency of presence or absence, by treatment group of the characteristics of interest, and analysed using the Cochran-Mantel-Haenszel χ^2^ test including stratification factors, where the parameter used for the statistical hypothesis testing will be the OR, as a measure of the relative difference in odds between treatment arms. An OR value greater than one indicates efficacy in favour of HBOT compared with placebo. The McNemar’s test was used to assess the association between the guessed and actual treatments (eg, proportion of correct versus incorrect guesses). A high p value suggests that the blinding procedure was effective.

### Role of the funding source

The funders of the trial had no role in the trial design, data collection, data analysis, data interpretation or writing of the report.

### Patient and public involvement

The trial design and consent form were discussed with and approved by a patient representative. We thank Svenska Covidföreningen through former chairman Åsa Kristofferson-Hedlund for their support.

## Results

Between 15 September 2021 and 20 June 2023, 80 subjects were randomised. 61 subjects completed their 10 treatments (29 in HBOT and 32 in placebo), four subjects had less than five treatments (three in HBOT and four in placebo) because of AEs and 15 subjects had five to nine treatments (seven in HBOT and eight in placebo), one subject in the HBOT group was given placebo.

All subjects completed the 13-week follow-up, with the exception of one subject who died in the placebo group. Two subjects withdrew consent and seven were lost to follow-up, the remaining 70 had completed 52 weeks of follow-up (visit 5) by 17 June 2024. The groups were balanced regarding demographics and baseline characteristics, suggesting a successful randomisation (table 1). Mean age was 41 years, body mass index 24, 80% were women, 94% were Caucasian and 81% had a higher education diploma. One subject assigned to HBOT received a placebo by mistake. The treatment was generally well tolerated; 29 subjects (75%) in the HBOT group and 32 subjects in the placebo group (78%) completed all 10 sessions, but very few of our subjects could tolerate more than three treatments per week due to severe PEM. There were no subjects in the highest interval of baseline disease severity, the mean of the Rand-36 physical domains PF and RP above 50, due to the high severity of the disease. Most subjects (66) were in the 0–30 interval, and 14 subjects were in the medium interval of 30–50.

**Table 1:** Demographics and baseline characteristics (n=80)

Demographics	HBOT (n=40)	Placebo (n=40)	Total (n=80)
Age (years)	41.1 (10.14)	41.4 (8.16)	41.3 (9.15)
Female (sex)	32 (80.0%)	33 (82.5%)	65 (81.3%)
Body mass index (kg/m^2^)	24.4 (4.12)	24.3 (4.13)	24.4 (4.10)
Tobacco			
Never smoker	28 (70.0%)	31 (77.5%)	59 (73.8%)
Ex-smoker	12 (30.0%)	9 (22.5%)	21 (26.2%)
Never used Swedish snus (smokeless)	32 (80.0%)	34 (85.0%)	66 (82.5%)
Ex-user Swedish snus	2 (5.0%)	5 (12.5%)	7 (8.8%)
Casual user Swedish snus	2 (5.0%)	0 (0.0%)	2 (2.5%)
Daily user Swedish snus	4 (10.0%)	0 (0.0%)	4 (0.5%)
Full time work/study before COVID-19	40 (100%)	35 (87.5%)	75 (93.0%)
Full time work/study at baseline	4 (10.0%)	5 (12.5%)	9 (11.2%)
Education (diploma/university degree)	31 (77.5%)	34 (85.0%)	65 (81.2%)
Physical activity, exercise (min/week)	23.5 (54.6)	14.3 (30.8)	18.9 (44.3)
Fully vaccinated	29 (72.5%)	26 (65.0%)	55 (68.8%)
Time from COVID-19 onset (months)	26.2 (7.1)	25.1 (8.7)	25.6 (7.9)
Positive SARS-CoV-2 PCR	16 (40.0%)	14 (35.0%)	30 (37.5%)
Positive SARS-CoV-2 antibodies	23 (57.5%)	18 (45.0%)	41 (51.2%)
RAND-36 domains (points)			
Physical functioning	39.8 (18.6)	37.9 (19.0)	38.8 (18.7)
Role limitations due to physical health	0.6 (4.0)	0.0 (0.0)	0.3 (2.8)
General health	28.8 (14.0)	25.0 (11.0)	25.0 (11.0)
Mental health	61.1 (16.6)	60.0 (16.5)	60.6 (16.5)
Role emotional	65.0 (45.3)	58.4 (45.2)	61.7 (45.1)
Social functioning	21.4 (22.5)	22.4 (20.5)	21.9 (21.4)
Body pain	51.6 (26.4)	51.6 (24.9)	51.6 (25.5)
Vitality	19.1 (19.1)	14.8 (12.1)	16.9 (16.0)
EQ-5D			
EQ-5D (VAS)	38.4 (15.1)	39.5 (16.8)	38.9 (15.9)
EQ-5D-5L (index)	0.5 (0.2)	0.5 (0.2)	0.5 (0.2)
Physical tests			
Reactive hyperaemia index	2.1 (0.6)	2.0 (0.6)	2.0 (0.6)
Cardiac index (Nexfin) (L/min/m^2^)	3.14 (0.84)	3.20 (0.76)	3.17 (0.80)
30-second chair-stand test (no/30 s)	12.1 (4.4)	11.0 (4.1)	12.0 (4.2)
6-minute walk test (m)	468.8 (129.6)	449.7 (141.8)	459.3 (135.4)
Symptoms			
Fever, chills or sensation of fever	22 (55.0%)	23 (57.5%)	45 (56.2%)
Cough, dyspnoea	28 (70.0%)	27 (67.5%)	55 (68.8%)
Brain fog	33 (82.5%)	37 (92.5%)	70 (87.5%)
Postexertional malaise/fatigue	38 (95.0%)	40 (100.0%)	78 (97.5%)
Palpitations,arrhythmiass	31 (77.5%)	33 (82.5)	64 (80.0)
Loss of smell	4 (10.0%)	4 (10.0%)	8 (10.0%)
Pain, including headache	37 (92.5%)	36 (90.0%)	73 (91.2%)
Gastrointestinal symptoms	23 (57.5%)	23 (57.5%)	46 (57.5%)
Vertigo, dizziness, orthostatism	33 (82.5%)	34 (85.0%)	67 (83.8%)

Patient characteristics, presented as number, (n) and proportion, (%) for categorical variables, and mean±standard deviation (SD)SD for continuous variables.

EQ-5D, European Quality of Life-5 dimensions; EQ-5D-5L, EQ-5D-5 level version; HBOT, hyperbaric oxygen treatment; VAS, Visual Analogue Scale.

**Figure 2:**
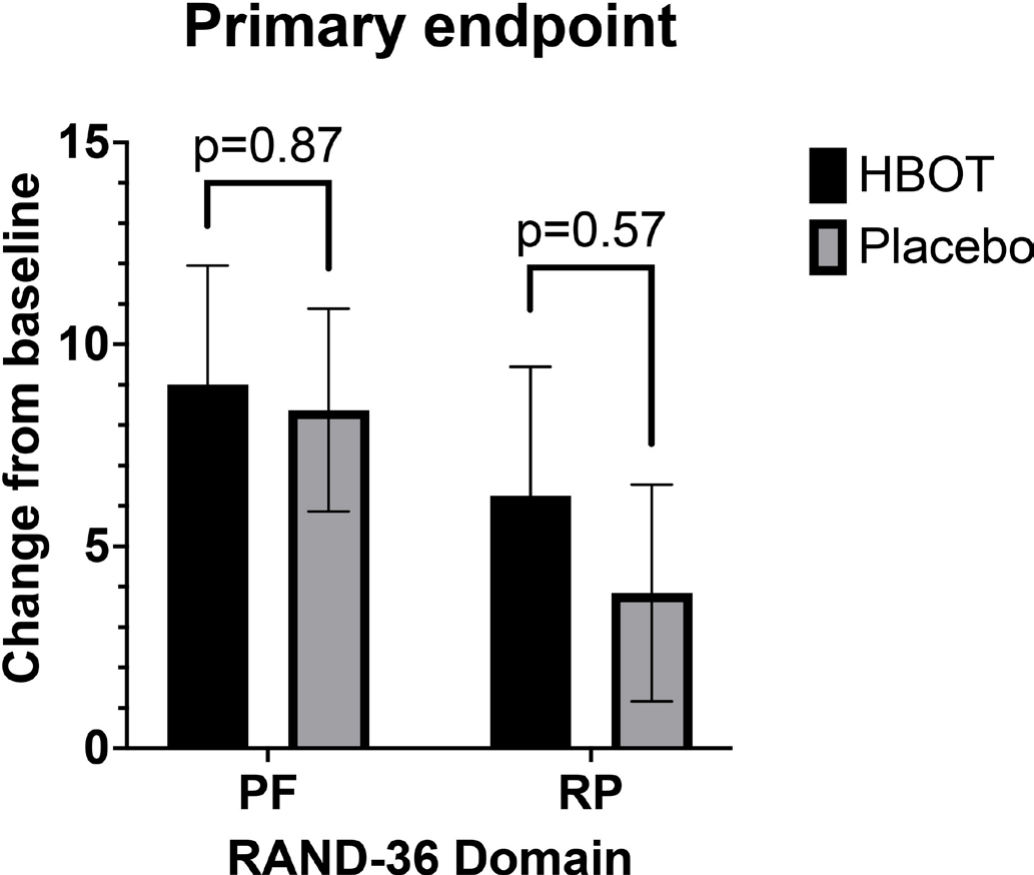
Primary endpoints PF and RP (full analysis set). RAND-36 scores for PF and RP for the respective groups at 13 weeks. Data are presented as mean and whiskers represent SEM. HBOT, hyperbaric oxygen treatment; PF, physical functioning; RP, role limitations due to physical health.

Primary endpoint, mean change from baseline at 13 weeks in RAND-36 domain, PF was 9 (18.68) in HBOT (n=40) versus 8.59 (16.02) in placebo (n=39), least square mean difference between groups (LSD), 0.63 (95% CI (−7.04 to 8.29)), p=0.87. Mean change from baseline in RAND-36 domain, RP was 6.25 (20.22) in HBOT (n=40) versus 3.85 (16.76) in placebo (n=39), (LSD), 2.35 (95% CI (−5.95 to 10.66)), p=0.57. PF and RP were not statistically significantly different at any time point during follow-up in the FAS population (figure 2).

No statistically significant difference was detected in any of the main secondary endpoints in the FAS population (table 2).

**Table 2:** Main secondary endpoints, mean change from baseline at week 13 (full analysis set population)

Endpoint	Change HBOT(n=40)	Change placebo(n=39)	LSD (SE)	95% CI	P value
RHI	−0.09 (0.62)	−0.06 (0.62)	−0.02 (0.14)	−0.31 to 0.26	0.86
6MWT	15.92 (79.63)	23.23 (49.60)	−7.48 (15.22)	−37.81 to 22.84	0.62
30-seconds CST	0.28 (2.77)	0.71 (2.30)	−0.37 (0.59)	−1.53 to 0.80	0.53
EQ-5D-5L index	0.05 (0.21)	0.10 (0.19)	−0.04 (0.04)	−0.13 to 0.05	0.36
EQ-5D VAS	5.35 (15.57)	6.13 (15.28)	−0.53 (3.50)	−7.49 to 6.44	0.88

Data are presented as mean±SD, least square difference between groups (LSD), 95% confidence interval (CI)CI and p -value.

CST, Chair-Stand Test; EQ-5D, European Quality of Life-5 dimensions; EQ-5D-5L, EQ-5D-5 level version; HBOT, hyperbaric oxygen treatment; 6MWT, 6-Minute Walk Test; RHI, reactive hyperaemia index; VAS, visual analogue scale.

When comparing the proportion of subjects with the reported common symptoms cough, ‘brain-fog’ and PEM between groups in the SAF population, we detected a statistically significant improvement in cough at 26 weeks (p=0.020) but no statistically significant differences in other symptoms or timepoints (online supplemental figure 1).

In the exploratory long-term follow-up, we observed a difference in favour of HBOT compared with placebo in PF at 52 weeks, but the differences were not statistically significant in the PPAS analysis (table 3).

**Table 3:** Long term follow-up, PPAS population

Endpoint RAND-36	Week	Mean (SD) change, HBOT	Mean (SD) change, placebo	LSD (SE)	95% CI	P value
PF	13	12.06 (18.00)	7.90 (16.92)	4.87 (4.44)	−4.02 to 13.76	0.277
RP	13	7.76 (23.24)	4.03 (18.37)	3.36 (5.34)	−7.35 to 14.06	0.533
PF	26	11.96 (18.17)	7.68 (21.92)	4.36 (5.48)	−6.64 to 15.35	0.43
RP	26	6.25 (21.11)	8.04 (21.57)	−1.43 (5.68)	−12.83 to 9.95	0.80
PF	52	18.57 (22.60)	10.80 (20.29)	8.43 (5.85)	−3.32 to 20.18	0.156
RP	52	15.18 (29.92)	11.00 (28.94)	3.26 (8.26)	−13.33 to 19.85	0.694

At week 13: n=40; n=39 (HBOT; placebo), week 26: n=38; n=35, week 52: n=38; n=32.

PPAS = subjects with no major protocol violation and who have received at least five treatments.

HBOT, hyperbaric oxygen treatment; LSD, least square difference between groups; PF, physical functioning domain in RAND-36; PPAS, per-protocol analysis set; RP, role limitations due to physical health domain in RAND-36.

When analysing the clinically relevant improvement of 10 units from baseline for the physical domains in RAND-36, we observed a statistically significant change in PF from baseline (p=0.033) at 52 weeks in the FAS population (table 4).

**Table 4:** Frequency and proportion of subjects with a clinically meaningful improvement in PF and RP (full analysis set)

Endpoint RAND-36	Week	Randomised to treatment				
		HBOT	Placebo	OR	P value*	Total
		n (%)	n (%)			n (%)
PF	13	19 (47.5%)	20 (50.0%)	0.92	0.849	39 (48.8%)
PF	26	20 (52.6%)	19 (54.3%)	0.96	0.902	39 (53.4%)
PF	52	25 (65.8%)	13 (40.6%)	**2.94**	**0.033**	38 (54.3%)
RP	13	6 (15.0%)	3 (7.5%)	2.07	0.349	9 (11.3%)
RP	26	5 (13.2%)	4 (11.4%)	1.16	0.853	9 (12.3%)
RP	52	9 (23.7%)	7 (21.9%)	1.12	0.852	16 (22.9%)

Clinically meaningful improvement from baseline is defined as >10 units increase.

At week 13: n=40; n=39, week 26: n=38; n=35, week 52: n=38; n=32 (HBOT; placebo).

Bold values indicate a statistically significant difference.

* Chi-squareχ2 test, Cochran-Mantel Haenszel test, adjusted for sex category and disease severity. ratio. At week 13: ; , week 26: ; , week 52: ; (HBOT; placebo).

HBOT, hyperbaric oxygen treatment; PF, physical functioning; RP, role limitations due to physical health.

The frequency of AEs was similar in both groups. Two SAEs were reported, one (generalised seizure) was assessed as probably related to HBOT. One death (suspected suicide) was recorded in the placebo group, which was assessed as not related to the intervention. Most AEs were grade 1 (mild). In 19 subjects (49%), AEs were probably related to HBOT, and the most commonly reported AEs were cough and chest pain/discomfort, compared with 10 subjects (24%) on placebo. All AEs, except the death in the placebo group, were transient (table 5).

**Table 5:** Overview of AEs by treatment group (SAF)

	HBOT(n=39)	Placebo(n=41)
	n (%) AEs	n (%) AEs
AEs	19 (49%) 43	18 (44%) 38
Serious adverse events	1 (2.6%) 1	1 (2.4%) 1
Mild	32	26
Moderate	8	11
Severe	3	0
Deaths	0	1
Recovered/recovering	19 (100%) 43	17 (94%) 37
Fatal		1 (2.4%) 1
Relationship to IMP—possible	6 (15%) 11	8 (20%) 12
Relationship to IMP—probably	19 (49%) 21	10 (24%) 12
Action taken regarding IMP (discontinued)	6 (15%) 14	3 (7%) 5

Data isare presented as number of subjects (n), proportion of subjects (%) and number of (AEs) medicinal product.

AEs, adverse events; HBOT, hyperbaric oxygen treatment; IMP, investigational medicinal product; SAF, safety population.

Detailed lists of all AEs including common terminology criteria for AEs severity grade, MedDRA System Organ Classes and preferred term are found in supplemental material (online supplemental table 5A–C).

In the PPAS population, the proportion of subjects with a clinically relevant change of at least five units in the physical domains, the difference between the groups was statistically significant and greater (p=0.024) at 52 weeks (table 6), and in a post hoc analysis of the subjects that had received all 10 treatments (PPS10), the difference between groups was statistically significant also (p=0.012) at 52 weeks (online supplemental table 6).

**Table 6:** Proportion of subjects that received at least five treatments and had a clinically relevant change in PF and RP (PPAS)

Endpoint RAND-36	Week	Randomised to treatment				
		HBOT	Placebo	OR	P value*	Total
		n (%)	n (%)			n (%)
PF	13	18 (48.6%)	19 (50.0%)	0.95	0.907	37 (49.3%)
PF	26	20 (55.5%)	19 (57.1%)	1.05	0.914	39 (54.9%)
PF	52	24 (68.6%)	13 (40.1%)	**3.18**	**0.024**	37 (48.0%)
RP	13	6 (16.2%)	3 (7.9%)	2.26	0.277	9 (12.0%)
RP	26	5 (13.9%)	4 (11.4%)	1.25	0.756	9 (12.7%)
RP	52	9 (25.7%)	7 (21.9%)	1.24	0.713	16 (23.9%)

Proportion of subjects with at least ten10 units of improvement from baseline. ratio. * Chi-square test, Cochran-Mantel Haenszel test, adjusted for sex category and disease severity. At week 13: n=37; n=35, week 26: n=36; n=35, week 52: n=35; n=32 (HBOT; placebo).

Bold values indicate a statistically significant difference.

* χ2 test, Cochran-Mantel Haenszel test, adjusted for sex category and disease severity.

HBOT, hyperbaric oxygen treatment; PF, physical functioning; PPAS, per-protocol analysis set; RP, role limitations due to physical health.

Subgroup comparison in the primary endpoint reveals that there was a difference in favour of HBOT compared with placebo among female subjects, however, not statistically significant, in SAF, PPAS and PPS10 analysis populations; FAS: PF (n=32 HBOT, n=32 placebo): 2.038 (4.43) –6.86 to 10.91, p=0.648, RP: 7.019 (3.76) –0.509 to 14.55, p=0.067**,** PPAS: RP (n=28+32) with at least 5 treatments 7.980 (4.006) –0.038 to 15.999, p=0.051, PPS10: RP (n=24+29) with at least 10 treatments 9.059 (4.566) –0.11 to 18.23, p=0.053.

In addition, we observed a difference in favour of HBOT compared with placebo in the proportion of female subjects, who received all 10 treatments and had a clinically relevant increase of 10 units in RP, at 13 weeks compared with baseline. However, it was not statistically significant (p=0.135) (FAS) (online supplemental table 7). Among female subjects, there was also a difference in favour of HBOT compared with placebo in subjects who had a clinically relevant increase of 10 units in PF at 52 weeks, compared with baseline. However, this was not statistically significant (p=0.096) (FAS) (online supplemental table 7). For male subjects, there were no statistically significant differences between treatment groups, PF: −7.776 (6.47) –18.87 to 9.32, p=0.475, RP (n=8 HBOT, n=7 placebo): −17.53 (15.95) –49.34 to 14.26, p=0.253.

To validate the blinding procedure and sham treatment as a placebo control, all subjects were asked to guess what treatment they received and on unblinding, the answers were compared with the actual treatment received. The McNemar’s test detected no association between treatment groups and guess of treatment (p=0.728).

## Discussion

We could not detect a statistically significant difference between HBOT and sham treatment in any of the primary or main secondary endpoints in the overall subject population. We chose the physical domains in RAND-36 as the primary endpoint, but there was no statistically significant difference between treatment arms in any of the domains, in the short term. HBOT has become an increasingly popular ‘off-label’ treatment for long covid, and 10 sessions have been suggested to be effective in the short term.^25^ The lack of evidence for efficacy in our primary and secondary endpoints could be due to several factors.

First, the sample size may be too small. We based our power calculation on similar studies, since no other RCTs with HBOT for long covid were published when we started our trial. A new sample size calculation based on the trial’s results, with a mean difference of seven, and a common SD of 20 and an effect size of 0.350, implied that 130 patients are needed in each group to detect a significant difference between groups, using a t-test for independent groups with 80% power and significance level of 0.05 (5%). A previous RCT has shown that 40 sessions of HBOT at 2.0 ATA, 5 days a week, 90 min with air breaks every 20 min were effective short term for cognitive function and for secondary endpoints, improving HRQoL.^10^ A long-term follow-up at 52 weeks showed that the improvement in HRQoL was sustained at 1 year.^12^

Second, it is possible that a different dose than the one used in our protocol, with only 10 sessions in total and two to three times a week, is needed in a mixed population. It should also be noted that because of severe fatigue and PEM, most subjects in our cohort could not cope with treatments on consecutive days, which may also have impacted the outcome. It can be speculated that a more intense dose may give a better effect both short and long term. However, a gradual increase in intensity, or a lower dose (2.0 ATA) over an extended time, may benefit patients with severe PEM. A recent study has shed some light on the pathophysiology of PEM in patients with long covid. Induction of PEM was associated with local and systemic metabolic disturbances, severe exercise-induced myopathy and more tissue infiltration of amyloid-containing deposits in skeletal muscles of patients with long covid compared with healthy controls.^26^ We have previously shown similarities in gene expression in hypoxia and inflammatory pathways between high intensity interval training and HBOT in immune cells from healthy subjects.^17^ We decided on a minimum of five treatments for our per-protocol analysis, which was liberal and not sufficient to confirm efficacy. However, in an exploratory analysis of the subjects who received all 10 treatment sessions, there was a statistically significant result at 1-year follow-up, in the proportion of subjects who were detected with a clinically meaningful improvement of 10 points in the RAND-36 PF domain. This suggests that 10 treatments, two to three times a week, could be sufficient in selected cases. However, some researchers and clinicians argue that a more intense dose with 40–60 sessions is needed for a sustained effect on neuroplasticity.^27^ The optimal individual dose required for sustained efficacy remains to be determined, and further studies would benefit from a higher number of subjects, allowing for subgroups.

There was one subject with missing data for thearrythmias primary endpoint. The subject was on placebo treatment and deceased before the 13-week follow-up. Since this is only 1 out of the total 80 subjects for the analysis of the primary endpoint, it could have no substantial impact on the findings. Analysis was done for the observed cases in the population. There were no missing data for the covariates sex and disease severity at baseline.

In our subgroup analysis, we observed a clinically important sex difference, suggesting that 10 HBOT sessions seem more effective for women than for men. Although it did not reach statistically significant evidence. Since efficacy data were skewed towards a sex difference, the subgroup comparison below or above the mean RP/PF 30 at baseline was not meaningful. These observed sex differences may warrant further investigation in future studies.

The dosing protocols in current guidelines for HBOT differentiate for indication but not for sex category. We have previously shown that there is a sex difference in response to one short session of HBOT in healthy volunteers with a mean age of 47 years.^17^ In an older cohort, mean age of 65 years, with severe COVID-19, we could not detect the same sex difference with five sessions of HBOT.^16^ More research is needed to clarify if the sex difference is important in other indications for HBOT and in different age groups.

The minimal clinically important difference (MCID) for RAND-36 is not defined for long covid, but a composite score of three to five has been suggested.^28^ We decided to use 10 as a MCID for primary endpoints in our power calculation. Both groups had a clinically meaningful improvement during the course of the trial, which is most likely multifactorial. Many of our subjects had been desperately seeking help for their symptoms, and regular follow-up and support during the trial may have had a positive effect on their HRQoL. Due to the episodic nature of long covid, short-term PROMs such as the EQ-5D are difficult to interpret, and despite that RAND-36 spans over 4 weeks, other PROMs may be more adequate for long covid.^29 30^

The self-reported HRQoL was very low in our cohort compared with previously published data on long covid.^31^ Importantly, at baseline, we found that most subjects had a RAND-36 RP value of zero, which was not foreseen at the design stage of this trial, and had a negative impact on the previously calculated power. The very low result of the RP domain in RAND-36 can be interpreted as ‘expectations in relation to function’ and may be one of the best indicators of the subjects’ function prior to long covid. Our cohort comprised generally high-achieving subjects, both physically and intellectually, prior to long covid, which could explain a zero in the RP domain. Only a few subjects had a change in RP, suggesting that even though HBOT had a positive effect, subjects are far from normalised. Most of our subjects were unvaccinated and infected during the first wave of COVID-19 in 2020. Vaccination may reduce the risk of contracting long covid, but it is still unclear how it affects the recovery from the syndrome if vaccinated once symptomatic.^32^ There may be a selection bias depending on this fact and there has also been a selection of patients who have been diagnosed with long covid; only highly symptomatic cases were recruited.^3 33^ For the above reasons, our cohort may not be representative of the general post-COVID-19 condition population, but the problem with an increasing number of patients is a major socioeconomic burden that continues to grow.^34^

Previously published RCT reported no significant difference in side effects between the groups (35.1% and 38.9%, p = 0.739 in the HBOT and control groups, respectively) and no discontinuation of the treatment due to side effects.^10^ Most previous trials with HBOT have not reported AEs in compliance with ICH-GCP.^35^ The similarities in AE occurrence between groups suggest that the AEs are not related to oxygen itself. Given the frailty of this group, it’s possible that AEs occurred in the placebo group due to the effort of participation, by breathing non-humidified air in sham treatment or would be observed in those subjects even without treatment. There is also a possibility that the sham treatment is sufficient stress for this group of patients to have a physiological effect, which may explain the unexpectedly small difference between active treatment and sham treatment, and the similarities in AEs. Alternative explanations for the higher rate of AEs are difference in disease severity, difference in treatment protocols (2.0 ATA vs 2.4 ATA) or differences in reporting of AEs. Our trial was conducted in compliance with ICH-GCP, which includes a meticulous reporting of AEs.

### Conclusions

10 sessions of HBOT in a mixed population of long covid patients did not detect an improvement in self-reported physical function evaluated by RAND-36, or any of the main secondary outcomes in the short term. An exploratory analysis generated a hypothesis for a possible long-term effect. We discovered an important sex difference in the per-protocol population: 10 sessions may already be effective for women. HBOT delivered with medical grade oxygen and hospital standards of chamber safety seems safe for previously healthy patients with long covid. Further studies with subgroups for male and female populations and a dose of more than 10 sessions are required.

## Supplementary material

10.1136/bmjopen-2024-094386 online supplemental file 1

10.1136/bmjopen-2024-094386 online supplemental file 2

## Data Availability

Data are available upon reasonable request.
