# Supplementary material for: Ten sessions of hyperbaric oxygen versus sham treatment in patients with long covid (HOT-LoCO): a randomised, placebo-controlled, double-blind, phase II trial
Source: BMJ Open. 2025 Apr 14;15(4):e094386. doi: 10.1136/bmjopen-2024-094386 (PMC11997836; doi:10.1136/bmjopen-2024-094386)
Supplement: online supplemental file 1 [file bmjopen-15-4-s001.docx]

**Supplemental Figures and Tables**

| **Supplemental table 5a Adverse Events by Treatment Group, (SAF).** | | | |
| --- | --- | --- | --- |
|  | | **Actual Treatment Received** | |
|  |  | **Placebo** | **HBO2** |
|  |  | N=41 | N=39 |
| Number of AE per subject | 1 | 9 | 11 |
|  | 2 | 4 | 3 |
|  | 3 | 2 | 2 |
|  | 4 | 1 | 2 |
|  | 5 | 1 | 0 |
|  | 6 | 1 | 0 |
|  | 12 | 0 | 1 |
| Severity (CTCAE grade) | Grade 1 - Mild | 26 | 32 |
|  | Grade 2 - Moderate | 11 | 8 |
|  | Grade 3 - Severe | 0 | 3 |
|  | Grade 5 - Death | 1 | 0 |
| IS the AE serious? | No | 37 | 42 |
|  | Yes | 1 | 1 |
| Relationship to investigational product | Not applicable | 1 | 0 |
|  | Unlikely | 13 | 11 |
|  | Possible | 12 | 11 |
|  | Probable | 12 | 21 |

| **Supplemental table 5b Adverse Events by SOC, PT and Treatment Group, (SAF).** | | | | |
| --- | --- | --- | --- | --- |
| **Actual Treatment Received** | **Severity (CTCAE grade)** | **MedDRA SOC** | **MedDRA PT** | **Count** |
| **Placebo** | Grade 1 - Mild | Cardiac disorders | Atrial fibirillation | 1 |
|  |  | Ear and labyrinth disorders | Ear pain | 2 |
|  |  |  | Vertigo | 2 |
|  |  | Eye disorders | Subconjunctival haemorrhage | 1 |
|  |  |  | Visual impairment | 1 |
|  |  | General disorders and administration site conditions | Chest pain | 2 |
|  |  |  | Peripheral swelling | 1 |
|  |  | Infections and infestations | Sinusitis | 1 |
|  |  |  | Upper respiratory tract infection | 1 |
|  |  | Musculoskeletal and connective tissue disorders | Myalgia | 1 |
|  |  | Nervous system disorders | Headache | 3 |
|  |  | Repiratory, thoracic and mediastinal disorders | Respiratory tract infection | 4 |
|  |  |  | Cough | 6 |
|  | Grade 2 - Moderate | Cardiac disorders | Bradycardia | 1 |
|  |  |  | Palpitations | 1 |
|  |  |  | Tachycardia | 1 |
|  |  | General disorders and administration site conditions | Chest pain | 3 |
|  |  | Infections and infestations | Sinusitis | 1 |
|  |  |  | Upper respiratory tract infection | 1 |
|  |  | Injury, poisoning and procedural complications | Clavicle fracture | 1 |
|  |  | Musculoskeletal and connective tissue disorders | Back pain | 1 |
|  |  | Respiratory, thoracic and mediastinal disorders | Dyspnoea | 1 |
|  | Grade 5 - Death | Injury, poisoning and procedural complications | Traumatic haemorrhage | 1 |
| **HBOT** | Grade 1 - Mild | General disorders and administration site conditions | Chest discomfort | 1 |
|  |  | Cardiac disorders | Palpitations | 1 |
|  |  | Ear and labyrinth disorders | Ear discomfort | 1 |
|  |  |  | Ear pain | 5 |
|  |  |  | Hypoacusis | 2 |
|  |  | General disorders and administration site conditions | Chest discomfort | 1 |
|  |  | Musculoskeletal and connective tissue disorders | Pain in extremity | 2 |
|  |  | Nervous system disorders | Dizziness | 1 |
|  |  |  | Paraesthesia | 1 |
|  |  | Respiratory, thoracic and mediastinal disorders | Chest pain | 1 |
|  |  |  | Cough | 11 |
|  |  |  | Dyspnoea | 2 |
|  |  |  | Oropharyngeal pain | 1 |
|  |  | Skin and subcutaneous tissue disorder | Livedo reticularis | 1 |
|  |  |  | Paresthesia | 1 |
|  | Grade 2 - Moderate | Eye disorders | Visual impairment | 1 |
|  |  | General disorders and administration site conditions | Chest pain | 1 |
|  |  | Infections and infestations | COVID-19 | 2 |
|  |  | Musculoskeletal and connective tissue disorders | Neck pain | 1 |
|  |  | Nervous system disorders | Dizziness | 1 |
|  |  | Respiratory, thoracic and mediastinal disorders | Cough | 1 |
|  |  |  | Dyspnoea | 1 |
|  | Grade 3 - Severe | Nervous system disorders | Epilepsy with myoclonic-atonic seizures | 1 |
|  |  | Respiratory, thoracic and mediastinal disorders | Cough | 1 |
|  |  |  | Dyspnoea | 1 |

| **Supplemental table 5c Adverse Events by, Relationship to Study Medication, SOC, PT, and by Treatment (SAF).** | | | | | |
| --- | --- | --- | --- | --- | --- |
| **Actual Treatment Received** | **Relationship to investigational product** | **Severity (CTCAE grade)** | **MedDRA SOC** | **MedDRA PT** | **Count** |
| **Placebo** | Not applicable | Grade 1 - Mild | Ear and labyrinth disorders | Ear pain | 1 |
|  | Unlikely | Grade 1 - Mild | Cardiac disorders | Atrial fibirillation | 1 |
|  |  |  | Ear and labyrinth disorders | Ear pain | 1 |
|  |  |  | Infections and infestations | Sinusitis | 1 |
|  |  |  |  | Upper respiratory tract infection | 1 |
|  |  |  | Repiratory, thoracic and mediastinal disorders | Respiratory tract infection | 4 |
|  |  | Grade 2 - Moderate | Infections and infestations | Upper respiratory tract infection | 1 |
|  |  |  | Injury, poisoning and procedural complications | Clavicle fracture | 1 |
|  |  |  | Musculoskeletal and connective tissue disorders | Back pain | 1 |
|  |  |  | Respiratory, thoracic and mediastinal disorders | Dyspnoea | 1 |
|  |  | Grade 5 - Death | Injury, poisoning and procedural complications | Traumatic haemorrhage | 1 |
|  | Possible | Grade 1 - Mild | Ear and labyrinth disorders | Vertigo | 2 |
|  |  |  | Eye disorders | Subconjunctival haemorrhage | 1 |
|  |  |  | Musculoskeletal and connective tissue disorders | Myalgia | 1 |
|  |  |  | Nervous system disorders | Headache | 3 |
|  |  |  | Respiratory, thoracic and mediastinal disorders | Cough | 1 |
|  |  | Grade 2 - Moderate | Cardiac disorders | Bradycardia | 1 |
|  |  |  | General disorders and administration site conditions | Chest pain | 2 |
|  |  |  | Infections and infestations | Sinusitis | 1 |
|  | Probable | Grade 1 - Mild | Eye disorders | Visual impairment | 1 |
|  |  |  | General disorders and administration site conditions | Chest pain | 2 |
|  |  |  |  | Peripheral swelling | 1 |
|  |  |  | Respiratory, thoracic and mediastinal disorders | Cough | 5 |
|  |  | Grade 2 - Moderate | Cardiac disorders | Palpitations | 1 |
|  |  |  |  | Tachycardia | 1 |
|  |  |  | General disorders and administration site conditions | Chest pain | 1 |
| **HBOT** | Unlikely | Grade 1 - Mild | Ear and labyrinth disorders | Ear discomfort | 1 |
|  |  |  |  | Ear pain | 4 |
|  |  |  |  | Hypoacusis | 2 |
|  |  |  | Respiratory, thoracic and mediastinal disorders | Oropharyngeal pain | 1 |
|  |  | Grade 2 - Moderate | Infections and infestations | COVID-19 | 2 |
|  |  |  | Musculoskeletal and connective tissue disorders | Neck pain | 1 |
|  | Possible | Grade 1 - Mild | General disorders and administration site conditions | Chest discomfort | 1 |
|  |  |  | Cardiac disorders | Palpitations | 1 |
|  |  |  | Musculoskeletal and connective tissue disorders | Pain in extremity | 2 |
|  |  |  | Respiratory, thoracic and mediastinal disorders | Chest pain | 1 |
|  |  |  |  | Cough | 2 |
|  |  |  |  | Dyspnoea | 1 |
|  |  |  | Skin and subcutaneous tissue | Livedo reticularis | 1 |
|  |  | Grade 2 - Moderate | General disorders and administration site conditions | Chest pain | 1 |
|  |  | Grade 3 - Severe | Nervous system disorders | Epilepsy with myoclonic-atonic seizures | 1 |
|  | Probable | Grade 1 - Mild | Ear and labyrinth disorders | Ear pain | 1 |
|  |  |  | General disorders and administration site conditions | Chest discomfort | 1 |
|  |  |  | Nervous system disorders | Dizziness | 1 |
|  |  |  |  | Paraesthesia | 1 |
|  |  |  | Respiratory, thoracic and mediastinal disorders | Cough | 9 |
|  |  |  |  | Dyspnoea | 1 |
|  |  |  | Skin and subcutaneus tissue disorder | Paresthesia | 1 |
|  |  | Grade 2 - Moderate | Eye disorders | Visual impairment | 1 |
|  |  |  | Nervous system disorders | Dizziness | 1 |
|  |  |  | Respiratory, thoracic and mediastinal disorders | Cough | 1 |
|  |  |  |  | Dyspnoea | 1 |
|  |  | Grade 3 - Severe | Respiratory, thoracic and mediastinal disorders | Cough | 1 |
|  |  |  |  | Dyspnoea | 1 |

| **Supplemental table 6. Proportion of subjects that received all ten treatments and had a clinically relevant change in PF and RP (PPS10).** | | | | | | | | | |
| --- | --- | --- | --- | --- | --- | --- | --- | --- | --- |
| **Endpoint RAND-36** | **Week** | **Randomised to Treatment** | | | | | | | |
|  |  | **HBOT** | | **Placebo** | |  |  | **Total** | |
|  |  | n | % | n | % | OR | P-value* | n | % |
| PF | 13 | 14 | 48.3% | 15 | 48.4% | 1.03 | 0.949 | 29 | 48.8% |
| PF | 26 | 16 | 57.1% | 16 | 57.1% | 1.00 | 1.000 | 32 | 53.4% |
| PF | 52 | 20 | 71.4% | 9 | 36.0% | **4.52** | **0.012** | 29 | 54.3% |
| RP | 13 | 5 | 17.2% | 2 | 6.5% | 3.11 | 0.234 | 7 | 11.3% |
| RP | 26 | 3 | 10.7% | 4 | 14.3% | 0.67 | 0.651 | 7 | 12.3% |
| RP | 52 | 7 | 25.0% | 4 | 16.0% | 1.71 | 0.429 | 11 | 22.9% |
| Proportion of subjects with at least ten units of improvement from baseline in the PPAS population. OR=Odds ratio. * Chi-square test, Cochran-Mantel Haenszel test, adjusted for sex category and disease severity. At week 13: n=29; n=31, week 26: n=28; n=28, week 52: n=28; n=25 (HBOT; placebo). | | | | | | | | | |

| **Supplemental table 7. Proportion of female subjects that had a clinically relevant change in PF and RP (FAS)** | | | | | | | | | |
| --- | --- | --- | --- | --- | --- | --- | --- | --- | --- |
| **Endpoint RAND-36** | **Week** | **Randomised to Treatment** | | | | | | | |
|  |  | **HBOT** | | **Placebo** | |  |  | **Total** | |
|  |  | n | % | n | % | OR | P-value* | n | % |
| PF | 13 | 17 | 53.1% | 16 | 50.0% | 1.15 | 0.783 | 33 | 48.8% |
| PF | 26 | 16 | 53.3% | 17 | 58.6% | 0.81 | 0.680 | 33 | 53..4% |
| PF | 52 | 20 | 66.7% | 12 | 44.4% | 2.51 | 0.096 | 32 | 54.3% |
| RP | 13 | 5 | 15.6% | 1 | 3.1% | 7.35 | 0.135 | 6 | 11.3% |
| RP | 26 | 3 | 10.0% | 4 | 13.8% | 0.49 | 0.465 | 7 | 12.3% |
| RP | 52 | 6 | 20.0% | 5 | 18.5% | 1.10 | 0.891 | 11 | 22.9% |
| Proportion of female subjects with at least ten units of improvement from baseline in the FAS population. OR=Odds ratio. * Chi-square test, Cochran-Mantel Haenszel test, adjusted for sex category and disease severity. At week 13: n=32; n=32, week 26: n=30; n=29, week 52: n=30; n=27 (HBOT; placebo). | | | | | | | | | |

**Supplemental Figure 1.**


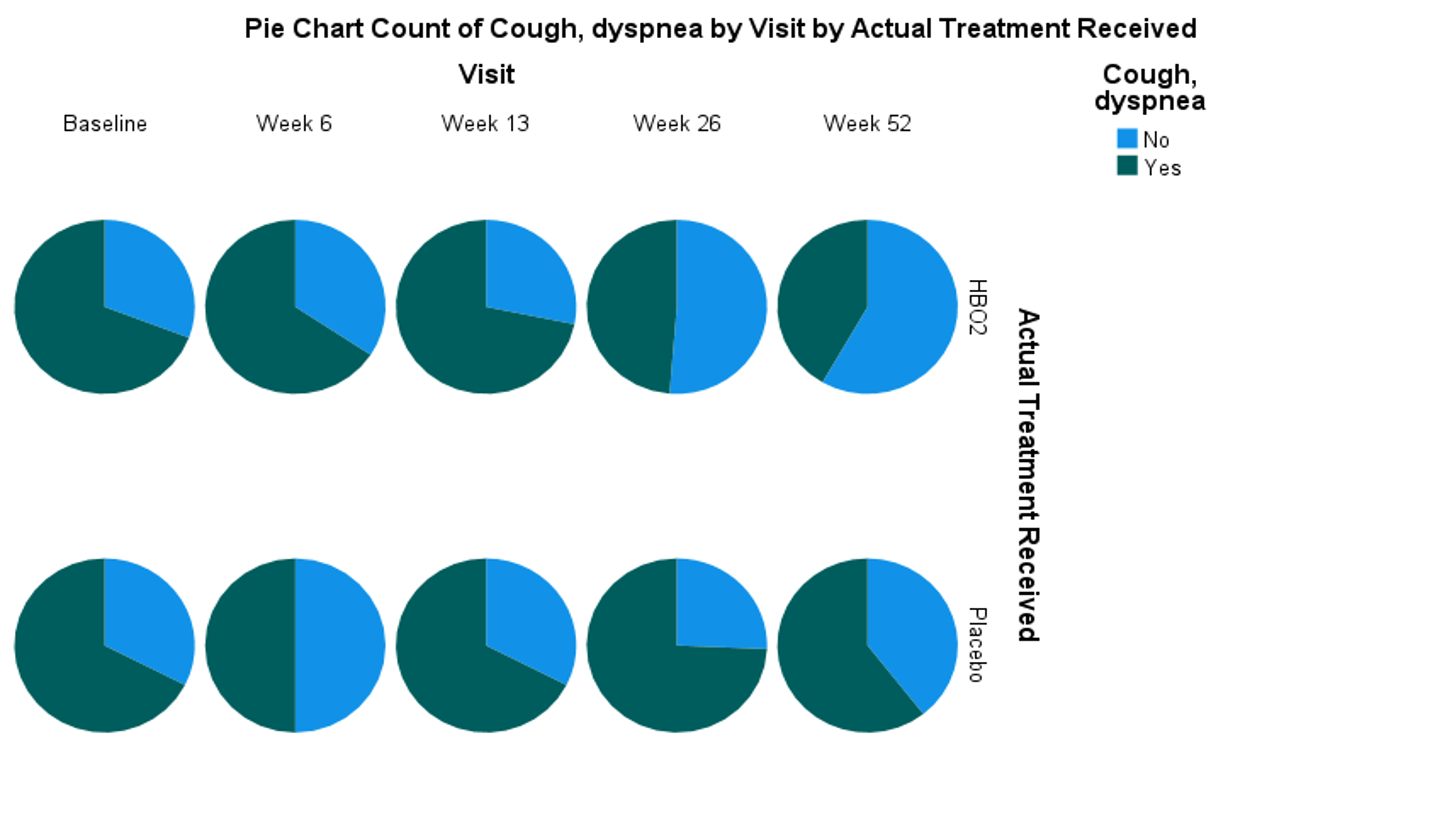


Supplemental Figure 1 Shows the propotion of reported symptoms cough/dyspnea by the subjects in each group. There is a graphical difference between the groups but only statistically significant (p=0.020), at 26 weeks.
