# Supplementary material for: Ten sessions of hyperbaric oxygen versus sham treatment in patients with long covid (HOT-LoCO): a randomised, placebo-controlled, double-blind, phase II trial
Source: BMJ Open. 2025 Apr 14;15(4):e094386. doi: 10.1136/bmjopen-2024-094386 (PMC11997836; doi:10.1136/bmjopen-2024-094386)
Supplement: online supplemental file 2 [file bmjopen-15-4-s002.pdf]

CLINICAL TRIAL PROTOCOL

Hyperbaric Oxygen for Treatment of Long COVID syndrome; A Randomized, Placebo-Controlled, Double-Blind, Phase II Clinical Trial

Safety and Efficacy of Hyperbaric Oxygen Therapy for Long COVID Syndrome

Trial code:	HOT-LOCO
EudraCT number:	2021-000764-30
ClinicalTrials.gov Identifier:	NCT04842448
Version number:	4
Date:	2022-01-03
Sponsor:	Karolinska University Hospital, Solna
Principal Investigator	Anders Kjellberg, MD

Table of Contents

Signature page 5
Contact information........ 6
List of used acronyms and abbreviations 7
1. Synopsis........ 9
2. Introduction........ 12
2.1 Background 12
2.2 Research hypothesis........ 13
2.3 Rationale for conducting the trial........ 14
3. Benefit-risk evaluation 14
3.1 The risk group 14
3.2 General risks with HBO_2 15
3.3 Blood sampling........ 15
3.4 Handling of sensitive personal data 15
3.5 Safety and logistics 16
4. Trial objectives and endpoints........ 16
4.1 Primary objective........ 16
4.2 Secondary objective(s)........ 16
4.2.1 Main secondary objective 16
4.2.2 Other secondary objectives 16
4.3 Primary endpoint: 17
4.4 Secondary endpoints: 17
4.4.1 Main Secondary Efficacy Endpoints 17
4.4.2 Other Efficacy Endpoints 17
4.4.3 Explorative/Descriptive Endpoints 17
4.4.4 Safety and Compliance Endpoints........ 18
5. Trial design and procedures........ 18
5.1 Overall Trial design and flowchart........ 18
5.2 Procedures........ 20
Trial schedule........ 21
5.2.1 Assessments and procedures........ 23
5.3 Biological sampling procedures 28
5.3.1 Handling, storage, and destruction of biological samples........ 28

5.3.2 Total volume of blood per subject ... 28
5.3.3 Biobank ... 28
5.3 End of Trial ... 29
6. Subject selection ... 29
6.1 Inclusion criteria: ... 29
6.2 Exclusion criteria: ... 29
6.3 Screening ... 30
6.4 Withdrawal Criteria ... 30
7. Trial treatments ... 31
7.1 Description of investigational product(s) ... 31
7.2 Dose and administration ... 31
7.3 Packaging, labeling, and handling of investigational products(s) ... 32
7.4 Drug accountability and treatment compliance ... 32
7.5 Randomization ... 32
7.6 Blinding ... 33
7.7 Code breaking ... 33
7.8 Concomitant Medication ... 33
7.9 Treatment after trial end ... 33
8. Handling of Adverse Events ... 34
8.1 Definitions ... 34
8.1.1 Adverse Event (AE) ... 34
8.1.2 Adverse Reaction (AR) ... 34
8.1.3 Serious Adverse Event (SAE) ... 34
8.1.4 Suspected Unexpected Serious Adverse Reaction (SUSAR) ... 34
8.2 Assessment of Adverse Events ... 35
8.2.1 Assessment of causal relationship ... 35
8.2.2 Assessment of intensity ... 35
8.2.3 Assessment of seriousness ... 36
8.3 Reporting and registration of Adverse Events ... 36
8.3.1 Reporting of Adverse Events (AE) ... 36
8.3.2 Reporting of Serious Adverse Events (SAE) ... 36
8.3.3 Reporting of Suspected Unexpected Serious Adverse Reactions (SUSAR) ... 37
8.4 Follow-up of Adverse Events ... 37

8.5 Safety Report (Development Safety Update Report, DSUR) 37
8.6 Procedures in case of emergencies, overdose or pregnancy 37
8.7 Reference Safety Information 38
9. Statistics 38
9.1 Statistical Analysis Plan 38
9.1.1 Analysis population 39
9.2 Statistical analyses 39
9.2.1 Sample size calculations 39
9.2.2 General statistical methodology 39
9.2.3 Patient Demographic and Baseline Characteristics 40
9.2.4 Primary Endpoint Analysis 41
9.2.5 Secondary Endpoints Analysis 41
9.2.6 Safety analyses 41
9.2.7 Interim Analysis 42
10. Quality Control and Quality Assurance 42
10.1 Quality Assurance and Sponsor oversight 42
10.2 Monitoring 42
10.3 Source data 43
10.4 Deviations or serious breaches 43
10.5 Audits and inspections 43
10.6 Data Safety Monitoring Board 44
10.7 Data protection 44
11. Ethics 45
11.1 Compliance to the protocol, GCP and regulations 45
11.2 Ethical review of the study 45
11.3 Procedure for obtaining informed consent 45
12. Insurances 46
13. Substantial changes to the trial 46
14. Collection, handling and archiving data 46
14.1 Case Report Form 46
15. Notification of trial completion, reporting, and publication 47
16. References 47
17. Amendments and Administrative changes 51

Signature page

Sponsor/Principal Investigator

I am responsible for ensuring that this protocol includes all essential information for the conduct of this trial. By signing my name below, I agree to conduct the trial in compliance with this protocol, the Declaration of Helsinki, ICH GCP (Good Clinical Practice) guidelines and the current hospital, national and international regulations governing the conduct of this clinical trial.

I will submit this protocol and all other important trial-related information to the staff members and investigators who participate in this trial, so that they can conduct the trial correctly. I am aware of my responsibility to continuously keep the staff members and investigators who work with this trial informed and trained.

I am aware that quality control of this trial will be performed in the form of monitoring, audit, and possibly inspection.

[signature] 2022-01-03

Sponsor/Principal Investigator's signature Date

Anders Kjellberg MD

Printed name

Contact information

**Role**	
Sponsor	Karolinska University Hospital<br>171 76 Stockholm
Principal Investigator,<br>Sponsor representative<br>Karolinska University Hospital	Anders Kjellberg, MD, PhD student,<br>ICU Consultant, Head of Hyperbaric unit,<br>Perioperative Medicine och Intensive Care<br>Karolinska University Hospital<br>Dept. Physiology and Pharmacology<br>Karolinska Institutet<br>171 76 Stockholm<br>+468760657355<br>anders.kjellberg@ki.se
Coordinating Investigator	Peter Lindholm, MD, PhD<br>peter.lindholm@ki.se<br>Dept. Physiology and Pharmacology<br>Karolinska Institutet
Co-investigators	Judith Bruchfeld, MD, PhD<br>Malin Nygren-Bonnier, Physiotherapist, PhD<br>Michael Runold, MD, PhD<br>Marcus Ståhlberg, MD, PhD<br>Kenny Rodriguez-Wallberg, MD, PhD<br>Sergiu Catrina, MD, PhD<br>John Pernow, MD, PhD
Trial site	Karolinska University Hospital, SE
Clinical monitoring organization,<br>Sweden	Karolinska Trial Alliance, KTA Support<br>Karolinska University Hospital Sabbatsbergs sjukhus<br>Olivecronas väg 15<br>113 61 Stockholm, Sweden
Senior Biostatistician	Jan Kowalski<br>JK Biostatistics AB<br>Karlbergsvägen 74<br>113 35 Stockholm, Sweden
Data Safety Monitoring Board	Kjell Ahlén, MD<br>Niklas Nielsen, MD, PhD<br>Stefan Grass, MD, PhD

List of used acronyms and abbreviations

Abbreviation	Term/Explanation
6 min walk test	6 minutes walk test (assessment of physical endurance)
30/60 s chair stand	30/60 seconds chair stand (assessment of functional mucscle strength)
AE	Adverse Event = any untoward medical occurrence
ANCOVA	Analysis of Covariance
AR	Adverse Reaction = adverse event, that is each unfavorable and unexpected reaction to a trial treatment, regardless of dose
ASA Class	ASA Physical Status Classification System
BP	Blood Pressure
CAT	COPD Assessement Test
COPD	Chronic Obstructive Pulmonary Disease
COVID-19	Corona Virus Disease 2019
CRA	Clinical Research Associate
CRO	Contract Research Organization
CT	Computerized Tomography
CXR	Chest X-Ray
DECT	Dual Energy Computed Tomography
DSMB	Data Safety Monitoring Board
DSUR	Development Safety Update Report = annual safety report
ECG	Electrocardiogram
eCRF	Electronic Case Report Form
EndoPAT	Endothelial assessement of Pulse Amplitude Tonometry
EPM	Etikprövningsmyndigheten (Swedish Ethical Review Authority)
EPR	Electron Paramagnetic Resonance Spectroscopy
EQ-5D	EuroQol 5 Dimensions questionnaire
FAS	Full Analys Set = including all data from all subjects who have participated in the trial
Frändin-Grimby	Frändin-Grimby activity scale
FSS	Fatigue Severity Scale
GAD-7	Generalised Anxiety Disorder 7-item scale
GCP	Good Clinical Practice
ICF	Informed Consent Form
ICH	International Council for Harmonization
IEC	Independent Ethics Comittee
IRB	Institutional Review Board

HBO_2	Hyperbaric Oxygen
HBOT	Hyperbaric Oxygen Therapy/Treatment
HIF	Hypoxia Inducible Factor
HRV	Heart Rate Variability (assessement for autonomic dysfunction)
HRQoL	Health-Related Quality of Life
HUT	Head Up Tilt test (assessment for POTS)
Jamar	Jamar (assessment of hand muscle strength)
kPa	kilo Pascal (SI unit for pressure, 100 kPa= 1 bar)
KSB	Kognitiva Screening Batteriet (Cognitive Screening Battery)
Long COVID	Long COVID Syndrome = PCS = PACS
LVFS	Läkemedelsverkets författningssamling (Swedish Medical Products Agency's statutes)
MIP/MEP	Maximal inspiratory and expiratory muscle strength
microRNA	Micro-Ribonucleic acid
MFS	Mental Fatigue Scale
mMRC	The Modified Medical Research Council Dyspnea Scale
MOCA	The Montreal Cognitive Assessement
MPA	Medical Products Agency
MRI	Magnetic Resonance Imaging
Nexfin	Nexfin noninvasive cardiovascular monitoring
PACS or PCS	Post (Acute) COVID-19 Syndrome = PCS = Long COVID
PBMC	Peripheral Blood Mononuclear Cells
PCL-5	Posttraumatic Stress Disorder Checklist (version 5)
PE	Pulmonary Embolism
PHQ-9	Patient Health Questionnaire-9
POTS	Postural Orthostatic Tachycardia Syndrome
PP	Per Protocol analysis = including only data from subjects who have completed the trial completely in accordance with the protocol, with no deviations from the protocol
RAND 36	RAND 36-Item Short Form Health Survey 1.0
RHI	Reactive Hyperemia Index
RNA	Ribonucleic acid
SAE	Serious Adverse Event = serious untoward medical occurrence
SAP	Statistical Analysis Plan
SPC or SmPC	Summary of (medical) Product Characteristics
SUSAR	Suspected Unexpected Serious Adverse Reaction
SARS-CoV-2	Severe Acute Respiratory Syndrome Corona Virus 2
SOP	Standard Operation Procedure
SpO_2	Peripheral Oxygen Saturation
TMF	Trial Master File
WAI	Work Ability Index (assessement of self reported work ability)

1. Synopsis

EudraCT number:	2021-000764-30
Title:	Hyperbaric Oxygen for Treatment of Long COVID Syndrome; A Randomized, Placebo-Controlled, Double-Blind, Phase II Clinical Trial
Trial code:	HOT-LOCO
ClinicalTrials.gov identifier:	NCT04842448
Short background/ Rationale/Aim:	Long COVID Syndrome (Long COVID), Post Acute COVID-19 Syndrome (PACS) or Post COVID-19 Syndrome (PCS) is defined as 'signs and symptoms that develop during or following an infection consistent with COVID-19, continue for more than 12 weeks and are not explained by an alternative diagnosis'. Common symptoms are fatigue, post-exertional malaise, brain fog, neurologic sensation, headaches, memory issues, insomnia, muscle aches, palpitations, shortness of breath, dizziness and speech issues. Many patients report very low Health Related Quality of Life (HRQoL) One in ten infected individuals may suffer persistent symptoms, and we are facing an emerging problem that will severely affect individuals, health care systems and society for years to come. Subjects will be recruited once they have been diagnosed with Long COVID through assessment by a multidisciplinary team with a thorough diagnostic work up including medical history, routine blood tests, questionnaires, physical tests and radiology.<br><br>We explore hyperbaric oxygen administered in a randomized placebo-controlled clinical trial as a potential treatment for patients suffering from Long COVID.<br><br>The overall hypothesis to be evaluated is that hyperbaric oxygen (HBO_2) treatment (HBOT) reduces oxidative stress and chronic inflammation, improves endothelial dysfunction and thereby alleviates symptoms associated with Long COVID.
Trial objectives:	**Primary objective:**<br><br>To evaluate if HBOT improves HRQoL (role limitations due to physical health and physical functioning for patients with Long COVID compared to placebo (sham treatment).<br><br>**Main secondary objectives:**<br><br>To evaluate if HBOT improves endothelial dysfunction in Long COVID.<br>To evaluate if HBOT improves objective physical performance in Long COVID.<br>To evaluate if HBOT improves HRQoL short term.

	To evaluate if HBOT can normalise physical functioning in Long COVID.<br><br>**Other secondary objectives (in selection):**<br><br>To evaluate if HBOT improves autonomic dysfunction.<br>To evaluate if HBOT improves restorative sleep.<br>To evaluate if HBOT has a long-term effect on subjective symptoms, HRQoL and objective physical performance in Long COVID<br>To evaluate the potential health-economic benefits of the treatment.
Trial design:	Randomized, placebo-controlled, double-blind, phase II
Trial population:	Previously healthy adult patients with Long COVID syndrome
Number of subjects:	80
Inclusion criteria:	1) Aged 18–60 years<br>2) Healthy or mild systemic disease (ASA 1-2) prior to COVID-19<br>3) Symptoms consistent with Long COVID for at least 12 weeks<br>4) Diagnosed with Long COVID, PACS, PCS (ICD-10 U09.9)<br>5) Working or studying prior to COVID-19<br>6) Documented informed consent according to ICH-GCP and national regulations
Exclusion criteria:	1) Known pregnancy or positive pregnancy test in women of childbearing age<br>2) ASA 3 or more from other cause than Long COVID<br>3) Score above 70 in RAND-36 Role Limitation Physical Health (RP) or Physical Functioning (PF)<br>4) Diabetes<br>5) Diagnosed with Hypertension prior to COVID-19<br>6) Contraindication for HBO_2 treatment according to local guidelines<br>7) Participation or recent participation in a clinical trial with an investigational product<br>8) Mental inability, reluctance or language difficulties that result in difficulty understanding the meaning of trialstudy participation
Investigational product(s), dosage, administration:	Hyperbaric oxygen (HBO_2) compared with placebo<br>HBO_2: HBO_2 240 kPa for 90 min, maximum 10 treatments

	Placebo: Air 134 kPa for 90 min, maximum 10 treatments
Trial endpoints:	**Primary endpoint:**<br>Mean change from baseline to 13 weeks in RAND 36 domains role limitations due to physical health and physical functioning.<br>**Secondary endpoints (in selection)**<br>Main Secondary Efficacy Endpoints:<br>I. Mean change from baseline to 13 weeks in Reactive Hyperemia Index (RHI).<br>II. Mean change from baseline to 13 weeks in the 6-min walk test.<br>III. Mean change from baseline to 13 weeks in the 30/60 sec chair stand.<br>IV. Mean change from baseline to 13 weeks in EQ-5D.<br>V. Proportion of subjects with a normalisation of levels in RAND-36 domains role limitations due to physical health and physical functioning respectively, at 13 weeks.<br>Safety and Compliance Endpoints<br>I. Number of subjects, proportion of subjects and number of AEs at 13 weeks.<br>II. Number of subjects, proportion of subjects that have completed planned treatments and number of treatments after 6 weeks.
Trial period:	Q3 2021 – Q4 2023
Statistical analyses	The analysis of the primary endpoint will be conducted on the Full Analys Set (FAS) and the Per Protocol Set (PPS).<br>The primary analysis of the primary endpoints will be performed using the ANCOVA, including randomisation strata of gender and disease severity together with treatment as fixed factors in the model.<br>The two primary endpoitns will be adequately adjusted for multiplicity.The p-value for testing the null hypotheses, no difference between treatment groups, must be less than 0.05 to be considered to have met the primary objective.

2. Introduction

2.1 Background

Post COVID-19 Syndrome (PCS), Post Acute COVID-19 Syndrome (PACS) or Long COVID Syndrome (Long COVID) has been defined as 'signs and symptoms that develop during or following an infection consistent with COVID-19, continue for more than 12 weeks and are not explained by an alternative diagnosis' (Venkatesan, 2021).

Common symptoms are fatigue, post-exertional malaise, brain fog, neurologic sensation, headaches, memory issues, insomnia, muscle aches palpitations, shortness of breath, dizziness and speech issues. Nearly 50% have reduced working capability and 22% cannot work at all. A majority are women and have never been hospitalized for acute COVID-19 (Davis et al., 2020).

The most common organ affected by the SARS-CoV-2 virus is the lung due to its main site of entry, binding to the angiotensin-converting enzyme 2 (ACE-2) receptor; resulting in damage to the cells of the alveolar-capillary membrane (Bourgonje et al., 2020). COVID-19 is associated with endothelial dysfunction, tissue edema and a pro-coagulant state in various organs including the lungs, liver, heart, kidney and small bowel (Varga et al., 2020).

Many of these changes may become chronic, which have been observed at post-COVID follow up (Halpin et al., 2021). COVID-19 should not only be viewd upon as an acute infection but as an inductor of a chronic inflammatory disease. Chronic oxidative stress, inflammation and endothelial dysfunction may explain many of the symtoms and objective findings associated with post-acute and long COVID even after recovery from the viral infection (Chang et al., 2020). These features are also hallmarks of other post-viral syndromes such as Myalgic Encepalomyelitis/Chronic Fatigue Syndrome (ME/CFS)(Scherbakov et al., 2020)

Endothelial dysfunction is a potentially reversible condition that serves as an independent predictor of cardiovascular events(Bonetti et al., 2003). Reactive Hyperemia Pulse Amplitude Tonometry (RH-PAT) is a non-invasive, user-independent tool for assessement of endothelial dysfunction. RH-PAT can be used in a clinical setting for monitoring of treatment effect in subjects with this condition (Bonetti et al., 2004). Autonomic dysfunction is an early marker of endothelial damage and is associated with cardivovascular morbidity and mortality (Khemani and Mehdirad, 2020). Endothelial dysfuncton is common in patients with ME/CFS and is associated with severity of symptoms and immune response (Scherbakov et al., 2020). Heart rate variability (HRV) is widely used as a standard method of measuring autonomic dysfunction in cardiovascular and neurological disorders (Rajendra Acharya et al., 2006).

Hyperbaric oxygen (HBO_2) (PO_2 240–280kPa) delivered by inhalation in a hyperbaric chamber in daily treatments over several weeks, has several anti-inflammatory effects. Hyperbaric

Oxygen Therapy/Treatment (HBOT) has been used safely for a century to treat other chronic inflammatory conditions such as radiation cystitis (Oscarsson et al., 2019), fibromyalgia (Efrati et al., 2015) and acute inflammatory conditions such as ulcerative colitis (Dulai et al., 2020). HBOT has shown beneficial effects on endothelial function in patients with slow coronary flow (Li et al., 2018). Usefully, it is possible to perform a placebo-controlled double-blind trial with HBO_2 (Lansdorp and van Hulst, 2018).

HBO_2 has been used off-label as one of few potentially curative treatments for acute COVID-19. Case series and a case control-study using HBO_2 have shown faster recovery and reduced need for ICU treatment (Guo et al., 2020, Thibodeaux et al., 2020, Gorenstein et al., 2020). RCTs are ongoing, including one at the Karolinska University Hospital (Clincaltrials.gov identifier: NCT04327505). Multiple hypotheses have been proposed for the effect of the therapy, with the common denominator being normalization of hypoxic- and inflammatory response (De Maio and Hightower, 2020, Kjellberg et al., 2020, Paganini et al., 2021).

One of the most studied effects of HBO_2 is attributed to Hypoxia Inducible Factor-1 (HIF-1) and target genes (Thom, 2011). One target for HIF-1 regulation, which is known to be associated with COVID-19, is Angiotensin Converting Enzyme 2 (ACE2). Hospitalized patients with COVID-19 have a three-fold higher expression of ACE2 in the lungs compared to healthy controls (Chua et al., 2020), suggesting a susceptibility for severe infection or an adaptive response. HIF-1 has been show to suppress ACE2, making HIF-1 modulation an interesting therapeutical target in COVID-19 (Serebrovska et al., 2020). Agents that stabilize HIF-1 have been proposed for COVID-19 (Afsar et al., 2020). A major challange in translating HIF-regulation into clinical practice is the complex adaptation to hypoxia and the intricate interplay between three different HIFs. The crosstalk between hypoxia and and inflammatory pathways adds further complexity to the system (D'Ignazio et al., 2016).

2.2 Research hypothesis

- HBO_2 can induce HIF signalling independent of heart, lung and brain function, thus has the potential to reduce inflammation, restore normal hypoxic response and thereby reduce morbidity in Long COVID.
- HBO_2 is safe and tolerable for Long COVID patients and the effect is accociated with relief in symptoms and thereby improve HRQoL .
- The effect can be monitored by markers of oxidative stress in blood and by non invasive assessement of endothelial dysfunction and autonomic dysfunction.
- Treatment results are not transient and thereby also cost efficient.
- The effect is related to regulation of hypoxia and inflammatory pathways.

2.3 Rationale for conducting the trial

Long COVID seems to affect approximately 10% of people infected with SARS-CoV-2, most of them are young women (Sivan and Taylor, 2020). To date, few treatment options are available. With over 100 million confirmed COVID-19 cases globally (600 000 in Sweden), the healthcare systems and their infrastructure are at risk of collapse if we cannot find an effective way of treating these patients. Karlolinska Universtity Hospitals was one of the first centers in the world to set up a multidisciplinary clinic for post covid sequaele and is now beening overwhelmed with referrals of suspected Long COVID.

The most common symtoms in Long COVID is chronic fatigue and autonomic dysregulation that are also hallmarks of Fibromyalgia (Sarzi-Puttini et al., 2020) and Myalgic Encepalomyelitis/Chronic Fatigue Syndrome (ME/CFS)(Lim et al., 2020) and some patients are also diagnosed with Postural Orthostatic Tachycardia Syndrome (POTS) and they may all be different semblance of the same chronic inflammatory disease. HBOT has been shown to have positive effect on ME/CFS and fibromyalgia in small clinical trials (Efrati et al., 2015, Yildiz et al., 2004, Akarsu et al., 2013).

If HBO_2 is effective for relieving symtoms in Long COVID there would be an obvious benefit for the individual patient. There is also a potential significant health-economic benefit if there is a lasting effect. The multiple explanatory endpoints may add valuble information about the disease for future interventional trials even with a negative result on the primary endpoint.

3. Benefit-risk evaluation

3.1 The risk group

There is currently no effective treatment available for Long COVID and since this is a new disease, there remain uncertainties regarding diagnosis, prognosis and mechanisms of action. There is emerging evidence that this may be an enormous problem for individuals, health-care and society. Diagnosis of Long COVID is mainly a clinical definition based on symptoms and it is difficult to find objective measurements. Patients that have been suffering from Long COVID since the first wave have often been misunderstood by the health care society and are desperate to find a cure for the disease. HBO_2 has the theoretical potential to reverse or reduce symptoms in Long COVID. The nature of the disease, which provokes multiple symptoms and a low quality of life make the risk group a 'vulnerable group' and it is important to make sure that the subjects are not unduly influenced by the expectation or benefits associated with participation. Therefore, we will conduct a placebo-controlled, double-blind, clinical trial in compliance with Good Clinical Practice (GCP), the Declaration of Helsinki and national regulatory requirements. The written information has a neutral language explaining both risks and potential benefits and the investigators are instructed to keep a neutral tone when delivering oral information. The cause of Long COVID is still not known and optimal

management is far from defined. We present a plausible hypothesis of the mechanism and a possible cure. Since there are no better options than 'expectation', and HBOT has been safely and effectively used in other chronic inflammatory conditions, the potential benefit for the subjects outweigh the risk.

3.2 General risks with HBO_2

HBOT is a well-established method that has been used for almost a century for several different indications. The mechanisms for its efficacy are not fully understood but it is generally regarded as safe, with few adverse events; serious adverse events are extremely rare. The Undersea and Hyperbaric Medical Society (UHMS) have reported a total of 40 complications per 10,000 treatments during 463,293 treatments over two years (Moon, 2019). Adverse events per 10,000 treatments include: ear pain 20, confinement anxiety 8, hypoglycaemic event 5, shortness of breath 2, oxygen-induced seizure 2, sinus pain, 1, chest pain. HBOT has very few contraindications that are all relative to the treatment environment; they include claustrophobia, medical history of spontaneous pneumothorax, severe COPD, and pregnancy.

3.3 Blood sampling

Blood sampling may have negative impact on the subject as a large number of samples will be necessary for the clinical investigation and may be needed for other trials. We aim to use blood tests already collected as much as possible. The blood sampling serves three purposes:

1. Safety, which is of benefit for the subject.

2. Explanatory, which may be beneficial for the placebo subjects in particular, if the trial results are positive and HBOT for Long COVID is adopted into clinical practice. Samples will serve as a quality control measure to ensure the validity of the data upon presentation of results.

3. Exploratory, which may benefit the subjects even if the HBOT is not successful, as the trial may generate hypotheses for alternative treatments.

Explanatory and Exploratory objectives are important for public health.

3.4 Handling of sensitive personal data

We will handle personal data, including gene expression analyses on the subjects, and there is a risk of personal integrity involved. The trial will be performed according to ICH-GCP; all staff involved will be educated in GCP. All information about the protocol and data will be entered into an eCRF. The data will not identify any person taking part in the trial in accordance with the EU Data Protection Directive (95/46/EU). An external monitor will help us assess the risks by assessing quality of trial design, data collection and informed consent.

3.5 Safety and logistics

The HBO_2 treatments will be performed in a hyperbaric chamber at the Karolinska University Hospital. Depending on availability, either monoplace or multiplace chambers will be used. The principal investigator is head of the unit with more than 20 years experience in HBOT. All staff are trained and certified for operating the chambers. Standard Operating Procedures for treatment will be used. Local, national and international guidelines for clinical trials and HBOT during the COVID-19 pandemic will be followed.

Monitoring will be conducted before, during and after the trial according to the monitoring plan. Interim analyses for safety and efficacy will be conducted by an independent Data Safety Monitoring Board (DSMB).

In summary, we believe the benefits for subjects, the risk-group and public health will outweigh the risks.

4. Trial objectives and endpoints

The overall hypothesis to be evaluated is that HBOT reduces oxidative stress and chronic inflammation, improves endothelial dysfunction and thereby alleviates symtoms associated with Long COVID.

4.1 Primary objective

To evaluate if HBOT improves HRQoL (role limitations due to physical health and physical functioning) for patients with Long COVID compared to placebo (sham treatment).

4.2 Secondary objective(s)

4.2.1 Main secondary objective

To evaluate if HBOT improves endothelial dysfunction in Long COVID.

To evaluate if HBOT improves objective physical performance in Long COVID.

To evaluate if HBOT improves HRQoL short term.

To evaluate if HBOT can normalise physical function in Long COVID

4.2.2 Other secondary objectives

To evaluate if HBOT improves autonomic dysfunction.

To evaluate if HBOT improves restorative sleep.

To evalute if HBOT has a long term effect on subjective symptoms, HRQoL and objective physical performance in Long COVID

To evaluate the potential health-economic benefits of the treatment.

To explore changes in general and organ-specific questionaires, physical tests and radiology used in clinical follow-up before and after treatment

To explore biomarkers in plasma, erythrocytes and PBMCs for HBO_2 effect on inflammation, endothelial function and chronic hypoxia.

4.3 Primary endpoint:

Mean change from baseline to 13 weeks in RAND 36 domains role limitations due to physical health (RP) and physical functioning (PF).

4.4 Secondary endpoints:

4.4.1 Main Secondary Efficacy Endpoints

I. Mean change from baseline to 13 weeks in Reactive Hyperemia Index (RHI).
II. Mean change from baseline to 13 weeks in the 6-min walk test.
III. Mean change from baseline to 13 weeks in the 30/60 sec chair stand.
IV. Mean change from baseline to 13 weeks in EQ-5D.
V. Proportion of subjects with a normalisation of levels in RAND-36 domains RP and PF respectively, at 13 weeks.

4.4.2 Other Efficacy Endpoints

I. Mean change in other RAND-36 domains at 13, 26 and 52 weeks compared to baseline.
II. Mean change in EQ-5D at 6, 26 and 52 weeks compared to baseline.
III. Mean change in physical activity using an activity meter at 6, 13 and 26 weeks compared to baseline
IV. Mean change in HRV using an activity meter at 6, 13 and 26 weeks compared to baseline
V. Mean change in sleeping pattern using an activity meter at 6, 13 and 26 weeks compared to baseline.

4.4.3 Explorative/Descriptive Endpoints

I. Mean change from baseline in hypoxia pathways in PBMCs evaluated by RNA sequencing, at 6, 13 and 26 weeks.
II. Mean change from baseline in inflammatory PBMCs evaluated by RNA sequencing, at 6, 13 and 26 weeks
III. Mean change from baseline of reactive oxygen species in red blood cells measured by EPR, at, 6 and 13 weeks.

IV. Mean change from baseline of microRNA in plasma, at 6 and 13 weeks.
V. Mean change from baseline in study specific clinical biochemistry at 6 and 13 weeks.
   a. D-Dimer
   b. Ferritin
   c. LDH
   d. Troponin T

VI. Mean change from baseline in objective organ specific findings on imaging at 13 and 26 weeks (from medical records).
VII. Mean change from baseline in objective organ specific and general physical tests (6-min walk test, 30/60-sec chair stand, HUT, Jamar, MIP/MEP and Spirometry at 13 and 26 weeks (from medical records).
VIII. Mean change from baseline in subjective rating of physical and cognitive symptoms evaluated by self-reported questionnaires (CAT, Frändin-Grimby, FSS, GAD-7, MFS, mMRC, MOCA, PCL-5, PHQ-9, WAI) at 13 and 26 weeks (from medical records).
IX. Long term post-trial follow-up of HRQoL using EQ-5D as variable up to 4 years post trial.

4.4.4 Safety and Compliance Endpoints

I. Number of subjects, proportion of subjects and number of AEs at 13 weeks.
II. Number of subjects, proportion of subjects that have completed planned treatments after 6 weeks.

5. Trial design and procedures

5.1 Overall Trial design and flowchart

Phase II Clinical Trial

Prospective randomized, placebo-controlled, double-blind, clinical trial,
estimated enrolment: 80 subjects

Parallel groups

Intervention: HBO_2: 240 kPa for 90 min, maximum 10 treatments within 6 weeks from randomization.

Control: Placebo treatment with 'sham' air breathing at a moderately higher pressure (134 kPa) for 90 min to simulate hyperbaric chamber treatment, maximum 10 treatments within 6 weeks from randomization.

The population will comprise of previously generally healthy patients diagnosed with Long COVID (U09.9). All patients are clinically assessed by a multidisciplinary team with a battery of questionnaires, physical tests, laboratory tests and radiology. After their first assessment,

individuals may have further organ specific work up for diagnosis, such as diagnosis of Postural Orthostatic Tachycardia Syndrome (POTS).

Once the patient has been diagnosed with Long COVID, they will be informed and asked to participate in the trial. No study specific procedures will take place before an informed consent form (ICF) has been signed. Some study specific procedures will be performed before inclusion (screening), such as HRQoL questionnaires and pregnancy test (if applicable). The patients will be included once they fulfil the inclusion criteria and exhibit none of the exclusion criteria. Baseline medial history, medical examination and study specific tests, blood samples and questionnaires will be collected during visit 1. If patients have already entered or gone through follow-up in clinical routine, some data from the last visit, no more than three months prior can be used for visit 1. If less than two weeks since last follow up, study specific procedures do not need to be repeated. Eligible subjects will be randomized within two weeks of the planned first treatment. Subjects will be randomized in a 1:1 alloction to HBO_2 or placebo (sham treatment). Scheduling of the HBOT will depend on available resources but the first treatment should be given within two weeks after randomization, and a maximum ten treatments should be given within 6 weeks from randomization. Physical tests, blood tests and questionnaires are repeated after the last treatment. Saftety and secondary endpoints are evaluated at visit 2. Efficacy evaluation of the primary endpoints will be made on assessments at visit 3 (three months), questionnaires and bloodtests. Subjects will be asked to use an activity meter in conjunction with each visit. Visit 4 and 5 are long term follow up, includes questionnaires, bloodtests and activity meter.

Clinical equipoise: The rationale for 1:1 randomization is that this is a new disease and that it will maximise the statistical power to detect a statistically significant efficacy between treatment groups.

Main efficacy and safety endpoints will be evaluated at one and three months after randomization, but all subjects will be asked to participate in a one-year follow-up after inclusion.

Subjects will also be asked to participate in a post-trial long-term follow-up with EQ-5D Questionnaire that will be sent out once a year for up to four years after visit 5.

Figure 1 and Table 1 show the trial overview and procedures

Figure 1. Trial flowchart

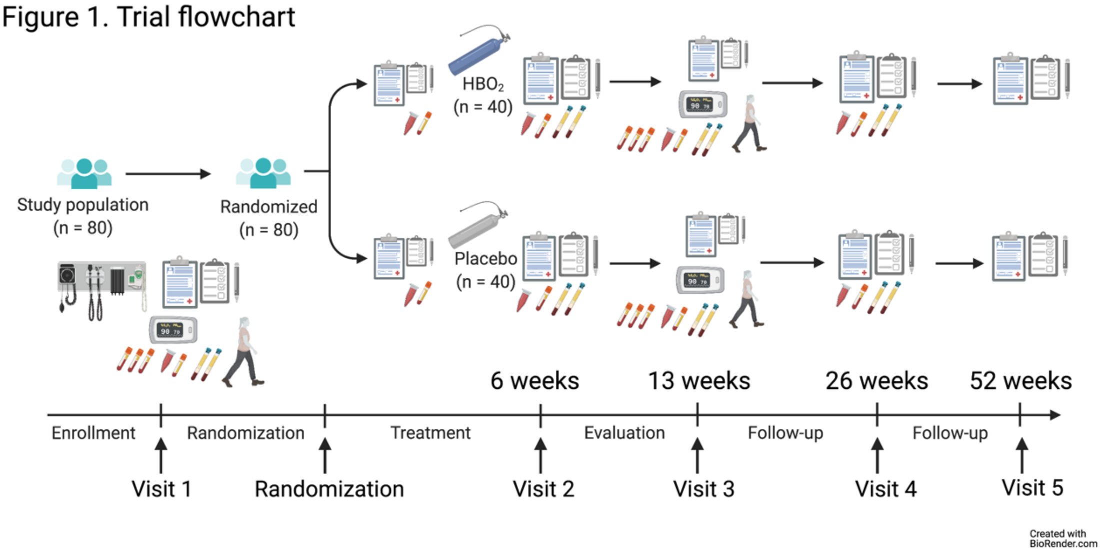

5.2 Procedures

Table 1. **List of procedures (Bold letters indicate study specific procedures**; other procedures may vary depending on symptoms and availability from medical records.)

Activity	Visit 1	Visit 2	Visit 3	Visit 4	Visit 5
Week	0	6	13	26	52
**Signed Informed consent Form**	**X**				
**Inclusion/exclusion criteria**	**X***				
**Randomization**	**X**				
**Medical history**	**X**	X**	X**	X**	X**
**Socio-demography**	**X**	X***	X***	X***	X***
**Concomitant medications**	**X**	**X**	**X**	X	X
**RAND 36**	**X**	X	**X**	**X**	**X**
**EQ-5D**	**X**	**X**	**X**	**X**	**X**
**RHI**	**X**		**X**		
**6 min walk test**	**X**	X	**X**	X	X
**30/60 s chair-stand**	**X**	X	**X**	X	
**Nexfin**	**X**		**X**		
**Treatment (HBOT/Placebo)**		**X (1-10)**			
**Treatment planned**		**X (1-10)**			
**AE/ADR**	**X**	**X**	X	X	X
**Study-specific biochemistry**	**X**	**X**	**X**	X	X
**Biobanking (PBMC, Plasma, EPR)**	**X**	**X, X**	**X**	**X**	
**Activity meter**	**X**	**X**	**X**	**X**	**X**

*Exclusion criteria includes a pregnancy test (if applicable), RAND 36 questionnaire, a HBOT specific questionnaire, review of medical records and a medical examination if needed.
**Medical history includes COVID-19 specific history, routine blood tests, questionnaires, physical tests, and radiology, medical records will be reviewed and recorded.
***Socio-demography that may change over time such as sick-leave, weight, activity, smoking habits.

Trial schedule

Visit 1: (Minimum 12 weeks post COVID-19)

a) After the patient has been informed about the trial and if agreed to participate, an **informed consent** form (ICF) will be **signed** off before any study-specific procedures occur.
During the **Screening**, procedures to assure the patient´s eligibility for trial participation will be performed, this includes a serum **pregnancy test** for females of childbearing potential, **RAND-36** and **EQ-5D questionnaires**, a **HBOT specific questionnaire**, review of medical records and a medical examination if needed for all. **Socio-demography**, **medical history** including COVID-19 specific history, adverse events, routine blood tests, questionnaires, physical tests, and radiology will be reviewed and recorded. **Questionnaires will be sent digitally and if eligible, subjects are booked for the physical tests.**

b) **Blood** samples for future biochemical research will be collected, and **study-specific chemistry** supplemented if necessary. **Study-specific procedures** will be conducted (not repeated if less than two weeks since last clinical visit and other relevant procedures will be recorded if less than 12 weeks since last clinical visit.

c) Subjects will be **randomized** to either HBO_2 or placebo when the first treatment is planned. Time, date and randomization group are recorded (blinded to subjects and all assessors of outcome variables).

Visit 2: (Starts within 4 weeks after visit 1, within 2 weeks of randomization, ends after last HBO_2 treatment)

Subjects are booked for the treatment.
a) **Review of medical records** and medical history. Advererse events, changes in concomitant medication, demographics, routine blood tests, questionnaires, physical tests and radiology will be reviewed and recorded.

b) **Blood** samples for future biochemical research may be collected before and after the first and the last treatment, **study-specific biochemistry** supplemented if necessary. Data from **activity meter** is registred. RAND 36 and EQ-5D questionnaires are sent digitally.

c) Subject will be introduced to the **Hyperbaric chamber** and given a **maximum 10 treatments within six weeks from randomization**. If planned but not given, this will be recorded with the reason for not giving the treatment.

Visit 3: (13 weeks after randomization +/- 2 weeks)

Questionnaires will be sent digitally and subjects are booked for physical tests.
a) **Review of medical records** and medical history. Adverse events, changes in concomitant medication, demographics, routine blood tests, questionnaires, physical tests and radiology will be reviewed and recorded.

b) **Blood** samples for future biochemical research will be collected, and **study-specific chemistry** supplemented if necessary.

c) **Study-specific procedures** will be conducted.

Visit 4: (26 weeks after randomization +/- 4 weeks)

Questionnaires wil be sent digitally to subjects.
a) **Review of medical records** and medical history. Changes in concomitant medication, demographics, routine blood tests, questionnaires, physical tests and radiology will be reviewed and recorded. Adverse events will be followed up.

b) **Study-specific blood** samples for future biochemical research will be collected, and **routine chemistry** supplemented if necessary. Data from **activity meter** is registred.

c) **Long term follow-up.**

Visit 5: (52 weeks after randomization +/- 4 weeks)

Questionnaires wil be sent digitally to subjects.
a) **Review of medical records** and medical history. Changes in concomitant medication, demographics, routine blood tests, questionnaires, physical tests and radiology will be reviewed and recorded. Adverse events will be followed up.

b) Data from **activity meter** is registred.

c) **Long-term follow-up.**

Unscheduled visits:

Any variables outside the timeframe of scheduled visits may be recorded as unscheduled visits during the trial.

End of Trial

A final visit in the electronic case report form (eCRF) should be completed for every randomised patient whether the patient completed the trial or not. The reason for any early discontinuation should be indicated on this form.

5.2.1 Assessments and procedures

Medical history

Relevant medical history will be recorded at Visit 1. The medical history will include a review of past and current relevant diseases/diagnoses/symptoms, for female subjects this includes information regarding menstrual cycle and pregnancies. Symptoms, signs and the start date of COVID-19, Long COVID and vaccination status will be collected. For concomitant diagnoses start year will be collected. Findings and/or abnormalities detected will be recorded in the eCRF. Other medical history, not relevant for the trial will be documented in medical records. Records and medical history will be reviewed for update/change in significantly changed parameters such as symptoms/signs or new diagnoses.

HBO_2 specific questionnaire

A HBO_2 specific questionnaire with focus on HBO_2 contraindications will be filled in by all subjects, contraindications include pregnancy, claustrophobia, obstructive lung disease and history of spontaneous pneumothorax. If anything in the questionnaire renders further examination, a review of medical records, an interview and a medical exam will be conducted. Findings and/or abnormalities detected will be documented in medical records with a statement “No contraindications for HBOT” or else the reason for contraindication.

Questionnaires

Change in RAND 36-item Health Survey (RAND-36), EQ-5D(euroquol.org) are used as primary and secondary enpoints , other questionaires may vary depending on clinical evaluation and main symptoms. Multiple questionnaries are used in clinical assessment including: RAND 36, EQ-5D, Frändin-Grimby activity sale, The Montreal Cognitive Assessment (MOCA), Work Ability Index (WAI), Mental Fatigue Scale (MFS), Fatigue Secerity Scale(FSS), Patient Health Questionnaire (PHQ-9), and the Generalized Anxiety Disorder (GAD-7), COPD Assessemnet Test(CAT), Medical Research Council(mMRC).

Medical records will be reviewed, time of questionnaire, reason for questionnaire and finding will be recorded. Baseline and change is categorized and described according to the reference in the specific questionnaire. SOPs for the study-specific questionnaires are available in the TMF, short description below:

RAND 36-item Health Survey 1.0 (RAND 36)

RAND 36 is a self-reporting questionnaire that contains 36 items that measure eight concepts of health in general terms, at present and past four weeks: physical functioning (10 items), role limitations due to physical health (4 items), role limitations due to emotional problems (3 items), energy/fatigue (4 items), emotional well-being (5 items), social functioning (2 items),

pain (2 items) and general health (5 items). It also includes a single item that provides an indication of perceived change in health over the last year. Scoring RAND 36 is a two-step process. First, numeric values from the survey are coded so that all items are scored from 0 (lowest score) to 100 (highest possible score). Scores then represent the percentage of total possible score achieved. In step 2, items in the same scale are averaged together to create the 8 scale scores. Items that are left blank (missing data) are not taken into account when calculating the scale scores. Hence, scale scores represent the average for all items in the scale that the respondent answered. RAND 36 is well documented in terms of reliability and variability also for Swedish translation (Orwelius et al., 2017).

EuroQol-5 Dimensions questionnaire (EQ-5D)

EuroQol-5 Dimensions questionnaire is a widely used self-reporting questionnaire that measure 5 dimensions of health TODAY at three or five levels (EQ-5D-3L or EQ-5D-5L) of severity; no problems, some/moderate problems and extreme problems/unable.The health dimensions are mobility, self-care, usual activities, pain/discomfort, anxiety/depression and a visual analogue scale (VAS) 0-100 which it used as a quantitative measure of overall health status. EQ-5D is the most widely used questionnaire for health-economy evaluation. Swedish population norm data for age and gender are available and can also be used for determining ability to work/study.

Physical tests

The 6 min walk test (American Thoracic Society), 30/60 sec chair stan (Jones et al 1997) , EndoPAT for measurement of RH-PAT and Nexfin (Edward Lifesiences) for measurement of cardiac indicies and activity meter for activity, heart rate variability (HRV) and sleep pattern are study-specific, other physical tests used in clinical practice may vary depending on main symptoms.
Multiple different physical tests are used in the clinical assessement including: 30/60-sec chair stand, Handgrip (Jamar), Spirometry, Maximal Inspiratory and Expiratory muscle strength (MIP/MEP), 6-min walk test, Head-Up-Tilt test (HUT).

Medical records will be reviewed, time of test, reason for test and finding will be recorded. Baseline and change is categorized and described according to the reference in the specific test. SOPs for the study-specific tests are available in the TMF, short description below:

6 minute walk test
The test is conducted in a corridor without obstacles with a measured distance of 30 meters (a cone is placed for start and turn) with markings every meters and double marknings every 5 meters. The subject carries a portable pulse/saturation meter.

- If the subject uses a walking aid the same should be used during the test, type of aid, if used is documented in the protocol.
- Periferal oxygen saturation (SpO_2) and pulse are recorded each minute.
- Any pauses during the test is noted, how long and posture during paus is recorded.

- A timer is started when the subject starts walking. The instructor only walks with the subject if deemed necessary from a safety perspective.
- Fatigue (general and leg) and dyspnea is self-rated with a Borg CR-10 scale immediately when the subject stops.
- The test is stopped it the subject experiences chest pain, SpO_2 below 80%, severe dyspnéa, cramping legs, staggering or wobbleing gait, perspiration or pale face. Time of disontinuation, cause and primary limiting factor is noted in the protocol.

30/60 sec chair stand

A red chair (44 cm high) is used, placed against a wall to minimise risk of falling.
The subject sits on the seat with a staight back, feet sholder wide with close to 90 degree angle in the knees, one foot slightly in front of the other. Arms crossed over chest.
The instructor demonstrates once and the subject practice once.

- The subject in instructed to stand up straight and sit down completely as many times possible during 60 seconds.
- A timer is started when the subjects back side lifts for the seat. The number of straight stands at 30 and 60 seconds is noted in the protocol. The subject is cheered on.The last stand is counted if the subject has risen more than half way at 60 seconds.
- Pulse, SpO_2, fatigue (general and leg) and dyspnea is self-rated with a Borg CR-10 scale immediately when the subject stops.

Nexfin
Subject is fasting, no beverage with caffein or sugar within 2 hours. The monitor is connected before 5 min rest in supine position without distraction. Non-invasive measurement of cardiovascular indicies with a beat-to-beat pulse wave analyzer placed on the middle phalang of one finger by Nexfin teqnology (ClearSight, Edwards Lifeciences). The ClearSight device comprices a pneumoatic pletysmograph that provides advanced hemodynamic parameters and continuous noninvasive blood pressure (BP) from a finger cuff with a redesigned self-coiling mechanism that reconstructs the clinical standard brachial arterial waveform from the finger arterial pressure waveform; it has been validated towards invasive measurements in a number of clinical trials.

- Measurement of beat-to-beat blood pressure and pulse including pulse-contour analysisis at rest and during physical tests.
- Registration of Heart rate, estimated Stroke volume, Cardiac index and Systemic vascular resistance index is recorded in the protocol.

EndoPAT
Subject is fasting, no beverage with caffein or sugar within 2 hours. The monitor is connected before 10 min rest in supine position without distraction. Non-invasive determination of digital endothelial function is measured with a pulse amplitude tonometry (PAT) device placed on the tip of each index finger (Endo-PAT2000; Itamar Medical, Caesarea, Israel). The PAT device comprises a pneumatic plethysmograph that allows measurement of pulse volume changes.

The PAT signal is recorded at baseline and following 5 min arterial occlusion using an inflatable blood pressure cuff placed on the forearm of one arm, while the contralateral arm serves as a control. The blood pressure cuff is inflated to 30 mmHg higher than the systolic pressure or a maximum of 200 mmHg for 5 min. The post-occlusive hyperemia stimulates endothelium-dependent vasodilatation causing an increase in digital pulse amplitude. The change from the baseline measurement is expressed as the reactive hyperemia index (RHI) which reflects vasodilator function of the digital microcirculation (Hamburg and Benjamin, 2009). Previous evaluation has demonstrated that reduced RHI reflects microvascular endothelial dysfunction, predicts cardiovascular events and reflects reduced NO bioavailability (Alexander et al., 2020).

Activity meter

The commercially available OURA™ ring will be used. The OURA™ ring is worn like a finger ring and has a number of sensors that register heart rate, temperature and physical activity. With the OURA™ ring it is possible to monitor HRV, level of physical activity, changes in body temperature and sleeping pattern. Subjects will be asked to wear the OURA™ ring at a minimum 1 week before and after each visit. Data will be automatically registred in a smartphone application and then uploaded to a secure encrypted database.

Radiology

Multiple different modalities of imaging are usesd in the clinical assessement including: Dual Energy Computed Tomography (DECT), Magnetic Resonance Imaging (MRI), Computed Tomography (CT) and normal chest X-ray (CXR). Review of records, document the time of radiology, reason for radiology and finding if it coincides with an interval of 12 weeks of visit 1, 3 4 and 5.

Socio-demography

Demographic data such as gender, age, level of education, rate of employment/studies, level/rate of exercise, country of origin, body weight, height, and smoking habits/ nicotine use will be collected at Visit 1. Records and medical history will be reviewed for update/change in parameters at each visit.

Concomitant and post-trial treatment(s)

Since Long COVID is a new syndrome, that may be chronic, without any definiete cure, “best practice” for symptomatic medications and other treatments are likely to change over the course of trial. Subjects are also likely to have tried or may try other remedies.

Medications and treatments that are considered “best practice” may be given to the subjects at the discretion of their attending physician/physiotherapist/phycologist. Subjects will be discouraged to try new medications, treatments or remedies that are not evidence based during the course of the trial.

Information regarding relevant regular concomitant medications, including vitamins, anti-oxidants, treatments and other remedies will be collected at Visit 1. Only relevant medications taken regularly, suspected to have caused an AE or used for treatment of an AE will be recorded. Changes in concomitant medications will be assessed (e.g. stop date or entry of a new treatment), throughout the trial by reviewing the patient's medical records and taking their medical history. Any changes will be recorded in the eCRF.

Blood samples

Routine biochemistry for kidney function, liver function, cardiac insult, haematology and blood glucose will be registered from the hospitals electronic system if they are outside normal range.

Study-specific blood tests that will be collected are: Ferritin, D-Dimer, LDH, Troponin T and a pregnancy test for women of childbearing age.

Date and time of collection and results from routine and study specific blood tests are recorded in the eCRF.

Details regarding the handling of blood sampling for laboratory analysis are found in section 5.3.

HBO_2 SOP and assessment

A standard operation procedure (SOP) will be attatched in the Trial Master File (TMF) but in general terms:

Subjects will be introduced to the hyperbaric unit; if required the subject may visit the unit before the first treatment. Treatment will be conducted in the multiplace (HAUX-STARMED-QUADRO 3500-2400) or monoplace chamber (SECHRIST 3300) depending on availability and number of subjets, at the discretion of the responsible physiscian. Subjects will be treated for 90 minutes; the treatment protocol is as follows - HBO_2 240 kPa with 10 min compression time and 10 minutes decompression time, and two air breakes, while placebo entails - 134kPa air, with 5 min compression time, and 5 min decompression to 120 kPa, and two air breakes will be reported to the subjects. Pressure gauges that can be seen by subjects will be covered. The frequency of the treatments and timing will depend on available resources at the discretion of the responsible physician but should be 2–5 treatments per week for 2–4 weeks. No treatment must be given more than 6 weeks after randomization.

Date and time for treatment will be recorded. Any planned treatment that could not be delivered and reason for the cancellation will be recorded. The treatment will be recorded on a separate CRF accessible only to staff designated to the treatment but blinded for the investigators performing assessements. Treatment type will be recorded in the eCRF and medical records once the code is broken or at the end of trial.

AE and ADR

Adverse events (AEs) and collection of AEs and Serious Adverse Events (SAEs) data.

Collection of AEs will start directly after inclusion and will be recorded until visit 3. Only SAEs will be collected outside the treatment period (visit 2). Ongoing AEs and SAEs at the end of visit 3 will be followed up during long-term follow-up until the subjects last visit. Definitions, documentation and reporting of AEs are described in detail in the AE section below.

5.3 Biological sampling procedures

5.3.1 Handling, storage, and destruction of biological samples

Study-specific routine biochemistry will be analysed at the Karolinska University Hospital laboratory (KUL).

Study-specific biobanking includes collection ot 4 extra tubes:

1x4ml EDTA plasma will be bio-banked for later analysis

2x8ml Citrate plasma (CPT-tubes) will be bio-banked for PBMC isolation and later analysis.

1x4ml heparin blood will be centrifuged and erythrocyte fraction will be incubated with CPH spin probe, bio-banked for later analysis of ROS in erythrocytes by EPR, plasma will be biobanked for later analysis.

CPT and EDTA tubes will be collected by a research nurse and transported immediately to the research laboratory Studiecenter Karolinska where PBMCs are isolated, half are prepared with RNA-later® for later RNA extraction and gene expression analysis and half is cryopreserved for later functional analysis of the monocytes. The monocytes, citrate-, EDTA- and heparin plasma will be stored in a sub-biobank at Bioclinicum Karolinska University Hospital. The biological samples will be saved until all analyses are performed.

5.3.2 Total volume of blood per subject

The study-specific blood will be maximum 40 ml (24 ml for all and additionally 16ml for some subjects). A maximum total amount of 200 ml blood is collected from each subject at five visits over nine months. This volume should be related to a blood donator that donates 450ml at one occasion that can be repeated every four months for women.

5.3.3 Biobank

Plasma, erythrocytes and PBMCs collected in this trial are registered in a regional biobank with an agreement with *Stockholms Medicinska Biobank (IVO reg nr 914)* and handled according to the current biobank laws and regulations. The samples are coded/psedonymized to protect the subject´s identification. All samples and the identification/code list are stored securely and separately to prevent unauthorized access.

5.3 End of Trial

The end of trial is defined as the last subject's final follow-up at visit 5 (week 52).

Premature termination of this clinical trial may occur because of a regulatory authority decision or at the discretion of the sponsor/the steering comittee.

The sponsor/steering comittee reserves the right to discontinue the trial at any time point in the following cases:
• Unexpected high proportion of AEs that are possibly or probably related to the trial drug.
• Trial protocol is difficult to cope with.
• Recruitment of eligible subjects is too low.

The end of the trial will be reported to the regulatory authority within 90 days, or 15 days if the trial is terminated prematurely. The Investigators will inform participants and ensure that the appropriate follow up is arranged for all involved.

6. Subject selection

6.1 Inclusion criteria:

To be included in the trial, subjects must meet the following criteria:

1) Aged 18–60 years
2) Healthy or mild systemic disease prior to COVID-19
3) Symptoms consistent with Long COVID for at least 12 weeks
4) Diagnosed with Long COVID, PACS, PCS (ICD-10 U09.9)
5) Working or studying prior to COVID-19
6) Documented informed consent according to ICH-GCP and national regulations

6.2 Exclusion criteria:

Subjects must not be included in the trial if any of the following criteria are met:

1) Known pregnancy or positive pregnancy test in women of childbearing age
2) ASA 3 or more from other cause than Long COVID
3) Score above 70 in RAND-36 domain Role Limitation Physical Health (RP) or Physical Functioning (PF)
4) Diabetes
5) Diagnosed with Hypertension prior to COVID-19
6) Contraindication for HBO_2 treatment according to local guidelines
7) Participation or recent participation in a clinical trial with an investigational product
8) Mental inability, reluctance or language difficulties that result in difficulty understanding the meaning of trial participation

6.3 Screening

Patients that have been assessed for Long COVID and that are likely to fullfil the inclusion criteria will be screened. Subjects will be informed about the trial by a study nurse during pre-screening and in detail about the trial by an investigator and after written informed consent, additional medical record review, HRQoL questionnaires, a HBOT specific questionnaire, physical examination (and pregnancy test if applicable) will be conducted. Subject eligibility (that subjects fulfill all inclusion criteria and do not meet any exclusion criteria) will be established before randomization to treatment.

6.4 Withdrawal Criteria

Subject participation: A subject will be considered to have completed the trial when he or she completes the assessment at 52 weeks (visit 5). Subjects should be encouraged to continue the trial but have the right to withdraw their consent or part of their consent regarding the trial participation e.g. to discontinue a study-specific blood test, but still participate in follow-up visits with questionnaires or not participate in further trial visits. The subject has no obligation to explain why he/she does not want to continue. The investigator also has the right to stop the subjects treatment in the event of AEs, protocol deviations, administrative reasons or any other reasons. It is understood by all concerned that an excessive rate of discontinues can render the trial uninterpretable. Therefore, unnecessary discontinuation should be avoided.

Irrespective of the reason for not continuing with the treatmentsl and whenever possible, the patient should be examined. Relevant laboratory test samples should be obtained and all relevant assessments should be completed if applicable. All AEs should be followed up until they have returned to baseline status or stabilised.
A termination visit (End of trial) in the electronic case report form (eCRF) should be completed for every randomised subject whether the subject completed the trial or not. The reason for any early discontinuation should be indicated on this form.

Subjects may be discontinued from the trial at the discretion of the Investigator. Specific reasons for discontinuing a subject from further assessments are:

AEs: Clinical or laboratory events that in the judgment of the investigator, DSMB or the Sponsor and in the best interest of the subject constitute grounds for discontinuation. This includes serious and non-serious AEs regardless of relation to trial drug.

Withdrawal of Consent: If a subject withdraws consent for disclosure of future information at the discontinuation of the trial or after completion of the trial, no further evaluations should be performed and no additional data should be collected. The Sponsor may retain and continue to use data collected before subject withdrew his/her consent. The Withdrawal of Consent reason is only applicable if the subject denies any further contact with site and no further data collection.

Lack of Efficacy/Treatment Failure: Subjects experiencing deterioration or no improvement of disease as judged by the investigator, may be discontinued from the trial at any time during the trial, offered alternative treatment and scored as treatment failures. Treatment failures includes significant disease worsening, requirement for surgical intervention and HBOT related SAE. Patients may be discontinued for sustained non-response at the discretion of investigator.

Protocol Violation: If the subject’s findings, or conduct, fails to meet protocol entry criteria or fails to adhere to the protocol requirements that make it impossible to derive sound scientific or medical conclusions from the primary endpoint data generated on a subject, (e.g. diagnose is changed after randomization or wrong treatment is given according to randomization).

Lost to Follow-Up: The subject does not show up for further visits and study personnel cannot reach the patient.

Other: Termination of other reason

If the subject discontinues the trial, follow-up of this subject will be performed according to the clinic’s routine but will be included in the Safety population if he/she have received at least one treatment.

7. Trial treatments

7.1 Description of investigational product(s)

Oxygen 100%, medical grade (Conoxia cryogen)

Placebo Air, compressed air medical grade

7.2 Dose and administration

Hyperbaric oxygen 240 kPa for 90 minutes (with 10 min compression time, two air breakes and 10 minutes decompression time). The number and frequency of treatments and timing will depend on the subject´s tolerance and available resources at the discretion of the attending physician, but the recommended interval is 2–5 treatments per week with a maximum of 10 treatments within 6 weeks from randomization. To satisfy the treatment compliance, a subject need to complete at least 5 treatments.

Placebo (134 kPa Air, with 5 min compression time, and 5 min decompression to 120 kPa, two air breakes will be reported to the subjects). The number and frequency of treatments and timing will depend on the subject´s tolerance and available resources at the discretion of the attending physician, but the recommended interval is 2–5 treatments per week with a maximum of 10 treatments within 6 weeks from randomization. To satisfy the treatment compliance, a subject need to complete at least 5 treatments.

7.3 Packaging, labeling, and handling of investigational products(s)

Treatment: 100% oxygen for medical use, cryogenic gas from hospital supply system. There will be no study-specific packaging or labeling.

Placebo: Compressed air from hospital supply system. There will be no study-specific packaging or labeling.

Treatments will be recorded in the eCRF, the code will be unblinded for staff administering the treatments but assessor-blinded. After the subjects end of study, the code will be broken and recorded in the medical records.

7.4 Drug accountability and treatment compliance

HBO_2 is delivered inside a hyperbaric chamber by inhaling 100% oxygen through a tight facemask (in selected cases a hood) attended by medical staff, or inside a monoplace chamber filled with oxygen. If the mask/hood is tight the inspired oxygen pressure is 233.7–240kPa (range depending on 100% saturated - dry gas) at 240 kPa pressure, hence there is no uncertainty about compliance. During compression/decompression patients may need to remove the mask in order to equalize the middle ears and the time might differ slightly between monoplace and multiplace chambers. The difference in dose during this period is neligleble. The date and time of treatment will be recorded in the eCRF. Compliance will be measured as the number and fraction of treatments planned vs given. Subjects that have been given at least 5 treatments will be analysed in the PP population. Any discrepancies from the protocol should be recorded in the eCRF.

7.5 Randomization

Subjects will be enrolled consecutively, as they are found to be eligible for inclusion in the trial, and randomized but after the treatment has been scheduled. Treatment should start within two weeks of randomization.

If a subject discontinues their trial participation, their subject code will not be reused, and the subject will not be allowed to re-enter the trial again. There will be no replacement for these subjects.

Eligible subjects will be randomized in a 1:1 allocation, stratified by disease severity in relation to RAND 36 and gender in blocks (blinded to all study personnel) to either HBO_2 or Placebo. There will be a computer generated randomization.

7.6 Blinding

This is a double-blind placebo-controlled trial where subjects and all study personell that participate in the asessement of sympoms and objective findings will be blinded to the treatment. The placebo protocol is well established and even experienced divers cannot differ between "sham treatment" and HBO_2 (Lansdorp and van Hulst, 2018). It is not unlikely that some sujects may have problems equalizing the ears even during placebo. Pressure gauges that can be seen by subjects will be covered and all staff will be informed not to discuss the treatment settings when subjects can hear and they will report two air brakes in the same manner as a normal treatment. To validate the blinding process all subjects will be asked at the end of the treatment if they believe they received placebo or HBO_2 and AE directly attributed to equalization problems will be compared.

7.7 Code breaking

The code is kept in the TMF in sealed envelopes, only accessed by staff designated to the hyperbaric unit if needed for safety reasons. If an AE or an SAE is reported, the PI should immediately assess the casual relationship and if an AR or SUSAR is suspected the code may be broken. Treatment type will be recorded in the medical records once the code is broken or at the end of trial.

7.8 Concomitant Medication

Medications that are considered necessary for the safety and well-being of the subject can be given at the discretion of the investigator, unless otherwise specified as an exclusion criterion.

All medications that the subject has taken regularly during the trial must be recorded in the eCRF. Non prescribed food supplements such as vitamins and anti-oxidants should also be recorded in the eCRF if taken regularly. Any changes need to be reported. Concomitant prescribed medications since start of symptoms shall be recorded at Visit 1.

7.9 Treatment after trial end

After an interval of six weeks no more HBO_2 must be given. The total dose during the trial will be recorded until six weeks after first treatment. At trial end, the participants will be treated according to routine clinical praxis.

8. Handling of Adverse Events

8.1 Definitions

8.1.1 Adverse Event (AE)

Adverse Events constitute any untoward medical occurrence in a clinical investigation subject administered a medicinal product and, which does not necessarily have a causal relationship with the treatment, can be an unfavorable and unintended sign (including an abnormal laboratory discovery), symptom or disease temporally associated with the use of the medicinal (investigational) product, whether or not related to the medicinal (investigational) product.

8.1.2 Adverse Reaction (AR)

In the new use of a medicinal product all noxious and unintended reactions to the medicinal product related to any dose should be considered an adverse reaction (AR). The phrase 'reaction to a medicinal product' means that the causal relationship between the medical product and an adverse event is at least a reasonable possibility, that is the relationship cannot be ruled out.

8.1.3 Serious Adverse Event (SAE)

Serious adverse events constitute any untoward medical occurrence that at any dose:

- results in death
- is life-threatening
- requires inpatient hospitalization or prolongation of existing hospitalization
- results in persistent or significant disability or incapacity
- results in a congenital anomaly/malformation
- regarded as medically important without meeting the above mentioned criteria

Medical and scientific assessment will be made to determine if an event is 'serious' and whether it would prompt reporting in other situations, for example important medical events that may not be directly life-threatening or result in death or hospitalization but may compromise the subject or may require intervention to prevent one of the other results set forth in the definitions above. These should also normally be considered as SAEs.

8.1.4 Suspected Unexpected Serious Adverse Reaction (SUSAR)

SUSAR comprise a reaction/event that is unexpected, serious, and suspected to be caused by the treatment, i.e. adverse events that are not included in the SPC.

8.2 Assessment of Adverse Events

8.2.1 Assessment of causal relationship

The investigator is responsible for determining whether there is a causal relationship between the AE/SAE and use of the investigational product.

Those AEs which are suspected of having a relationship to the investigational product will be followed up until the subject has recovered or is well taken care of and on their way to good recovery (see also section 8.4, Follow-up of Adverse Events).

All AEs will be categorized either as related, probably related, possibly related, unlikely related or not related, in accordance with the definitions below:

Related: Clinical event, including abnormal results from laboratory analyses, occurring in a plausible temporal sequence in relation to drug administration. The observed event matches with the known adverse reactions scheme for the drug involved. The event cannot be attributed to underlying disease or other medications.

Probably related: Clinical event, including abnormal results from laboratory analyses, occurring within a reasonable time after administration of the investigational product. The observed event match with the known adverse reactions scheme for the drug involved. It is unlikely attributable to underlying disease or other drugs.

Possibly related: Clinical event, including abnormal results from laboratory analyses, occurring within a reasonable time after administration of the intervention/investigational product. The event could be explained by the investigational product and its emergence is reasonable in relationship with use of the investigational product, but there is insufficient information to determine the relationship. The event could be explained by an underlying disease or other medications.

Unlikely related: Clinical event, including abnormal results from laboratory analyses, with a with a temporal relationship with respect to drug exposure that makes a relationship improbable (but not impossible). The event could be plausibly explained by an underlying disease or other medications.

Not related: Clinical event, including abnormal results from laboratory analyses that do not meet any of the above criteria for relatedness.

8.2.2 Assessment of intensity

Each adverse event shall be classified by an investigator as mild, moderate or severe.

Mild: Transient symptoms that are relatively tolerable and does not affect the subject's normal life.

Moderate: Marked symptoms, sufficiently unpleasant that interfere with the subject's normal life. Deterioration of function but is transient.

Severe: Unacceptable or incapacitating symptoms that causes deterioration of function to the extent that the subject is unable to perform normal activities.

8.2.3 Assessment of seriousness

The investigator is responsible for assessing the seriousness (serious or non-serious). If the incident is considered serious, this should be reported as a serious adverse event (SAE) by the investigator to the sponsor. See also section 8.3.2, Reporting of Serious Adverse Events (SAE).

8.3 Reporting and registration of Adverse Events

At each trial visit, AE are registered. Collection of AE data will start directly after inclusion and continue until 13 weeks (Visit 3) which is 7 weeks after the subject has ended their treatment with the investigational product. All AEs that occur during the trial and that are observed by the investigator/study-nurse or reported by the subject will be registered in the eCRF regardless of whether they are related to the investigational product or not. Assessment of causal relationship, severity, and whether the AE is considered to be an SAE or not will be done by the investigator directly in the eCRF. At minimum, for each AE/SAE, a description of the event is recorded (diagnosis/symptom if diagnosis is missing), start and stop times, causal relationship, severity, if the AE is considered to be an SAE or not, measures and outcome.

The following situations will not be reported as AE/SAE:

- Symptoms judged by the investigator as associated with Long COVID will not be recorded as an AE.
- A change in routine biochemistry will not be reported as AE unless detected during the treatment period.
- Non-serious adverse events outside the treatment period (visit 2) will not be recorded.

8.3.1 Reporting of Adverse Events (AE)

All AEs to be reported shall be registered in the eCRF continously.

8.3.2 Reporting of Serious Adverse Events (SAE)

Serious adverse events are reported to the sponsor on a special SAE form (included in the eCRF) within 24 hours of the investigator being informed of the SAE.

Follow-up information describing the outcome and handling of the SAE is reported as soon as this information is available.

The sponsor will in a timely manner assess whether the adverse event was expected for the investigational product or not, using the reference safety information. Serious AEs must be

collected, registered in the CRFs and an assessment of causality of the SAE should be performed. Also, discontinuations due to AEs will be collected.

8.3.3 Reporting of Suspected Unexpected Serious Adverse Reactions (SUSAR)

Those SAEs in Sweden which are assessed by the sponsor to be SUSARs are reported via a CIOMS form to the MPA that are submitting the CIOMS report to the to the European Medicines Agency (EudraVigilance database) according to the specified time frames.

SUSARs that are fatal or life-threatening are reported as soon as possible and no later than 7 days after the incident has become known to the sponsor. Relevant follow-up information is sent thereafter within an additional 8 days. Other SUSARs are reported as soon as possible and no later than 15 days after they have come to the sponsor's knowledge.

Any SUSAR will also be notified to the EPM by the sponsor.

Information about SUSARs occurring during the study is compiled by the sponsor and sent out to the principal investigator at all participating centers in connection to the event.

SUSARs in other participating countries will be reported to respective CA and EC according to applicable procedures

8.4 Follow-up of Adverse Events

All AEs should be followed up until they have returned to baseline status or stabilized until End of trial. AEs suspected to have a causal relationship with the trial intervention are followed until recovered or until the subject is on good way to recovery, follow-up will be done at the planned visits regadless of withdrawal from the trial.

8.5 Safety Report (Development Safety Update Report, DSUR)

During the trial period an annual Development and Safety Update Report (DSUR) will be submitted to the Swedish MPA and EPM .

The report includes a summary of all reported SAEs and SUSARs, a summarized safety assessment for trial subjects and information regarding potential updates of the risk-benefit assessment since trial approval.

8.6 Procedures in case of emergencies, overdose or pregnancy

The sponsor and investigator are obliged to immediately take the urgent safety measures necessary to protect the subjects from immediate danger. Examples of such measures are to temporarily suspend the clinical trial or to introduce supplementary monitoring measures.

The sponsor shall inform the MPA and EPM as soon as possible about the urgent safety measures taken by the investigator or sponsor.

If a subject who participates in a clinical trial for investigational products becomes pregnant, this person must be followed up until the birth has taken place. If the fetus/child has any congenital malformation, this must be reported as a serious adverse event or side effect (SAE).

8.7 Reference Safety Information

For reference safety information, reference is given in the SmPC.

9. Statistics

9.1 Statistical Analysis Plan

The principal features of the statistical analysis of the data are described in this section. A more technical and detailed elaboration of the principal features will be written in a separate Statistical Analysis Plan (SAP). The SAP will be finalised prior to Data Base Lock (DBL) and will include a more technical and detailed description of the statistical analyses described in this section. This section is a summary of the planned statistical analyses of the most important endpoints including primary and key secondary endpoints.

9.1.1 Analysis population

9.1.1.1 Definition of Trial Populations

9.1.1.1.1 The Full Analysis Set (FAS) Population; All randomized subjects who were exposed at least once to the study intervention will be included in the FAS population.

9.1.1.1.2 Per-Protocol (PP) Population; All randomized subjects with no major protocol violations will be included in the PP population. The final decisions regarding the PP population will be taken at the Clean File meeting before the database lock.

9.1.1.1.3 Safety Population; All randomized subjects that have received at least one treatment will be included in the safety population.

9.2 Statistical analyses

9.2.1 Sample size calculations

The assessment of the primary endpoints in this trial are based on the RAND 36-item health survey at baseline and 3 months where the domains of physical functioning and role functioning/physical defines the primary endpoints.

The primary endpoints have been used for long COVID (Garratt et al., 2021). There are norm data available for Sweden which enable us to determine a threshold for normalisation of individual RAND 36 domain levels. Using data from a few studies with similar methodology where RAND 36 has been previously used, we have assumed the standard deviation (SD) of Role Physical (RP), Physical Functioning (PF) 15.0. We expect the quality of life to be generally low in our cohort, especially in the RAND 36 RP and PF domains. We consider a ten points higher RAND 36 score in the HBO group compared to the placebo group to constitute a clinically relevant difference to be detected. Sample size calculation using t-test for independent groups, with 80% power, assuming a common SD of 15, and with a 5% significance level, reveals that at least 37 subjects per group are needed. We aim to recruit 80 subjects. An interim analysis will be made after 20 have undergone visit 2 to evaluate safety and when 40 subjects have undergone visit 3 in order to stop for futility and adjustment of sample-size if needed.

Sample size calculation was done in nQuery version 7.

9.2.2 General statistical methodology

Primary and secondary endpoints will be evaluated using the FAS population and sensitivity analyses performed using the PP population.

9.2.2.1 Statistical Hypotesis

The primary objective of the study is to confirm a superior efficacy for the active treatment compared to placebo in the primary endpoints. The null hypothesis to be tested is that there is no difference between HBO treatment and placebo, i.e., the mean change in (HBOT) = mean change (placebo). The same statistical hypothesis will be used for key secondary endpoints.

9.2.2.2 Adjustment for Multiplicity

The overall type I error rate for testing the primary efficacy endpoints will be controlled at the type I error rate of 0.05 using appropriate methods for adjustment of multiplicity in the primary. There will be no adjustment for multiplicity in main secondary endpoints but nominal p-values will be presented and results will be interpreted as exploratory findings.

All hypothesis tests will be two-sided. Details of the methods for adjustment in terms of the selection of endpoints to include in the testing sequence and the criteria for rejecting (or not rejecting) individual hypotheses are provided in the SAP.

9.2.2.3 Subgroups

The following subgroups will be evaluated for this study:

- Gender
- Disease severity
  - RAND-36 RP and PF below 30
  - RAND-36 RP and PF 30-50
  - RAND-36 RP and PF above 50

9.2.3 Patient Demographic and Baseline Characteristics

Baseline values and patient characteristics will be presented in tables by group and in total. All continuous variables will be described using standard statistical measures, i.e., number of observations, mean and median value, standard deviation, minimum and maximum value. All categorical variables will be summarised in frequency tables.

In general, continuous outcome variables will be analysed using ANCOVA, unless otherwise specified. Estimates will be presented using least square means for differences between treatment arms. For continuous endpoints that are measured repeatedly over time, such as EQ5D, RAND-36 domains, the change from baseline will be analyzed using a linear mixed effect model including baseline, treatment group, sex, symptom severity, visit, and treatment

group by visit interaction, and subjects as random effects, in the models. An unstructured covariance matrix will be assumed.

Analysis for categorical data in terms of binary data (Yes/No) will be presented as the proportion of participants with the frequency of presence or absence by treatment group of the characteristics of interest and analysed using the CMH Chi-square test, where the parameter used for the statistical hypothesis testing will be the OR, as a measure of the relative difference in odds between treatment arms. An OR>1 indicates an efficacy in favour of HBOT compared to placebo.

9.2.4 Primary Endpoint Analysis

The analysis of the primary endpoint will be conducted on the Full Analys Set (FAS) and the Per Protocol Set (PPS).

The primary analysis of the primary endpoints will be performed using the ANCOVA, including randomisation strata of main symptom and gender together with treatment as fixed factors in the model.

The two primary endpoitns will be adequately adjusted for multiplicity.The p-value for testing the null hypotheses, no difference between treatment groups, must be less than 0.05 to be considered to have met the primary objective.

9.2.5 Secondary Endpoints Analysis

The same analysis approach used for the primary efficacy endpoint will be applied to the secondary efficacy and exploratory endpoints as for the primary endpoints referred to as a ’Proportion endpoints’.

For categorical secondary endpoints, the CMH Chi-square test adjusting for disease severity and gender will be used to test for differences between treatments. Results will be presented using the frequency and the proportion by treatment group and the OR together with its corresponding 95% confidence interval.

All tests for the secondary endpoints will be two-sided on the 0.05 significance level. There will be no adjustment for multiplicity in main secondary endpoints.

All analysis will be done for the FAS population using observed data.

9.2.6 Safety analyses

Safety analyses will be performed on the Safety population.

9.2.6.1 Analysis of Adverse Events

The number and percentage of patients reporting AEs, and the number of AEs reported will be presented. The events will be tabulated by system organ class and preferred term. In addition, summaries by relationship to trial drug and severity will be presented. SAEs will also be presented in separate tabulations.

The number of patients experiencing an AE will be compared descriptively between groups. All patients with AEs will be listed individually with patient number in addition to type of event, start and stop time, duration, seriousness, severity, action taken, relationship to trial drug and outcome of AE.

9.2.7 Interim Analysis

Safety will be monitored continuously by the DSMB throughout the trial, an interim safety analysis will be performed when 20 subjects have available data for the safety endpoints.

There will be an interim analysis performed after 40 subjects have available data for the primary endpoint. The purpose of the interim analysis is to evaluate the assumption used for the sample size calculation and if necessary, to adjust the sample-size if needed. Also, the study will be evaluated for futility regarding the primary endpoints, to stop the study for futility (i.e., the predictive probability of success at the end of the study, given the data at the interim analysis) is less than 20%.

The DSMB will perform both interim analyses. A separate DSMB protocol will be created.

9.2.8 Handling of Dropouts and Missing Data

For the primary endpoint efficacy analyses, missing data will be adequately imputed for all subjects in the FAS population. In addition, the observed cases population will be evaluated as a sensitivity analysis. For secondary endpoints, only observed data will be analysed.

10. Quality Control and Quality Assurance

10.1 Quality Assurance and Sponsor oversight

The sponsor is responsible for having oversight of the trial´s quality. Steps to be taken to ensure the accuracy and reliability of data include the selection of qualified investigators and review of protocol procedures with the site personnel before the trial. eCRF completion guidelines will be provided and reviewed with study-personnel before the start of the trial.

10.2 Monitoring

The trial will be monitored by an independent monitor before the trial begins, during the trial conduct, and after the trial has been completed, so as to ensure that the trial is carried out

according to the protocol and that data is collected, documented, and reported according to ICH-GCP and applicable ethical and regulatory requirements. Monitoring is performed as per the trial's monitoring plan for which the sponsor is responsible and is intended to ensure that the subject's rights, safety, and well-being are met as well as data in the CRF are complete, correct, and consistent with the source data. The monitoring will be performed by an independent experienced monitor qualified in ICH GCP, applicable national and international regulations and the Declaration of Helsinki.

10.3 Source data

The investigator must keep source documents for each subject in the trial. Data in the eCRF can be source data, such as for certain demography parameters, AEs and assessment of SAEs. Source data is defined before trial start and a document describing what has been classified as source data in the trial should be included in the TMF. The investigator must ensure that all source documents are accessible for monitoring and other quality control activities.

10.4 Deviations or serious breaches

Serious breaches and deviations from the trial protocol, GCP and other regulations that significantly and directly affects, or with high likelihood could affect, the subjects or the scientific value of the trial, shall be reported within seven days (from knowledge) to the Swedish MPA. It is the sponsor's responsibility to judge the consequences of deviations that have occurred, and thus also to decide whether the Swedish MPA should be informed.

For major protocol deviations i.e violations see also section 6.4.

Minor deviations that do not affect subjects' integrity or safety, nor significantly affect the trial's scientific value, are documented in the trial documentation of the principal investigator and the sponsor.

10.5 Audits and inspections

Authorized representatives for the sponsor and Competent Authorities (CA) may carry out audits or inspections at the trial site, including source data verification. The investigator must ensure that all source documents are available for audits and inspections. The purpose of an audit or inspection is to systematically and independently review all study-related activities and documents, so as to determine whether these activities were performed, registered, analyzed and reported correctly according to protocol, Good Clinical Practice (GCP) and applicable regulations.

10.6 Data Safety Monitoring Board

An independent DSMB will evaluate the safety data in the context of the overall trial and the currently existing information about the trial drug. The DSMB will be composed of representatives from and experts in their respective disciplines of medicine, statistics and clinical trial methodology and conduct.

The DSMB will review the data during the course of the trial, as specified in table 2 below, and will draw up a charter delineating their guidelines for operating and stopping rules for terminating individual subjects, a portion or all of the trial prematurely. However, the DSMB may for any safety concerns recommend stopping of the trial even if these criteria are not fulfilled. It is the responsibility of the Sponsor to decide whether premature end of trial will be made, based on the advice provided by the DSMB

The DSMB will have access to all trial data. It may request and will be provided with whatever data is deemed necessary or useful for it to carry out its duties. The data provided will be blinded to treatment group unless specific unblinding is requested by the DSMB.

**Table 2. DSMB meeting schedule**	**Time of meeting**
Before trial start	Before first subject is included
Safety Interim analysis	When 20 subjects have completed visit 2
Interim analysis	When 40 subjects have completed visit 3
Efficacy analysis	When all 80 subjects have completed visit 3
End of the trial	Final visit has been done by the last subject.

10.7 Data protection

If any part of the data is handled by any other organization, inside or outside the EU, appropriate agreements and/or other documentation will be established, to ensure that the data processing is performed in accordance with the provisions of the General Data Protection Regulation (GDPR) and other relevant legislation, before any data transfer takes place.

The content of the informed consent form complies with relevant integrity and data protection legislation. In the subject information and the informed consent form, the subject will be given complete information about how collection, use and publication of their trial data will take place. The subject information and the informed consent form will explain how trial data are stored to maintain confidentiality in accordance with national data legislation. All information processed by the sponsor will be pseudonymized and identified with a Study-ID.

The informed consent form will also explain that for verification of the data, authorized representatives of the sponsor, as well as relevant authority, may require access to parts of

medical records or study records that are relevant to the trial, including the subject's medical history.

11. Ethics

11.1 Compliance to the protocol, GCP and regulations

The trial will be performed in compliance with the trial protocol, the Declaration of Helsinki, ICH-GCP (Good Clinical Practice) guidelines and current hospital, national and international regulations governing this clinical trial. This is to ensure the safety and integrity of the subjects as well as the quality of the data collected.

11.2 Ethical review of the study

The final trial protocol must be approved, as a part of the application for a permit for clinical trials, by both the Swedish Ethical Review Authority (Etikprövningsmyndigheten, EPM) and the Swedish Medical Products Agency (MPA) before the trial can be conducted. The final version of the informed consent form and other information provided to subjects, must be approved or given a written positive opinion by EPM. EPM and MPA must be informed of any changes in the trial protocol in accordance with current requirements.

11.3 Procedure for obtaining informed consent

The principal investigator shall ensure that the subject is given full and adequate oral and written information about the trial, its purpose, any risks and benefits as well as inclusion and exclusion criteria. Subjects must also be informed that they are free to discontinue their participation in the trial at any time without having to provide a reason. Subjects should be given the opportunity to ask questions and be allowed time to consider the provided information. If the person chooses to participate, both the subject and the investigator (qualified physician) shall sign the informed consent form. A copy of the subject information as well as the informed consent form shall be provided to the subject. The subject's signed and dated informed consent must be obtained before performing any study-specific activity in the trial. Each subject who participated in the trial will be identified by a subject number and if randomized, indentified by a randomization number on a subject identification list. The subject agrees that monitors, auditors, and inspectors may have access to their medical records and other source data. If new information is added to the trial, the subject has the right to reconsider whether he/she will continue their participation.

12. Insurances

Trial subjects are covered by the Swedish patient insurance and the Swedish pharmaceutical insurance.

13. Substantial changes to the trial

Substantial changes to the signed trial protocol are only possible through approved protocol amendments and by agreement from all responsible persons. Information on non-substantial changes should be clearly noted in the amended protocol.

In the event that substantial changes to the protocol (e.g., changing of the main objective, primary or secondary variables, method to measure the primary variable, changing of the investigational product or dosage) will be made during the course of the trial, approval from the MPA and EPM shall be obtained before any changes are implemented. A change that concerns a new site, new investigator or a new subject information sheet shall only be approved by the EPM, as applicable.

Non-substantial changes will be recorded and later entered in documentation that is submitted, for example in any subsequent notifications of a substantial change or in connection with End of Trial reporting.

14. Collection, handling and archiving data

Subjects who participate in the trial are coded with a specific trial identification number (Study-ID). All subjects are registered in a subject identification list (subject enrolment and identification list) that connects the subject's name and personal identity number with the Study-ID. When randomized a separate randomization number will be added.

All data will be registered, managed, and stored in a manner that enables correct reporting, interpretation, and verification. The complete Trial Master File, as well as source documents, will be archived for at least 10 years after the trial is completed. Source data in the medical records system is stored and archived in accordance with hospital regulations.

14.1 Case Report Form

An electronic Case Report Form (eCRF) is used for data collection. The investigator must ensure that data is registered and any corrections in the eCRF are made as stated in the trial protocol and in accordance with the instructions. The investigator must ensure that the registered data is correct, complete, and that reporting takes place according to the timelines that have been predefined and agreed. The investigator signs the completed eCRF. A copy of the completed eCRF will be archived at the trial site.

If an examination/test is not performed and data does not exist, ND (Not done) or NK (Not known) is marked. If the question is irrelevant NA (Not applicable) is written. Corrections in paper work sheets are done by striking out the incorrect information and adding the correct information next to the incorrect information, signing, and dating the correction.

15. Notification of trial completion, reporting, and publication

The MPA and EPM shall be informed of the trial's completion at latest 90 days after trial end, through submission of a "Declaration of End of Trial Notification" form.

Within one year after the trial is completed, the results shall be analyzed, a clinical trial report with individual data shall be prepared, and the trial results shall also be reported in the EudraCT database. The sponsor is responsible for the preparation of the clinical trial report. The statistical analyses will be performed and the results will be presented to the Investigator(s). Based on these data, the Principal investigator, in cooperation with the Co-Investigator(s), will prepare a clinical trial report. The report will be submitted to the competent authorities and will form the basis for a manuscript intended for publication in a medical/scientific journal. All personnel who have contributed significantly with the planning and performance of the trial may be included in the list of authors.

16. References

AFSAR, B., KANBAY, M. & AFSAR, R. E. 2020. Hypoxia inducible factor-1 protects against COVID-19: A hypothesis. *Med Hypotheses,* 143**,** 109857.

AKARSU, S., TEKIN, L., AY, H., CARLI, A. B., TOK, F., SIMSEK, K. & KIRALP, M. Z. 2013. The efficacy of hyperbaric oxygen therapy in the management of chronic fatigue syndrome. *Undersea Hyperb Med,* 40**,** 197-200.

ALEXANDER, Y., OSTO, E., SCHMIDT-TRUCKSASS, A., SHECHTER, M., TRIFUNOVIC, D., DUNCKER, D. J., ABOYANS, V., BACK, M., BADIMON, L., COSENTINO, F., DE CARLO, M., DOROBANTU, M., HARRISON, D. G., GUZIK, T. J., HOEFER, I., MORRIS, P. D., NORATA, G. D., SUADES, R., TADDEI, S., VILAHUR, G., WALTENBERGER, J., WEBER, C., WILKINSON, F., BOCHATON-PIALLAT, M. L. & EVANS, P. C. 2020. Endothelial Function in Cardiovascular Precision Medicine : A Position Paper on Behalf of the European Society of Cardiology. *Cardiovasc Res.*

BONETTI, P. O., LERMAN, L. O. & LERMAN, A. 2003. Endothelial dysfunction: a marker of atherosclerotic risk. *Arterioscler Thromb Vasc Biol,* 23**,** 168-75.

BONETTI, P. O., PUMPER, G. M., HIGANO, S. T., HOLMES, D. R., JR., KUVIN, J. T. & LERMAN, A. 2004. Noninvasive identification of patients with early coronary atherosclerosis by assessment of digital reactive hyperemia. *J Am Coll Cardiol,* 44**,** 2137-41.

BOURGONJE, A. R., ABDULLE, A. E., TIMENS, W., HILLEBRANDS, J. L., NAVIS, G. J., GORDIJN, S. J., BOLLING, M. C., DIJKSTRA, G., VOORS, A. A., OSTERHAUS, A. D., VAN DER VOORT, P. H., MULDER, D. J. & VAN GOOR, H. 2020. Angiotensin-converting enzyme 2 (ACE2), SARS-CoV-2 and the pathophysiology of coronavirus disease 2019 (COVID-19). *J Pathol,* 251**,** 228-248.

CHANG, R., MAMUN, A., DOMINIC, A. & LE, N. T. 2020. SARS-CoV-2 Mediated Endothelial Dysfunction: The Potential Role of Chronic Oxidative Stress. *Front Physiol,* 11**,** 605908.

CHUA, R. L., LUKASSEN, S., TRUMP, S., HENNIG, B. P., WENDISCH, D., POTT, F., DEBNATH, O., THURMANN, L., KURTH, F., VOLKER, M. T., KAZMIERSKI, J., TIMMERMANN, B., TWARDZIOK, S., SCHNEIDER, S., MACHLEIDT, F., MULLER-REDETZKY, H., MAIER, M., KRANNICH, A., SCHMIDT, S., BALZER, F., LIEBIG, J., LOSKE, J., SUTTORP, N., EILS, J., ISHAQUE, N., LIEBERT, U. G., VON KALLE, C., HOCKE, A., WITZENRATH, M., GOFFINET, C., DROSTEN, C., LAUDI, S., LEHMANN, I., CONRAD, C., SANDER, L. E. & EILS, R. 2020. COVID-19 severity correlates with airway epithelium-immune cell interactions identified by single-cell analysis. *Nat Biotechnol,* 38**,** 970-979.

D'IGNAZIO, L., BANDARRA, D. & ROCHA, S. 2016. NF-kappaB and HIF crosstalk in immune responses. *FEBS J,* 283**,** 413-24.

DAVIS, H. E., ASSAF, G. S., MCCORKELL, L., WEI, H., LOW, R. J., RE'EM, Y., REDFIELD, S., AUSTIN, J. P. & AKRAMI, A. 2020. Characterizing Long COVID in an International Cohort: 7 Months of Symptoms and Their Impact. *medRxiv***,** 2020.12.24.20248802.

DE MAIO, A. & HIGHTOWER, L. E. 2020. COVID-19, acute respiratory distress syndrome (ARDS), and hyperbaric oxygen therapy (HBOT): what is the link? *Cell Stress Chaperones***,** 1-4.

DULAI, P. S., RAFFALS, L. E., HUDESMAN, D., CHIOREAN, M., CROSS, R., AHMED, T., WINTER, M., CHANG, S., FUDMAN, D., SADLER, C., CHIU, E. L., ROSS, F. L., TOUPS, G., MURAD, M. H., SETHURAMAN, K., HOLM, J. R., GUILLIOD, R., LEVINE, B., BUCKEY, J. C., JR. & SIEGEL, C. A. 2020. A phase 2B randomised trial of hyperbaric oxygen therapy for ulcerative colitis patients hospitalised for moderate to severe flares. *Aliment Pharmacol Ther*.

EFRATI, S., GOLAN, H., BECHOR, Y., FARAN, Y., DAPHNA-TEKOAH, S., SEKLER, G., FISHLEV, G., ABLIN, J. N., BERGAN, J., VOLKOV, O., FRIEDMAN, M., BEN-JACOB, E. & BUSKILA, D. 2015. Hyperbaric oxygen therapy can diminish fibromyalgia syndrome--prospective clinical trial. *PLoS One,* 10**,** e0127012.

GARRATT, A. M., GHANIMA, W., EINVIK, G. & STAVEM, K. 2021. Quality of life after COVID-19 without hospitalisation: Good overall, but reduced in some dimensions. *J Infect*.

GORENSTEIN, S. A., CASTELLANO, M. L., SLONE, E. S., GILLETTE, B., LIU, H., ALSAMARRAIE, C., JACOBSON, A. M., WALL, S. P., ADHIKARI, S., SWARTZ, J. L., MCMULLEN, J. J. S., OSORIO, M., KOZIATEK, C. A. & LEE, D. C. 2020. Hyperbaric oxygen therapy for COVID-19 patients with respiratory distress: treated cases versus propensity-matched controls. *Undersea Hyperb Med,* 47**,** 405-413.

GUO, D., PAN, S., WANG, M. & GUO, Y. 2020. Hyperbaric oxygen therapy may be effective to improve hypoxemia in patients with severe COVID-2019 pneumonia: two case reports. *Undersea Hyperb Med,* 47**,** 181-187.

HALPIN, S. J., MCIVOR, C., WHYATT, G., ADAMS, A., HARVEY, O., MCLEAN, L., WALSHAW, C., KEMP, S., CORRADO, J., SINGH, R., COLLINS, T., O'CONNOR, R.

J. & SIVAN, M. 2021. Postdischarge symptoms and rehabilitation needs in survivors of COVID-19 infection: A cross-sectional evaluation. *J Med Virol,* 93**,** 1013-1022.

HAMBURG, N. M. & BENJAMIN, E. J. 2009. Assessment of endothelial function using digital pulse amplitude tonometry. *Trends Cardiovasc Med,* 19**,** 6-11.

KHEMANI, P. & MEHDIRAD, A. A. 2020. Cardiovascular Disorders Mediated by Autonomic Nervous System Dysfunction. *Cardiol Rev,* 28**,** 65-72.

KJELLBERG, A., DE MAIO, A. & LINDHOLM, P. 2020. Can hyperbaric oxygen safely serve as an anti-inflammatory treatment for COVID-19? *Medical Hypotheses,* 144.

LANSDORP, C. A. & VAN HULST, R. A. 2018. Double-blind trials in hyperbaric medicine: A narrative review on past experiences and considerations in designing sham hyperbaric treatment. *Clin Trials,* 15**,** 462-476.

LI, Y., ZHANG, H., LIANG, Y., WANG, W., XU, T., ZHANG, J., XIAO, W. & WANG, T. 2018. Effects of hyperbaric oxygen on vascular endothelial function in patients with slow coronary flow. *Cardiol J,* 25**,** 106-112.

LIM, E. J., AHN, Y. C., JANG, E. S., LEE, S. W., LEE, S. H. & SON, C. G. 2020. Systematic review and meta-analysis of the prevalence of chronic fatigue syndrome/myalgic encephalomyelitis (CFS/ME). *J Transl Med,* 18**,** 100.

MOON, R. E. (ed.) 2019. *Hyperbaric Oxygen Therapy Indications*: Undersea and Hyperbaric Medical Society.

ORWELIUS, L., NILSSON, M., NILSSON, E., WENEMARK, M., WALFRIDSSON, U., LUNDSTROM, M., TAFT, C., PALASZEWSKI, B. & KRISTENSON, M. 2017. The Swedish RAND-36 Health Survey - reliability and responsiveness assessed in patient populations using Svensson's method for paired ordinal data. *J Patient Rep Outcomes,* 2**,** 4.

OSCARSSON, N., MULLER, B., ROSEN, A., LODDING, P., MOLNE, J., GIGLIO, D., HJELLE, K. M., VAAGBO, G., HYLDEGAARD, O., VANGEDAL, M., SALLING, L., KJELLBERG, A., LIND, F., ETTALA, O., AROLA, O. & SEEMAN-LODDING, H. 2019. Radiation-induced cystitis treated with hyperbaric oxygen therapy (RICH-ART): a randomised, controlled, phase 2-3 trial. *Lancet Oncol,* 20**,** 1602-1614.

PAGANINI, M., BOSCO, G., PEROZZO, F. A. G., KOHLSCHEEN, E., SONDA, R., BASSETTO, F., GARETTO, G., CAMPORESI, E. M. & THOM, S. R. 2021. The Role of Hyperbaric Oxygen Treatment for COVID-19: A Review. *Adv Exp Med Biol,* 1289**,** 27-35.

RAJENDRA ACHARYA, U., PAUL JOSEPH, K., KANNATHAL, N., LIM, C. M. & SURI, J. S. 2006. Heart rate variability: a review. *Med Biol Eng Comput,* 44**,** 1031-51.

SARZI-PUTTINI, P., GIORGI, V., MAROTTO, D. & ATZENI, F. 2020. Fibromyalgia: an update on clinical characteristics, aetiopathogenesis and treatment. *Nat Rev Rheumatol,* 16**,** 645-660.

SCHERBAKOV, N., SZKLARSKI, M., HARTWIG, J., SOTZNY, F., LORENZ, S., MEYER, A., GRABOWSKI, P., DOEHNER, W. & SCHEIBENBOGEN, C. 2020. Peripheral endothelial dysfunction in myalgic encephalomyelitis/chronic fatigue syndrome. *ESC Heart Fail,* 7**,** 1064-1071.

SEREBROVSKA, Z. O., CHONG, E. Y., SEREBROVSKA, T. V., TUMANOVSKA, L. V. & XI, L. 2020. Hypoxia, HIF-1alpha, and COVID-19: from pathogenic factors to potential therapeutic targets. *Acta Pharmacol Sin,* 41**,** 1539-1546.

SIVAN, M. & TAYLOR, S. 2020. NICE guideline on long covid. *BMJ,* 371**,** m4938.

THIBODEAUX, K., SPEYRER, M., RAZA, A., YAAKOV, R. & SERENA, T. E. 2020. Hyperbaric oxygen therapy in preventing mechanical ventilation in COVID-19 patients: a retrospective case series. *J Wound Care,* 29**,** S4-S8.

THOM, S. R. 2011. Hyperbaric oxygen: its mechanisms and efficacy. *Plast Reconstr Surg,* 127 Suppl 1**,** 131S-141S.

VARGA, Z., FLAMMER, A. J., STEIGER, P., HABERECKER, M., ANDERMATT, R., ZINKERNAGEL, A. S., MEHRA, M. R., SCHUEPBACH, R. A., RUSCHITZKA, F. & MOCH, H. 2020. Endothelial cell infection and endotheliitis in COVID-19. *Lancet,* 395**,** 1417-1418.

VENKATESAN, P. 2021. NICE guideline on long COVID. *Lancet Respir Med*.

YILDIZ, S., KIRALP, M. Z., AKIN, A., KESKIN, I., AY, H., DURSUN, H. & CIMSIT, M. 2004. A new treatment modality for fibromyalgia syndrome: hyperbaric oxygen therapy. *J Int Med Res,* 32**,** 263-7.

17. Amendments and Administrative changes

The following amendments and Administrative changes have been made to this protocol since day of preparation:

Amendment	Section/Page	Date	Type/comment
Version 1		2021-05-05	EPM submission
Version 2<br>addition of sponsor representative signature<br>addition of DSMB members<br>addition of COVID-19 pandemic statement<br>minor layout and typos	Signature page/5<br><br>Contact information/6<br>3.5/16<br><br>Full protocol	2021-06-30	MPA submission/non substantial change
Version 3<br>specification of activity meter used<br>minor layout and typos	 5.2/21-26<br>Full protocol	2021-08-16	EPM amendment/non substantial change
Version 4<br>minor layout and typos<br>Change of treatment interval	 Full protocol<br>7.9	2022-01-03	Non substantial change<br><br>Incoherent with section 5
