# Supplementary material for: Ten sessions of hyperbaric oxygen versus sham treatment in patients with long covid (HOT-LoCO): a randomised, placebo-controlled, double-blind, phase II trial
Source: BMJ Open. 2025 Apr 14;15(4):e094386. doi: 10.1136/bmjopen-2024-094386 (PMC11997836; doi:10.1136/bmjopen-2024-094386)
Supplement: Supplementary file 3 [file bmjopen-2024-094386.reviewer_comments.pdf]

PEER REVIEW HISTORY

BMJ Open publishes all reviews undertaken for accepted manuscripts. Reviewers are asked to complete a checklist review form and are provided with free text boxes to elaborate on their assessment. These free text comments are reproduced below.

ARTICLE DETAILS

Title (Provisional)

Ten sessions of Hyperbaric Oxygen versus sham treatment in patients with Long COVID (HOT-LoCO): a randomised, placebo controlled, double-blind, phase II trial.

Authors

Kjellberg, Anders; Hassler, Adrian; Boström , Emil; El Gharbi, Sara; Al-Ezerjawi, Sarah; Schening, Anna; Fischer, Katarina; Kowalski, Jan H; Rodriguez-Wallberg, Kenny A; Bruchfeld, Judith; Ståhlberg, Marcus; Nygren-Bonnier, Malin; Runold, Michael; Lindholm, Peter

---

VERSION 1 - REVIEW

Reviewer 1

Name Kot, Jacek

Affiliation Medical University of Gdansk, National Center for Hyperbaric Medicine

Date 02-Nov-2024

COI None

The manuscript is a very good description of an elegant study on using HBOT for long-covid19.

While all scientific aspects of clinical research are correctly addressed, I strongly disagree with some of the conclusions presented by the Authors.

In lines 354-355, the Authors say, "We could not detect a statistically significant difference between HBOT and sham treatment in any of the primary or main secondary endpoints in the overall subject population."

Also, they state in line 82 that "The sample size was too small to detect conclusive benefits." and in lines 361 - 368 that "First, the sample size may be too small."

This is contradictory to the clinical trial project and the power analysis, which, in my opinion, were correctly planned and conducted.

The correct conclusion is that there was no observed effect of the procedure (HBOT) on the defined endpoints. This would mean that the effect is either smaller than expected (so the

larger population must be tested) or the procedure must be different. Both options could be hypotheses for the next studies but cannot be treated as CONCLUSIONS from this one.

The other comment is about the fatal case in the placebo group. This needs to be explained in more detail, including whether it directly relates to the therapeutic procedures, which means a hyperbaric session.

The description given below is vague:

Lines 426-428: Given the frailty of this group, it's possible that AEs occurred in the placebo group due to the effort of participation, by breathing non humidified air in sham treatment, or would be observed in those subjects even without treatment. "

Line 317 "One death (suspected suicide)"

Otherwise, the manuscript is excellent.

**Reviewer**	**2**
**Name**	**Efrati, Shai**
**Affiliation**	**Assaf-Harofeh) Medical Center**
**Date**	**04-Nov-2024**
**COI**	**i am medical advisor to AVIV scientific**

The article titled "Ten sessions of Hyperbaric Oxygen versus sham treatment in patients with Long COVID (HOT-LoCO): a randomized, placebo-controlled, double-blind, phase II trial" included 80 patients suffering from Long COVID. This study, initiated on September 15, 2021, was conducted before extensive knowledge on Long COVID was available, which may account for certain methodological limitations. Nevertheless, it offers important insights that any physician or researcher working with HBOT should consider.

Key Insights on HBOT and Sham Protocols:

The study's HBOT and sham protocols differ notably from protocols that have proven effective for Long COVID, with only ten sessions administered instead of the standard 40. In addition, the following two points should be clearly mention and discussed:

1. HBOT Protocol Variances: Medical oxygen (100%) was administered at 2.4 ATA for 90 minutes with two 5-minute air breaks using a re-breather mask. In contrast, the protocol found effective for Long COVID involves 2 ATA with air breaks every 20 minutes. This difference is significant, as 2.4 ATA may produce distinct biological effects. For example, while 2 ATA is associated with neurogenesis and regenerative benefits, 2.4 ATA may elevate toxicity risks and was nether demonstrated to be superior with regards to regeneration.

2. Sham Treatment Limitations: The sham protocol briefly raised pressure to 1.34 ATA before reducing and maintaining it at 1.2 ATA for 90 minutes. Previous studies, however, demonstrate that this level of pressurization cannot serve as a true sham, as tissue oxygenation increases by over 50% and stem cells proliferation is also extended for some degree. Thus, rather than a sham, the protocol used in this study functioned as a low-dose intervention.

These points are essential to address so that other investigators avoid similar methodological choices in future studies.

Treatment Duration Considerations:

The study’s findings highlight that ten HBOT sessions, while more convenient from an implementation perspective, are insufficient for achieving tissue regeneration. Furthermore, past research and clinical experience has shown that patients undergoing HBOT may experience fatigue or increased exhaustion during the initial 20–30 sessions, a reaction tied to the therapeutic process. Patients who experience such “worsening” of symptoms early in the treatment often achieve the greatest benefits, as observed in prior studies on fibromyalgia, PCS, and PTSD. Consequently, HBOT should not be initiated unless a patient can commit to a program of at least 40–60 sessions.

Conclusion and Recommendations:

The above considerations should be explicitly discussed in the study’s discussion and conclusion sections to prevent the replication of similar protocols without adjustment in future research.

**Reviewer**	**3**
**Name**	**Gorenstein, Scott**
**Affiliation**	**NYU Long Island School of Medicine, Surgery**
**Date**	**07-Nov-2024**
**COI**	**None**

A very well designed study and comprehensive manuscript. I applaud the undertaking and appreciated the scientific process to help provide a scientific basis for the use of HBO and long COVID.

**Reviewer**	**4**
**Name**	**Kelson, Zoe**

Affiliation **University of Exeter, Mathematics**

Date **12-Jan-2025**

COI **None**

This study aims to evaluate if ten hyperbaric oxygen treatments (HBOT) improve short- and long-term health related quality of life (HRQoL), symptoms, and physical performance in long COVID patients compared with placebo.

Reviewer comments:

"Trial registrations: ClinicalTrials.gov (NCT04842448), EudraCT (2021-000764-30)" [Abstract]

and

"The protocol was approved by The Swedish ethical review board (EPM no 2021-02634, amendment 2021-04572), approval 2021-05-25 and 2021-09-22 and The Swedish medical products agency (LV no 5.1-2020-36673), approval 2021-07-06. The trial was registered on ClinicalTrial.gov (NCT04842448), April 13, 2021, and on EudraCT (2021-000764-30), May 21, 2021, before start of the trial."

and

"The full protocol is published and available with open access."

and

"The Standard Operations Procedure (SOP) with details about the randomisation-blinding procedure used is published with the protocol and available online"

Thanks for providing a copy of the study protocol.

"A more technical and detailed elaboration of the principal features will be written in a separate Statistical Analysis Plan (SAP)."

Can the authors please provide a copy of this SAP?

"The trial was conducted in accordance with The Declaration of Helsinki, ICH-GCP, local and national regulations and is reported according to Consolidated Standards of Reporting Trials (CONSORT) Harms 2022 guidelines"

Thanks for providing a copy of the CONSORT checklist.

"Single-centre, parallel, randomised, placebo-controlled, double-blind trial conducted at Karolinska University Hospital, Sweden" [Abstract]

and

"HOT-LoCO (Hyperbaric Oxygen for Treatment of Long COVID syndrome) is an investigator initiated, randomised, placebo-controlled, double blind, parallel-arms, phase II clinical trial

that was conducted at the hyperbaric outpatient clinic at Karolinska University Hospital, Stockholm, Sweden."

A rigorous study design has been designed and followed.

"At 13 weeks, both groups showed improvement, with no significant difference between HBOT and placebo in PF (Least square mean difference between groups (LSD), 0.63 [95%CI - 7.04 to 8.29], p=0.87) and RP (LSD, 2.35 [95%CI - 5.95 to 10.66], p=0.57)." [Abstract]

The results have been presented accurately.

"Previously healthy subjects aged 18–60 years, diagnosed with long COVID were randomly assigned to ten HBOT or sham (placebo) treatments over six weeks." [Abstract]

and

"Between Sept 15, 2021, and June 20, 2023, 80 subjects (65 women, 15 men) were enrolled (40 in each group)." [Abstract]

and

"Patients were recruited trough long COVID outpatient clinics, directly or by advertisement through Svenska Covidföreningen"

and

"Between September 15, 2021, and June 20, 2023, 80 subjects were randomised. 61 subjects completed their ten treatments (29 in HBO 2 and 32 in placebo), four subjects had less than five treatments (three in HBOT and four in placebo) because of AEs and 15 subjects had five to nine treatments (seven in HBOT and eight in placebo), one subject in the HBOT group was given placebo."

and

"There may be a selection bias depending on this fact and there has also been a selection of patients that have been diagnosed with long COVID; only highly symptomatic cases were recruited"

Can the authors please further comment on whether the included cohort can be considered to be representative for generalisability of the study findings?

"A ten-point higher mean change in the HBOT group compared with the placebo group was considered as a clinically relevant difference. Sample size calculation was performed using a t test for independent groups with 80% power, and with a type I error rate of 0.05 (5%), assuming a common standard deviation (SD) of 15 known from prior studies, to detect a ten-point difference between groups. Power calculations indicated that at least 37 subjects per group were needed. Accounting for 5% dropout, we aimed to recruit 80 subjects. nQuery V.7 was used for sample size calculations."

The basis for the sample size calculation has been suitably communicated, in line with the study protocol.

"Randomisation (1:1) was done electronically, in blocks stratified by sex and disease severity" [Abstract]

and

"Eligible subjects were randomised in a 1:1 allocation, stratified by disease severity in relation to RAND-36 domains PF and PR (mean of PF and RP < or > 30, and sex in mixed block sizes 2:4:8 (blinded to investigators and personnel assessing outcome data) to either HBOT or placebo."

and

"primary endpoint is mean change from baseline to week 13 in the RAND-36 score of RP and PF respectively"

and

"for continuous outcome variables including the primary endpoint, analyses were performed using analysis of covariance (ANCOVA), unless otherwise specified, including stratification factors and treatment as fixed factors in the model. Estimates are presented using least square means for differences between treatment arms."

and

"All continuous variables are described using standard statistical measures, that is, number of observations (N), percentage (%), mean or median value, standard deviation (SD), and minimum and maximum values. Analysis for categorical data (yes/no) is presented as the proportion of subjects with the frequency of presence or absence, by treatment group of the characteristics of interest, and analysed using the Cochran-Mantel-Haenszel Chi-square test (CMH) χ2 test including stratification factors, where the parameter used for the statistical hypothesis testing will be the odds ratio (OR), as a measure of the relative difference in odds between treatment arms. An OR value greater than one indicates efficacy in favour of HBOT compared with placebo. "

The authors have applied suitable statistical methods, which satisfactorily adjust for the stratification variables, in line with the methods specified in the study protocol.

"Hypothesis testing for the primary endpoints were controlled at the type I error rate of 0.05 where adjustment for multiplicity was done using the Hochberg method"

Mutliple comparisons have been suitably corrected for by the authors, in line with the methods stated in the study protocol.

"Efficacy was analysed on an intention-to-treat basis. " [Abstract]

and

"Primary and main secondary endpoints were evaluated using the FAS population and sensitivity analyses was performed using the PPAS population."

The authors have suitable conducted a per protocol sensitivity analysis.

"For all analyses, the observed population has been used for evaluation, i.e., no imputation for missing data."

Can the authors please clarify how much missing covariate data there was?

Can it be assumed to be missing at random?

"For the primary endpoint efficacy analyses, missing data will be adequately imputed for all subjects in the FAS population. In addition, the observed cases population will be evaluated as a sensitivity analysis. For secondary endpoints, only observed data will be analysed" [protocol]

Can the authors please impute missing data in the primary analysis, as pre-specified in the study protocol?

"Subgroup analysis was done for males and females."

An insightful subgroup analysis has been pre-specified, performed and reported.

Can the authors please consider if additional exploratory subgroup analyses (e.g. by age or comorbidity) might be of interest?

"The following subgroups will be evaluated for this study: • Gender • Disease severity o RAND-36 RP and PF below 30 o RAND-36 RP and PF 30-50 o RAND-36 RP and PF above 50" [protocol]

Can the pre-specified subgroup analyses by disease severity please be undertaken and reported?

"The groups were balanced regarding demographics and baseline characteristics, suggesting a successful randomisation (Table 1)"

Perhaps differences between groups exist in smoking, snus, physical activity, vaccination status, and antibodies? Can the authors please consider if post-hoc exploratory analyses adjusting for these variables may be informative?

"In the long-term follow-up we observed a trend towards efficacy in PF at 52 weeks, but the differences were not statistically significant in the PPAS analysis (Table 3)."

and

"In addition, we observed a trend towards efficacy in the proportion of female subjects who received all ten treatments and had a clinically relevant increase of ten units from baseline in RP at 13 weeks (p=0.135) (FAS) (Supplemental table 6). Among female subjects there was also a trend towards statistically significant effect in subjects who had a clinically relevant increase of ten units from baseline in PF (p=0.096) at 52 weeks (FAS)."

Can the authors please refrain from inferring a 'trend' for statistically non-significant findings (where no difference can be concluded)?

"To validate the blinding procedure and sham-treatment as a placebo control, all subjects were asked to guess what treatment they received and upon unblinding the answers were compared to the actual treatment received. The McNemar´s test detected no association between treatment groups and guess of treatment (p=0.728) "

Can this test please be specified in the methods section?

Figure 2: Can statistical outcomes (i.e. precise p-values) please be shown on these plots?

"The sample size was too small to detect conclusive benefits" [strengths and limitations of this study]

and

" the sample size may be too small. We based our power calculation on similar studies, since no other RCTs with HBOT for long COVID were published when we started our trial. A new sample size calculation based on the trial´s results, with a mean difference of seven, and a common SD of 20 and an effect size of 0.350, implied that 130 patients are needed in each group to detect a significant difference between groups, using a t-test for independent groups with 80% power and significance level of 0.05 (5%)."

Can the authors please be cautious when suggesting the sample size was 'too small to detect conclusive benefits'.

The target sample size as per the rigorous a priori power calculation has been achieved.

The a priori sample size calculation was based on a 'clinically relevant difference' of ten (is seven still considered to be clinically relevant?).

Even if a larger sample size was recruited, the study may still not have provided 'conclusive benefits'.

"Ten sessions of HBOT in a mixed population of long COVID patients were not sufficient to detect an improvement in self-reported physical function evaluated by RAND-36, in the short term, but there may be a long-term effect. We discovered an important sex difference: ten sessions may be already effective for women who could also benefit additionally from more sessions. HBOT delivered with medical grade oxygen and hospital standards of chamber safety seems safe for previously healthy patients with long COVID. Further studies with subgroups for male and female population and a dose of more than ten sessions are required."

Can the conclusions please more accurately reflect that none of the pre-specified primary and secondary analyses found an effect, whilst exploratory post-hoc analyses have generated hypotheses that may warrant further investigation?

VERSION 1 - AUTHOR RESPONSE

Reviewer: 1
Dr. Jacek Kot, Medical University of Gdansk
Comments to the Author:
The manuscript is a very good description of an elegant study on using HBOT for long-covid19.

We thank the reviewer for a thorough review and constructive comments. We have responded to each comment to the best of our ability and in some cases explained the background and rationale for comments where we disagree.

1. While all scientific aspects of clinical research are correctly addressed, I strongly disagree with some of the conclusions presented by the Authors.

In lines 354-355, the Authors say, "We could not detect a statistically significant difference between HBOT and sham treatment in any of the primary or main secondary endpoints in the overall subject population."

Also, they state in line 82 that "The sample size was too small to detect conclusive benefits." and in lines 361 - 368 that "First, the sample size may be too small."

This is contradictory to the clinical trial project and the power analysis, which, in my opinion, were correctly planned and conducted.

Reply: Thank you for the comments. We have removed the statement "The sample size was too small to detect conclusive benefits." from the "Strengths and limitations of this study" section in line 82 and agree that this should not be a conclusion.

We beleive it is worth discussing as we have done in line 388-396. If the trial was correctly planned is open for discussion. When we designed the trial and conducted the power calculation in 2020-2021, the pandemic was still ongoing and there were no published reports on HBOT for Long COVID. However, we discussed with colleagues around the world that had tested HBOT for this novel condition and suggested benefits from unpublished data that corresponded to a clinically relevant change in SF-36 (in all essence same as RAND-36) with standard deviation (SD) 10-15. We used published non-randomised studies on Fibromyalgia and chronic fatigue syndrome that was the only similar condition at the time with published results. Since this was an unknown syndrome and HBOT requires plenty of resourses we decided to use a conservative minimal clinically important difference (MCID) of 10 points (Hays 2001), but some reserachers argue that an MCID for SF-36 is 3-5 points (Samsa 1999, Frendl 2014). Also, based on differences in baseline disease severity, compared to other published studies, we had a severely disabled study population, and the number of HBOT treatment sessions needed may be correlated to disease severity.

2. The correct conclusion is that there was no observed effect of the procedure (HBOT) on the defined endpoints. This would mean that the effect is either smaller than

expected (so the larger population must be tested) or the procedure must be different. Both options could be hypotheses for the next studies but cannot be treated as CONCLUSIONS from this one.

Reply: Many questions are still unanswered regarding the benefits of HBOT in general and specifically in long COVID, but the biggest problem with our trial is that the placebo effect was almost as high as the active treatment and may be considered clinically relevant. PF: Mean 9, SD(18.7) for HBOT vs 8.4 (15.9) for Sham and RP: 6.3 (20.2) for HBOT vs 3,8 (16.6) for Sham, in absolute numbers Intention to treat. One of the other reviewers also argue that the sham treatment may not be a proper placebo treatment. We believe that our conclusion is balanced since we observed a difference as expected close to our set MCID but but a much higher effect than expected for the Sham population and we disuss theese issues thoroughly in lines 398-434.

3. The other comment is about the fatal case in the placebo group. This needs to be explained in more detail, including whether it directly relates to the therapeutic procedures, which means a hyperbaric session.

Reply: Thank you for the comment. We have clarified that the suspected suicide was assessed as not related to the procedure In line 344. With respect for the relatives and other subjects that knew the dead subject we do not want to go into more details than "suspected suicide" in the main manuscript but the cause of death (traumatic hemorrage) and that it was unrelated to the intervention can be found in Supplementary Table 3c.

4. The description given below is vague:
Lines 426-428: Given the frailty of this group, it's possible that AEs occurred in the placebo group due to the effort of participation, by breathing non humidified air in sham treatment, or would be observed in those subjects even without treatment. "

Reply: We agree that it may be a vague if one have not encountered this group of severely disabled patients, they are extremely sensitive to any type of stress. Some patients will have post exertional malaise by taking a shower, changing clothes and may have a serious back-lash from coming for an appointment in the hospital. It is possible that the well established "placebo treatment" is infact not placebo for this group of patients. We have added "There is also a possibility that the Sham treatment is sufficient stress for this group of patients to have a physiological effect, which may explain the unexpected small difference between active treatment and sham treatment" in line 458-461.

5. Line 317 "One death (suspected suicide)"

Reply: Please see our previous response: We have clarified that the suspected suicide was assessed as not related to the procedure In line 344. With respect for the relatives and other subjects that knew the dead subject we do not want to go into more details than "suspected suicide" in the main manuscript but the cause of death (traumatic hemorrage) and that it was unrelated to the intervention can be found in Supplementary

Table 3c.

6. Otherwise, the manuscript is excellent.

Thank you again for taking the time and effort to read and comment on our manuscript!

Reviewer: 2
Prof. Shai Efrati, Assaf-Harofeh) Medical Center, Tel-Aviv University, Development Unit, Tel-Aviv University
Comments to the Author:
The article titled “Ten sessions of Hyperbaric Oxygen versus sham treatment in patients with Long COVID (HOT-LoCO): a randomized, placebo-controlled, double-blind, phase II trial” included 80 patients suffering from Long COVID. This study, initiated on September 15, 2021, was conducted before extensive knowledge on Long COVID was available, which may account for certain methodological limitations. Nevertheless, it offers important insights that any physician or researcher working with HBOT should consider.

We thank the reviewer for taking the time to read our manuscript and provide a thorough review with relevant and constructive comments. We have responded to each comment to the best of our ability and in some cases explained the background and rationale for comments where we disagree.

Key Insights on HBOT and Sham Protocols:
The study’s HBOT and sham protocols differ notably from protocols that have proven effective for Long COVID, with only ten sessions administered instead of the standard 40. In addition, the following two points should be clearly mention and discussed:

1. HBOT Protocol Variances: Medical oxygen (100%) was administered at 2.4 ATA for 90 minutes with two 5-minute air breaks using a re-breather mask. In contrast, the protocol found effective for Long COVID involves 2 ATA with air breaks every 20 minutes. This difference is significant, as 2.4 ATA may produce distinct biological effects. For example, while 2 ATA is associated with neurogenesis and regenerative benefits, 2.4 ATA may elevate toxicity risks and was nether demonstrated to be superior with regards to regeneration.

Reply: Thank you for this relevant comment regarding the “optimal dose” for each session. Many centers around the world, including ours, still use 2.4 ATA based on old dogmas and other indications with chronic inflammation where our protocol has been shown to be effective, such as for late radiation tissue injury (Clarke 2008, Oscarsson 2019). It is quite possible that the 2.4 ATA with two air breaks is an unnecessary high dose. In line 393-396 we have written about the evidence for HBOT in long COVID which include that 40 sessions of HBOT at 2.0 ATA has been shown effective. We have added details of the protocol “five days a week, 90 minutes with air-breaks every 20 minutes” .

2. Sham Treatment Limitations: The sham protocol briefly raised pressure to 1.34 ATA before reducing and maintaining it at 1.2 ATA for 90 minutes. Previous studies, however, demonstrate that this level of pressurization cannot serve as a true sham, as tissue oxygenation increases by over 50% and stem cells proliferation is also extended for some degree. Thus, rather than a sham, the protocol used in this study functioned as a low-dose intervention.

Reply: This is also a very relevant comment. We have added a sentence in line 458-461 "There is also a possibility that the Sham treatment is sufficient stress for this group of patients to have a physiological effect, which may explain the unexpected small difference between active treatment and sham treatment and the similarities in AEs."

These points are essential to address so that other investigators avoid similar methodological choices in future studies.

3. Treatment Duration Considerations:
The study's findings highlight that ten HBOT sessions, while more convenient from an implementation perspective, are insufficient for achieving tissue regeneration. Furthermore, past research and clinical experience has shown that patients undergoing HBOT may experience fatigue or increased exhaustion during the initial 20–30 sessions, a reaction tied to the therapeutic process. Patients who experience such "worsening" of symptoms early in the treatment often achieve the greatest benefits, as observed in prior studies on fibromyalgia, PCS, and PTSD. Consequently, HBOT should not be initiated unless a patient can commit to a program of at least 40–60 sessions.

Reply: Thank you for this comment. We are currently looking at the possibility of conducting a new trial to confirm previous results with 40 sessions and also including 60 sessions that has been shown beneficial for PTSD. However, there is still clinical equipoise regarding the "optimal dose" and the dose may need individual tailoring to be cost efficient. We have added this to the discussion in lines 414-416 and added a reference.

4. Conclusion and Recommendations:
The above considerations should be explicitly discussed in the study's discussion and conclusion sections to prevent the replication of similar protocols without adjustment in future research.

Reply: We hope that the revisions made in the discussion is satisfying the expectations from the reviewers. We have also discussed dosing in detail line 398-427. To conclude the optimal dose, non-inferiority trials with different doses are needed. There may also be a need for individual dosing to make HBOT cost efficient for the general population.

Reviewer: 3

Dr. Scott Gorenstein, NYU Long Island School of Medicine
Comments to the Author:
A very well designed study and comprehensive manuscript. I applaud the undertaking and appreciated the scientific process to help provide a scientific basis for the use of HBO and long COVID.

We thank the reviewer for taking the time to read our manuscript.
Thank you for your support!

Reviewer: 4
Prof. Zoe Kelson, University of Exeter
Comments to the Author:
This study aims to evaluate if ten hyperbaric oxygen treatments (HBOT) improve short- and long-term health related quality of life (HRQoL), symptoms, and physical performance in long COVID patients compared with placebo.

We thank the reviewer for taking the time and effort to read and comment on our manuscript. We have commented the questions/requests and have tried to adress them at the best of our ability. Where we have decided not to fulfil the request we have provided a rationale for our decision.

Reviewer comments:

"Trial registrations: ClinicalTrials.gov (NCT04842448), EudraCT (2021-000764-30)" [Abstract]
and
"The protocol was approved by The Swedish ethical review board (EPM no 2021-02634, amendment 2021-04572), approval 2021-05-25 and 2021-09-22 and The Swedish medical products agency (LV no 5.1-2020-36673), approval 2021-07-06. The trial was registered on ClinicalTrial.gov (NCT04842448), April 13, 2021, and on EudraCT (2021-000764-30), May 21, 2021, before start of the trial."
and
"The full protocol is published and available with open access."
and
"The Standard Operations Procedure (SOP) with details about the randomisation-blinding procedure used is published with the protocol and available online"

1. Thanks for providing a copy of the study protocol.
Reply: Thank you for a thorough review.

"A more technical and detailed elaboration of the principal features will be written in a separate Statistical Analysis Plan (SAP)."

2. Can the authors please provide a copy of this SAP?
Reply: Thank you for the comment, the SAP is now included for review and will be published on clinical trials when the manuscript is published. If the editor finds it

relevant can also be published with the manuscript if accepted.

"The trial was conducted in accordance with The Declaration of Helsinki, ICH-GCP, local and national regulations and is reported according to Consolidated Standards of Reporting Trials (CONSORT) Harms 2022 guidelines"

3. Thanks for providing a copy of the CONSORT checklist.
Reply: Thank you for reviewing.

"Single-centre, parallel, randomised, placebo-controlled, double-blind trial conducted at Karolinska University Hospital, Sweden" [Abstract]
and
"HOT-LoCO (Hyperbaric Oxygen for Treatment of Long COVID syndrome) is an investigator initiated, randomised, placebo-controlled, double blind, parallel-arms, phase II clinical trial that was conducted at the hyperbaric outpatient clinic at Karolinska University Hospital, Stockholm, Sweden."

4. A rigorous study design has been designed and followed.
Reply: Thank you for your comment. We are conducting clinical trials with HBOT in compliance with ICH-GCP to ensure patient safety and integrity, and quality of data.

"At 13 weeks, both groups showed improvement, with no significant difference between HBOT and placebo in PF (Least square mean difference between groups (LSD), 0.63 [95%CI -7.04 to 8.29], p=0.87) and RP (LSD, 2.35 [95%CI - 5.95 to 10.66], p=0.57)." [Abstract]

5. The results have been presented accurately.
Reply: Thank you.

"Previously healthy subjects aged 18–60 years, diagnosed with long COVID were randomly assigned to ten HBOT or sham (placebo) treatments over six weeks." [Abstract]
and
"Between Sept 15, 2021, and June 20, 2023, 80 subjects (65 women, 15 men) were enrolled (40 in each group)." [Abstract]
and
"Patients were recruited trough long COVID outpatient clinics, directly or by advertisement through Svenska Covidföreningen"
and
"Between September 15, 2021, and June 20, 2023, 80 subjects were randomised. 61 subjects completed their ten treatments (29 in HBO 2 and 32 in placebo), four subjects had less than five treatments (three in HBOT and four in placebo) because of AEs and 15 subjects had five to nine treatments (seven in HBOT and eight in placebo), one subject in the HBOT group was given placebo."
and
"There may be a selection bias depending on this fact and there has also been a

selection of patients that have been diagnosed with long COVID; only highly symptomatic cases were recruited"

6. Can the authors please further comment on whether the included cohort can be considered to be representative for generalisability of the study findings?
Reply: Thank you for this relevant question. We have clarified that "For the above reasons our cohort may not be representative of the general post COVID-19 condition population, but the problem with an increasing number of patients is a major socioeconomic burden that continues to grow." In lines 450-452

"A ten-point higher mean change in the HBOT group compared with the placebo group was considered as a clinically relevant difference. Sample size calculation was performed using a t test for independent groups with 80% power, and with a type I error rate of 0.05 (5%), assuming a common standard deviation (SD) of 15 known from prior studies, to detect a ten-point difference between groups. Power calculations indicated that at least 37 subjects per group were needed. Accounting for 5% dropout, we aimed to recruit 80 subjects. nQuery V.7 was used for sample size calculations."

7. The basis for the sample size calculation has been suitably communicated, in line with the study protocol.
Reply: Thank you for contributing with your statistical expertise.

"Randomisation (1:1) was done electronically, in blocks stratified by sex and disease severity" [Abstract]
and
"Eligible subjects were randomised in a 1:1 allocation, stratified by disease severity in relation to RAND-36 domains PF and PR (mean of PF and RP < or > 30, and sex in mixed block sizes 2:4:8 (blinded to investigators and personnel assessing outcome data) to either HBOT or placebo."
and
"primary endpoint is mean change from baseline to week 13 in the RAND-36 score of RP and PF respectively"
and
"for continuous outcome variables including the primary endpoint, analyses were performed using analysis of covariance (ANCOVA), unless otherwise specified, including stratification factors and treatment as fixed factors in the model. Estimates are presented using least square means for differences between treatment arms."
and
"All continuous variables are described using standard statistical measures, that is, number of observations (N), percentage (%), mean or median value, standard deviation (SD), and minimum and maximum values. Analysis for categorical data (yes/no) is presented as the proportion of subjects with the frequency of presence or absence, by treatment group of the characteristics of interest, and analysed using the Cochran-Mantel-Haenszel Chi-square test (CMH) $\chi 2$ test including stratification factors, where the parameter used for the statistical hypothesis testing will be the odds ratio (OR), as a measure of the relative difference in odds between treatment arms. An OR value greater than one indicates efficacy in favour of HBOT compared with placebo. "

8. The authors have applied suitable statistical methods, which satisfactorily adjust for the stratification variables, in line with the methods specified in the study protocol.
Reply: Thank you for contributing with your statistical expertise.

"Hypothesis testing for the primary endpoints were controlled at the type I error rate of 0.05 where adjustment for multiplicity was done using the Hochberg method"
Mutliple comparisons have been suitably corrected for by the authors, in line with the methods stated in the study protocol.

"Efficacy was analysed on an intention-to-treat basis. " [Abstract]
and
"Primary and main secondary endpoints were evaluated using the FAS population and sensitivity analyses was performed using the PPAS population."

9. The authors have suitable conducted a per protocol sensitivity analysis.
Reply: Thank you for contributing with your statistical expertise.

"For all analyses, the observed population has been used for evaluation, i.e., no imputation for missing data."

10. Can the authors please clarify how much missing covariate data there was?
Can it be assumed to be missing at random?
Reply: Thank you for the relevant question. There were in general very little missing data and monitoring was performed on 13 occasions (10 unblinded and 3 blinded) by independent monitors from KTA. The covariates Sex and Disease severity was predefined and there were complete.

"For the primary endpoint efficacy analyses, missing data will be adequately imputed for all subjects in the FAS population. In addition, the observed cases population will be evaluated as a sensitivity analysis. For secondary endpoints, only observed data will be analysed" [protocol]

11. Can the authors please impute missing data in the primary analysis, as pre-specified in the study protocol?
Reply: There was only one subject with missing data in the follow-up and analysis of the primary endpoint: the subject that died before follow up. We analysed data as we have decided in the SAP, i.e., to exclude that subject, only analysing observed cases. This single missing data point 1 out of 80, could maximally have very little impact on the interpretations and conclusions of the results.

"Subgroup analysis was done for males and females."

12. An insightful subgroup analysis has been pre-specified, performed and reported.
Can the authors please consider if additional exploratory subgroup analyses (e.g. by age or comorbidity) might be of interest?

Reply: Age may be of interest but the variation in age was quite small 41.3 (9.15) and the treatment groups were very similar, 41.1 (HBOT) vs 41.4 (Placebo). Unfortunately we do not have the resources to do such a post-hoc analysis at this stage. Of course it could have been of interest to explore that, but we do not have any good reference suggesting how to divide age into age groups for subgroup analysis.
Comorbidities were very few since we only included "previously healthy subjects" and would therefore most likely not be relevant.

"The following subgroups will be evaluated for this study: • Gender • Disease severity o RAND-36 RP and PF below 30 o RAND-36 RP and PF 30-50 o RAND-36 RP and PF above 50" [protocol]

13. Can the pre-specified subgroup analyses by disease severity please be undertaken and reported?
Reply: We did not have any subjects with RAND-36 above 50 but the other two categories were included. There were 66 subjects in the 0-30 subgroup and only 14 in the 30-50 subgroup. The latter group were too small to allow for any robust analysis and findings. However, there were no significant differences within the subgroups of disease severity. We decided to not include these results in the manuscript.

"The groups were balanced regarding demographics and baseline characteristics, suggesting a successful randomisation (Table 1)"

14. Perhaps differences between groups exist in smoking, snus, physical activity, vaccination status, and antibodies? Can the authors please consider if post-hoc exploratory analyses adjusting for these variables may be informative?

Reply: Thank you for the interesting input for explanatory factors for the correlation/covariation to the primary endpoint. The cohort in our study included no smokers. Snus users and physical activity impact on the primary endpoint may be interesting to study as sub group analyses. However, this is out of scope for this randomised controlled trial, where these subgroups are expected to be fairly balanced because of its nature. Adequate adjustments for several factors in a multivariate analysis would be difficult in this small data set, which is tailored for the aim of this clinical trial. A much larger data set would be needed to perform the exploratory analyses.

"In the long-term follow-up we observed a trend towards efficacy in PF at 52 weeks, but the differences were not statistically significant in the PPAS analysis (Table 3)."
and
"In addition, we observed a trend towards efficacy in the proportion of female subjects who received all ten treatments and had a clinically relevant increase of ten units from baseline in RP at 13 weeks (p=0.135) (FAS) (Supplemental table 6). Among female subjects there was also a trend towards statistically significant effect in subjects who had a clinically relevant increase of ten units from baseline in PF (p=0.096) at 52 weeks (FAS)."

15. Can the authors please refrain from inferring a 'trend' for statistically non-significant findings (where no difference can be concluded)?

Reply: We agree that this should be excluded from conclusions, we have excluded "trend" used in line 79, and have clarified that this is an exploratory analysis in lines 333 and 469-470. However we would like to explain the rationale for using it in line 331, 365 and 367. The rationale for us using "trend" is that we have seen this sex-difference in a previous clinical trial (Kjellberg 2023) and dose-adjustments according to sex differences has not previously been considered in the field of HBOT. This is in our opinion the most interesting exploratory finding in the results and may be relevant for future individualised protocols.
Since the efficacy in a clinical trial conducted in compliance with ICH-GCP is purely based on the primary endpoint, and all secondary endpoints are of exploratory character, we find it relevant to include this "trend" in the results and discussion as a hypothesis generating finding. When further analysing this "trend" in the per-protocol population the results were statistically significant, and the effect seems to be "dose-dependent" as suggested in Supplementary table 4a and 4b. Taken toghether these findings suggest that this "trend" may be relevant.

"To validate the blinding procedure and sham-treatment as a placebo control, all subjects were asked to guess what treatment they received and upon unblinding the answers were compared to the actual treatment received. The McNemar´s test detected no association between treatment groups and guess of treatment (p=0.728) "
16. Can this test please be specified in the methods section?

Reply: Thank you for this comment. We have specified in the method section that: "The McNemar's test was used to assess the association between the guessed and actual treatments (e.g., proportion of correct versus incorrect guesses). A high p-value suggests that the blinding procedure was effective." In lines 288-290

17. Figure 2: Can statistical outcomes (i.e. precise p-values) please be shown on these plots?

Reply: Normally, we would like to avoid duplicates in terms of numbers, p-values in our manuscript, where these data are presented under the results of the primary endpoint and in table 3. The rationale for Figure 2 is to give a graphical understanding of the primary endpoints. But of course, we have followed the reviewers instructions.

"The sample size was too small to detect conclusive benefits" [strengths and limitations of this study]
and
" the sample size may be too small. We based our power calculation on similar studies, since no other RCTs with HBOT for long COVID were published when we started our trial. A new sample size calculation based on the trial´s results, with a mean difference

of seven, and a common SD of 20 and an effect size of 0.350, implied that 130 patients are needed in each group to detect a significant difference between groups, using a t-test for independent groups with 80% power and significance level of 0.05 (5%)."

18. Can the authors please be cautious when suggesting the sample size was 'too small to detect conclusive benefits'.
a. The target sample size as per the rigorous a priori power calculation has been achieved.
b. The a priori sample size calculation was based on a 'clinically relevant difference' of ten (is seven still considered to be clinically relevant?).
c. Even if a larger sample size was recruited, the study may still not have provided 'conclusive benefits'.

Reply: Thank you for relevant questions and comments.

a) We have removed the statement from “strengths and limitations” line 82
b) When we designed the trial and conducted the power calculation in December 2020, the pandemic was still ongoing and there were no published reports on HBOT for Long COVID. However, we discussed with colleaguse around the world that had tested HBOT for this novel condition and suggested benefits from unpublished data that corresponded to a clinically relevant change in SF-36 (in all essence same as RAND-36) with standard deviation (SD) 10-15. We used published non-randomised studies on Fibromyalgia and chronic fatigue syndrome, that was the only similar condition at the time with published results. We also did not expect the placebo treatment to be “clinically significant”. Since this was an unknown syndrome and HBOT requires plenty of resourses we decided to use a conservative minimallt clinically important difference (MCID) of 10 points (Hays 2001), but some reserachers argue that an MCID for SF-36 is 3-5 points (Samsa 1999, Frendl 2014). Also, the study population recruited showed to be much more severe in terms of the baseline status in terms of disease severity measured as RAND-36 RP and PF. Therefore, the clinical relevant change to be possible to achive in these endpoints may be somewhat different from what the previous studies has published.
c) True. However, with the results from the current trial and the other RCT published, other researchers will have a better chance of conducting a better sample size calculation.

"Ten sessions of HBOT in a mixed population of long COVID patients were not sufficient to detect an improvement in self-reported physical function evaluated by RAND-36, in the short term, but there may be a long-term effect. We discovered an important sex difference: ten sessions may be already effective for women who could also benefit additionally from more sessions. HBOT delivered with medical grade oxygen and hospital standards of chamber safety seems safe for previously healthy patients with long COVID. Further studies with subgroups for male and female population and a dose of more than ten sessions are required."

19. Can the conclusieons please more accurately reflect that none of the pre-specified primary and secondary analyses found an effect, whilst exploratory post-hoc analyses have generated hypotheses that may warrant further investigation?

Thank you for this relevant comment. We have amended the conclusions to: “Ten sessions of HBOT in a mixed population of long COVID patients were not sufficient to detect an statistically significant improvement in self-reported physical function evaluated by RAND-36, or any of the main secondary outcomes in the short term. Exploratory post-hoc analysis generated a hypothesis for a possible long-term effect. We discovered an important sex difference: ten sessions may be effective for women. HBOT delivered with medical grade oxygen and hospital standards of chamber safety seems safe for previously healthy patients with long COVID. Further studies with subgroups for male and female population and a dose of more than ten sessions are required.”

Reviewer: 1 competing interests.: Not applicable

Reviewer: 2 competing interests.: i am medical advisor to AVIV scientific

Reviewer: 3 competing interests.: n/a

Reviewer: 4 competing interests.: Not applicable

VERSION 2 - REVIEW

**Reviewer**	**1**
**Name**	**Kot, Jacek**
**Affiliation**	**Medical University of Gdansk, National Center for Hyperbaric Medicine**
**Date**	**14-Mar-2025**
**COI**	

All my concerns were explained - thank you. Congratultations for the study.

**Reviewer**	**2**
**Name**	**Efrati, Shai**
**Affiliation**	**Assaf-Harofeh) Medical Center**

Date **28-Feb-2025**

COI

The authors addressed the reviewers comments

Reviewer **4**

Name **Kelson, Zoe**

Affiliation **University of Exeter, Mathematics**

Date **06-Mar-2025**

COI

Many thanks to the authors for the responses and revisions.

The following reviewer requests are raised:

Author responses:

"There were in general very little missing data and monitoring was performed on 13 occasions (10 unblinded and 3 blinded) by independent monitors from KTA. The covariates Sex and Disease severity was predefined and there were complete."

and

"There was only one subject with missing data in the follow-up and analysis of the primary endpoint: the subject that died before follow up. We analysed data as we have decided in the SAP, i.e., to exclude that subject, only analysing observed cases. This single missing data point 1 out of 80, could maximally have very little impact on the interpretations and conclusions of the results. "

and

"We did not have any subjects with RAND-36 above 50 but the other two categories were included. There were 66 subjects in the 0-30 subgroup and only 14 in the 30-50 subgroup. The latter group were too small to allow for any robust analysis and findings. However, there were no significant differences within the subgroups of disease severity. We decided to not include these results in the manuscript."

and

"Adequate adjustments for several factors in a multivariate analysis would be difficult in this small data set, which is tailored for the aim of this clinical trial. A much larger data set would be needed to perform the exploratory analyses."

Reviewer request:

Can the above responses to reviewer please be further clarified in the article?

Author response:

"...we would like to explain the rationale for using it [trend] in line 331, 365 and 367. The rationale for us using “trend” is that we have seen this sexdifference in a previous clinical trial (Kjellberg 2023) and dose-adjustments according to sex differences has not previously been considered in the field of HBOT. This is in our opinion the most interesting exploratory finding in the results and may be relevant for future individualised protocols. Since the efficacy in a clinical trial conducted in compliance with ICH-GCP is purely based on the primary endpoint, and all secondary endpoints are of exploratory character, we find it relevant to include this “trend” in the results and discussion as a hypothesis generating finding. When further analysing this “trend” in the per-protocol population the results were statistically significant, and the effect seems to be “dose dependent” as suggested in Supplementary table 4a and 4b. Taken toghether these findings suggest that this “trend” may be relevant. "

Article text:

"Subgroup comparison in the primary endpoint reveal that there was an almost statistically significant difference between treatment groups among female subjects, in SAF, PPAS and PPS10 analysis populations; FAS: PF (n=32 HBOT n=32 placebo): 2.038 (4.43) -6.86 to 10.91, p = 0.648, RP: 7.019 (3.76) -0.509 to 14.55, p = 0.067, PPAS: RP (n=28+32) with at least 5 treatments 7.980 (4.006) - 0.038 to 15.999, p=0.051, PPS10: RP (n=24+29) with at least 10 treatments 9.059 (4.566) -0.11 to 18.23, p=0.053

In addition, we observed a trend towards efficacy in the proportion of female subjects who received all ten treatments and had a clinically relevant increase of ten units from baseline in RP at 13 weeks (p=0.135) (FAS) (Supplemental table 7). Among female subjects there was also a trend towards statistically significant effect in subjects who had a clinically relevant increase of ten units from baseline in PF (p=0.096) at 52 weeks (FAS) (Supplemental table 7). For male subjects, there were no statistically significant difference between treatment groups, PF: -7.776 (6.47) -18.87 to 9.32, p = 0.475, RP (n=8 HBOT n=7 placebo): -17.53 (15.95) – 49.34 to 14.26, p = 0.253."

Reviewer requests:

It is not clear where supplementary tables 4a and 4b which the authors refer to are in the submission?

The authors reasoning and rationale is appreciated, however the use of 'trend' is currently associated with a non-significant findings in the article text (referring to Table 3 and Supplemental table 7). Can the authors please ensure that non-significant outcomes are reported as no evidence for a difference, and then go on to discuss the significant outcomes for the exploratory per-protocol analyses (showing supplementary tables 4a and 4b in support of this) which give rise to the potentially important hypothesis of sex differences?

Furthermore, can the authors please refrain from inferring 'an almost statistically significant difference' when p-values are close to but above 0.05? These are non-significant outcomes, with no difference between groups evidenced.

However, given the citation provided by the authors and the borderline nature of some of the outcomes, the authors can conclude in the discussion that sex differences may warrant further investigation in future studies.

Author amended conclusion text:

"Ten sessions of HBOT in a mixed population of long COVID patients were not sufficient to detect an statistically significant improvement in self-reported physical function evaluated by RAND-36, or any of the main secondary outcomes in the short term. Exploratory post-hoc analysis generated a hypothesis for a possible long-term effect. We discovered an important sex difference: ten sessions may be effective for women. HBOT delivered with medical grade oxygen and hospital standards of chamber safety seems safe for previously healthy patients with long COVID. Further studies with subgroups for male and female population and a dose of more than ten sessions are required."

Reviewer request:

Thanks for amending the conclusions.

In addition to this revision, can the authors please refrain from implying that sample size prevented signficance (as previously discussed in a comment with the authors, we agree that even if a larger sample size was recruited, the study may still not have provided conclusive benefits). Specifically, please can the authors remove the phrase 'were not sufficient to', and replace this with 'did not'?

Furthermore, can the authors please add 'in the per protocol population' to the end of 'We discovered an important sex difference', for clarity, transparency, and accurate interpretation?

VERSION 2 - AUTHOR RESPONSE

Reviewer: 1
Dr. Jacek Kot, Medical University of Gdansk
Comments to the Author:
All my concerns were explained - thank you. Congratultations for the study.
Thank you again for your time and effort!

Reviewer: 2
Prof. Shai Efrati, Assaf-Harofeh) Medical Center, Tel-Aviv University, Development Unit,

Tel-Aviv University
Comments to the Author:
The authors addressed the reviewers comments
Thank you again for your time and effort!

Reviewer: 4
Prof. Zoe Kelson, University of Exeter
Comments to the Author:
Many thanks to the authors for the responses and revisions.
The following reviewer requests are raised:
Thank you again for your time and effort to help us to improve our manuscript. We have addressed your additional/outstanding questions and requests in point by point responses and made requested changes in the manuscript.

Author responses:
"There were in general very little missing data and monitoring was performed on 13 occasions (10 unblinded and 3 blinded) by independent monitors from KTA. The covariates Sex and Disease severity was predefined and there were complete."
and
"There was only one subject with missing data in the follow-up and analysis of the primary endpoint: the subject that died before follow up. We analysed data as we have decided in the SAP, i.e., to exclude that subject, only analysing observed cases. This single missing data point 1 out of 80, could maximally have very little impact on the interpretations and conclusions of the results. "
and
"We did not have any subjects with RAND-36 above 50 but the other two categories were included. There were 66 subjects in the 0-30 subgroup and only 14 in the 30-50 subgroup. The latter group were too small to allow for any robust analysis and findings. However, there were no significant differences within the subgroups of disease severity. We decided to not include these results in the manuscript."
and
"Adequate adjustments for several factors in a multivariate analysis would be difficult in this small data set, which is tailored for the aim of this clinical trial. A much larger data set would be needed to perform the exploratory analyses."
Reviewer request:
Can the above responses to reviewer please be further clarified in the article?
Response 1: Thank you for this comment. This has been clarified in line 322-325 in the results and line 434-438 in the discussion.

Author response:
"...we would like to explain the rationale for using it [trend] in line 331, 365 and 367. The rationale for us using “trend” is that we have seen this sexdifference in a previous clinical trial (Kjellberg 2023) and dose-adjustments according to sex differences has not previously been considered in the field of HBOT. This is in our opinion the most interesting exploratory finding in the results and may be relevant for future individualised protocols. Since the efficacy in a clinical trial conducted in compliance with ICH-GCP is purely based on the primary endpoint, and all secondary endpoints are

of exploratory character, we find it relevant to include this “trend” in the results and discussion as a hypothesis generating finding. When further analysing this “trend” in the per-protocol population the results were statistically significant, and the effect seems to be “dose dependent” as suggested in Supplementary table 4a and 4b. Taken toghether these findings suggest that this “trend” may be relevant. "

Article text:

"Subgroup comparison in the primary endpoint reveal that there was an almost statistically significant difference between treatment groups among female subjects, in SAF, PPAS and PPS10 analysis populations; FAS: PF (n=32 HBOT n=32 placebo): 2.038 (4.43) -6.86 to 10.91, p = 0.648, RP: 7.019 (3.76) -0.509 to 14.55, p = 0.067, PPAS: RP (n=28+32) with at least 5 treatments 7.980 (4.006) - 0.038 to 15.999, p=0.051, PPS10: RP (n=24+29) with at least 10 treatments 9.059 (4.566) -0.11 to 18.23, p=0.053

In addition, we observed a trend towards efficacy in the proportion of female subjects who received all ten treatments and had a clinically relevant increase of ten units from baseline in RP at 13 weeks (p=0.135) (FAS) (Supplemental table 7). Among female subjects there was also a trend towards statistically significant effect in subjects who had a clinically relevant increase of ten units from baseline in PF (p=0.096) at 52 weeks (FAS) (Supplemental table 7). For male subjects, there were no statistically significant difference between treatment groups, PF: -7.776 (6.47) -18.87 to 9.32, p = 0.475, RP (n=8 HBOT n=7 placebo): -17.53 (15.95) – 49.34 to 14.26, p = 0.253."

Reviewer requests:

It is not clear where supplementary tables 4a and 4b which the authors refer to are in the submission?

Response 2: Sorry for the confusion on our side. Table 4a is now Table 6 and 4b was removed. We also noted some typos in Supplementary Table 6 and Table 7 where we have amended the numbers in “Total”.

The authors reasoning and rationale is appreciated, however the use of 'trend' is currently associated with a non-significant findings in the article text (referring to Table 3 and Supplemental table 7). Can the authors please ensure that non-significant outcomes are reported as no evidence for a difference, and then go on to discuss the significant outcomes for the exploratory per-protocol analyses (showing supplementary tables 4a and 4b in support of this) which give rise to the potentially important hypothesis of sex differences?

Furthermore, can the authors please refrain from inferring 'an almost statistically significant difference' when p-values are close to but above 0.05? These are non-significant outcomes, with no difference between groups evidenced.

However, given the citation provided by the authors and the borderline nature of some of the outcomes, the authors can conclude in the discussion that sex differences may warrant further investigation in future studies.

Response 3: We agree with these requests. We have amended the manuscript according to the reviewer´s requests. “Trend towards efficacy” was removed in line 345, and “almost” in line 373 and 379. We have claified that the observed difference in favour of HBOT was not statistically significant.

Author amended conclusion text:

"Ten sessions of HBOT in a mixed population of long COVID patients were not

sufficient to detect an statistically significant improvement in self-reported physical function evaluated by RAND-36, or any of the main secondary outcomes in the short term. Exploratory post-hoc analysis generated a hypothesis for a possible long-term effect. We discovered an important sex difference: ten sessions may be effective for women. HBOT delivered with medical grade oxygen and hospital standards of chamber safety seems safe for previously healthy patients with long COVID. Further studies with subgroups for male and female population and a dose of more than ten sessions are required."

Reviewer request:
Thanks for amending the conclusions.
In addition to this revision, can the authors please refrain from implying that sample size prevented significance (as previously discussed in a comment with the authors, we agree that even if a larger sample size was recruited, the study may still not have provided conclusive benefits). Specifically, please can the authors remove the phrase 'were not sufficient to', and replace this with 'did not'?
Response 4: We have changed the phrase to ‘did not’ as requested.

Furthermore, can the authors please add 'in the per protocol population' to the end of 'We discovered an important sex difference', for clarity, transparency, and accurate interpretation?
Response 5: We have added ‘in the per protocol population’ as requested

---

Reviewer: 1 competing interests.: Not applicable

Reviewer: 2 competing interests.: chair of the medical advisory board and shareholder in AVIV scientific

Reviewer: 4 competing interests.: Not applicable
